# Molecular signature of an ancient organizer regulated by Wnt/β-catenin signalling during primary body axis patterning in *Hydra*

**DOI:** 10.1038/s42003-019-0680-3

**Published:** 2019-11-26

**Authors:** Puli Chandramouli Reddy, Akhila Gungi, Suyog Ubhe, Saurabh J. Pradhan, Amol Kolte, Sanjeev Galande

**Affiliations:** Centre of Excellence in Epigenetics, Department of Biology, Indian Institute of Science Education and Research, Pune, 411008 India

**Keywords:** Evolutionary developmental biology, Pattern formation

## Abstract

Wnt/β-catenin signalling has been shown to play a critical role during head organizer formation in *Hydra*. Here, we characterized the Wnt signalling regulatory network involved in formation of the head organizer. We found that Wnt signalling regulates genes that are important in tissue morphogenesis. We identified that majority of transcription factors (TFs) regulated by Wnt/β-catenin signalling belong to the homeodomain and forkhead families. Silencing of *Margin*, one of the Wnt regulated homeodomain TFs, results in loss of the ectopic tentacle phenotype typically seen upon activation of Wnt signalling. Furthermore, we show that the *Margin* promoter is directly bound and regulated by β-catenin. Ectopic expression of *Margin* in zebrafish embryos results in body axis abnormalities suggesting that *Margin* plays a role in axis patterning. Our findings suggest that homeobox TFs came under the regulatory umbrella of Wnt/β-catenin signalling presumably resulting in the evolution of primary body axis in animal phyla.

## Abstract (web summary)

Puli Chandramouli Reddy et al. characterize the Wnt signalling regulatory network involved in the formation of head organization in *Hydra*. They find that *Margin*, a Wnt-regulated homeodomain transcription factor plays a key role in body axis patterning.

## Introduction

The palaeontological records indicate that animals with primary body axis existed in the Ediacaran period (635–541 million years ago)^1,2^. Phyla such as Porifera, Ctenophora and Cnidaria which diverged during the Ediacaran–Cambrian transition might hold clues about the molecular mechanisms determining the primitive body axis. Homeodomain-containing proteins evolved with critical roles in animal body patterning and development^3,4^. Among these, Hox genes have been postulated to play important roles in determining the axis polarity based on their colinear expression and functional studies in multiple model organisms^5^. However, Porifera and Ctenophora lack Hox homologues and still exhibit body polarity either in the larval stage (Porifera) or throughout their life cycle^6,7^. In the case of Cnidaria, Hox-like genes have been identified in multiple organisms and only one bilaterian homologue of anterior *Hox1* was found in each organism. In contrast, other Hox-like genes exhibit weak similarity with known bilaterian central/posterior Hox genes^8–10^. Their expression pattern is also not colinear as observed in Bilateria suggesting an alternative mechanism in body axis patterning. Remainder of the conserved homeobox genes might play a critical role in this process along with other signalling pathways^9,11^.

The Wnt signalling pathway has been shown to play a conserved role in determining the polarity of primary body axis^12^. In many of the sponges polarized expression of *Wnt* genes has been reported and ectopic activation of Wnt signalling resulted in induction of multiple oscula^13–15^. Similarly, in ctenophores, Wnt transcripts are expressed at one side (aboral side) of the embryo during development^16^. In *Hydra*, canonical Wnt signalling is involved in organization of the head (oral region) and thus axis patterning^17^. It has been reported that expansion of Wnt ligands took place before the Cnidaria–Bilateria split resulting in 11 Wnts in *Hydra*. Six of these namely *HyWnt1*, *-7*, *-9/10a*, *-9/10c*, -*11* and -*16* have been shown to be expressed in the organizer region^18^. Further study revealed that the Wnt/β-catenin signalling pathway regulates tissue morphogenesis along with the non-canonical Wnt signalling pathway^19^. In a recent study, six Wnt signalling target genes were identified which are regulated during regeneration of both head and foot. In adult polyps four of these genes namely *zebra, matrixin, cathepsinL1-like* and *hv100202099* were shown to be expressed in regions other than the organizer. Additionally, binding of hyTcf on these genes has been demonstrated in whole polyps which does not corroborate with hyTcf and target gene expression domains^20^. This suggests that their role is related more with the regeneration rather than the organizer function. Recent studies identified thrombospondin and Sp5 as negative feedback regulators in *Hydra*^21,22^. Though previous studies shed light on the role of Wnt/β-catenin signalling in *Hydra* head organizer function and identified a couple of downstream targets, however, comprehensive knowledge regarding the downstream molecular players is lacking.

To gain insight into the molecular players involved in this mechanism, a transcriptome analysis was carried out under systemic Wnt activation and siRNA-mediated *β-catenin* knockdown conditions. An in-house transcriptome assembly and annotation with better coverage facilitated a comprehensive RNA-Seq analysis. Here, we identified genes that are regulated by Wnt signalling in a β-catenin-dependent manner. We have also demonstrated the conserved role of selected transcription factors in early embryonic axis patterning in vertebrates using the zebrafish model system. Further comparison of molecular repertoire regulated by Wnt/β-catenin signalling in *Hydra* with planaria and *Xenopus* indicates an unusually higher similarity with the latter. Our results provide insights into the molecular signature and its regulation that determine the function of an ancestral organizer important in body axis patterning.

## Results

### Transcriptome assembly and regulation of global gene expression upon activation of Wnt signalling

**Fig. 1:**
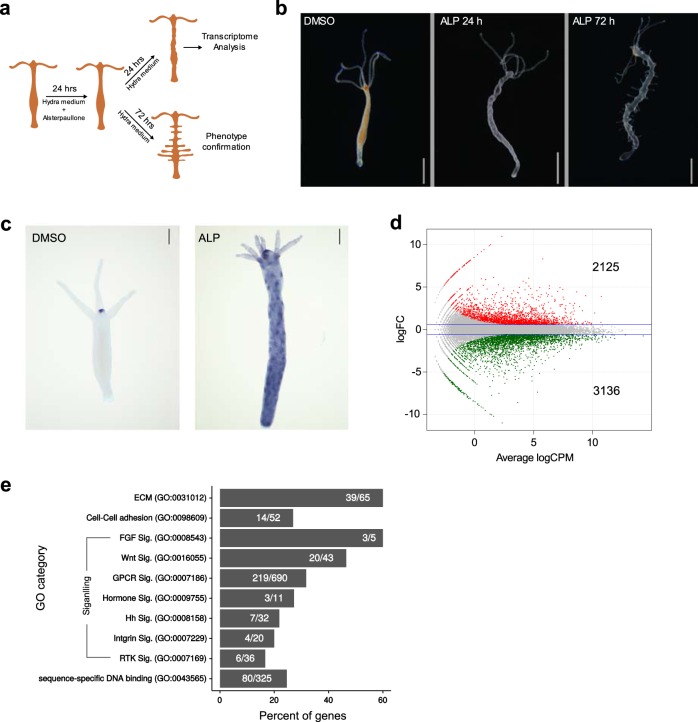
Global molecular changes that result in a drastic phenotypic alteration with expanded hypostome-like features upon systemic activation of Wnt signalling. Wnt-regulated genes were identified by differential gene expression (DE) profiling after inhibiting GSK-3β with Alsterpaullone (5 μM). **a** Cartoon depicting the experimental design for activation of Wnt signalling and collection of samples at different time points for transcriptome and phenotype analysis. **b** Morphological changes after 24 h and ectopic tentacle formation after 72 h of Alsterpaullone treatment (5 µM). Scale bar represents 1 mm. **c** Expression pattern of *HvWnt3a* in DMSO control and ALP-treated polyps 24 h post treatment. Scale bar represents 200 µm. **d** Smear plot showing logarithmic fold change of transcript expression compared to control on *y*-axis and log of read counts per million on *x*-axis. Red and Green colour dots denote significantly up- and downregulated genes with log_2_ fold change cut-off of ±0.58 with a *p* value of 0.01 respectively; two biological replicates for each condition were used for DE analysis. **e** Bar plot showing the percentage of genes differentially regulated in selected GO term classes, upon activation of Wnt signalling. Numbers in the bars represent genes that exhibit altered expression over total genes of respective GO categories.

In *Hydra*, canonical Wnt signalling is involved in the organization of the head (oral region) and therefore also in axis patterning^17,23^. Activation of Wnt signalling by inhibiting GSK-3β using Alsterpaullone (ALP) treatment results in the expression of head-specific genes and the formation of ectopic tentacles all over the body column^24^. Similar observations were made in another cnidarian model system *Nematostella*^25^. This indicates that the entire body column acquires hypostome-like characteristics upon activation of Wnt signalling and can be exploited to identify the molecular players involved in the process of axis formation since the entire signalling mechanism is amplified. Upon ALP treatment the morphological changes begin to appear 24 h post treatment as protuberances on the body column and after 72 h the ectopic tentacles appear (Fig. 1a, b). To determine the early molecular changes during morphogenesis after ALP treatment, we selected the 24 h time point for differential expression through transcriptome analysis (Fig. 1a, c).

The available *Hydra magnipapillata* genome assembly is incomplete and the reference gene models are in silico predicted^26^. To overcome this limitation a hybrid transcriptome assembly was built by merging the in-house de novo transcriptome assembly and the NCBI RefSeq (Supplementary Fig. 1; Supplementary Information). Comparison of this hybrid assembly with *H. magnipapillata* RefSeq revealed that our hybrid assembly exhibits 99.6% completeness compared to 95.7% of RefSeq (Supplementary Fig. 2a; Supplementary Information). Functional annotation of the hybrid assembly resulted in the annotation of maximum number of transcripts with highest possible coverage of the gene ontology (GO) terms (Supplementary Fig. 2b; Supplementary Information). Differential expression analysis of Wnt activated polyps using the custom hybrid transcriptome assembly demonstrated that 5261 transcripts are dysregulated (Fig. 1d, Supplementary Data 1). Functional classification based on the associated gene ontology (GO) terms revealed that multiple genes associated with the cell–cell adhesion, extracellular matrix (ECM) and cytoskeletal components were dysregulated (Fig. 1e). These gene products play critical roles in planar cell polarity and morphogenesis and are regulated by Wnt signalling in complex organisms^27^. Additionally, SHH (sonic hedgehog), RTK (receptor tyrosine kinase) and hormone-mediated signalling pathways important for organismal development are also regulated by Wnt signalling in *Hydra* (Fig. 1e). Previous reports suggest the origin and divergence of many bilaterian specifics RTKs^28^ along with other important developmental signalling pathway members in early divergent phyla and their conservation in *Hydra*^17,29^. This highly correlates with the transition of bilaterian-specific innovations such as evolution of different cell types, body patterning and morphogenesis. Collectively, these findings clearly indicate a crosstalk of the Wnt signalling pathway with diverse classes of molecules involved in the developmental processes and therefore suggest its central role in controlling the organization of the body plan.

### Transcription factors regulated by Wnt signalling in *Hydra*

**Fig. 2:**
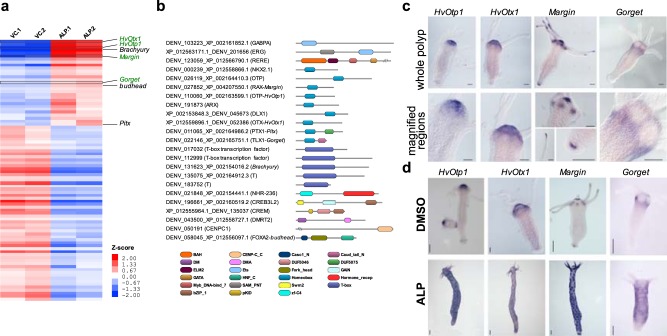
Homeobox family transcription factors (TFs) upregulated upon activation of Wnt signalling are expressed in head (oral side) of the polyp. Hierarchical clustering of differentially expressed TFs was carried out and domain organization of the upregulated TFs was studied. Expression analysis of selected TFs was carried out by in situ hybridization. **a** Heatmap of differentially expressed TFs after activation of Wnt signalling. VC.1, VC.2, biological replicates of vehicle (DMSO) controls; ALP.1, ALP.2, biological replicates of Alsterpaullone (5 μM)-treated samples; intensity of red and blue colours indicates respectively the higher and lower expression levels of genes compared to the controls; red and blue colours denote significantly up- and downregulated genes with log_2_ fold change cut-off of ±0.58 with a *p* value of 0.01, respectively; genes that have been previously reported and characterized in *Hydra* are labelled in black and genes investigated in the current study are labelled in green. **b** Domain organization of TFs upregulated by Wnt signalling. **c** Expression pattern of *HvOtp1*, *HvOtx1*, *Margin* and *Gorget* and magnified images of selected regions of the polyps displaying expression. Scale bar represents 100 µm. **d** Expression pattern of *HvOtp1*, *HvOtx1, Margin* and *Gorget* in DMSO controls and Alsterpaullone (5 µM)-treated polyps. Scale bar represents 200 µm.

Major regulatory switches are controlled by developmental signalling pathways which culminate in expression of transcription factors (TFs) during axis pattern formation^30^. Here, 88 unique TFs are differentially regulated by activation of Wnt signalling (Fig. 2a, Supplementary Data 2). Among these 29 are upregulated and 59 are downregulated. In the upregulated TFs, 9 belong to homeobox gene family members (Fig. 2b). Among the upregulated TFs, Brachyury^31^ and *Budhead*^32^ have been shown to play a role in *Hydra* oral fate determination based on their expression pattern and *Pitx* has been shown to be regulated by Wnt/β-catenin signalling^29^. The downregulated transcripts comprise of 16 Homeobox, 12 Forkhead and 10 bZIP members (Supplementary Fig. 3). Majority of the upregulated homeobox genes belong to the PRD class and we focused our analysis on these genes. Phylogenetic analysis resulted in clear classification of Otp and Otx homologues. However, two genes (*Gorget* and *Margin*) do not exhibit clear phylogenetic affinities to known bilaterian homologues (Supplementary Fig. 4; Supplementary Information). To understand the role of these upregulated homeobox genes, we monitored the localization of expression of selected TFs by in situ hybridization.

The expression of *HvOtp1* is observed at the tip of hypostome predominantly in endoderm and sparsely in the ectoderm surrounding the mouth (Fig. 2c). Additionally, a recent study of the expression of transcripts at single-cell resolution confirms the observed expression pattern specific to both ectodermal and endodermal epithelial cells in the head. A higher expression in spumous and granular mucosal gland cells and a lower expression in a neuronal sub-population is also evident from the plots generated from the single-cell RNA-seq data^33^ (Supplementary Fig. 5a). In *Nematostella*, the *HvOtp1* counterpart *NvOtp* has been shown to be expressed at the oral end, beginning in the mid planula stage and also in specific neuronal cell types^34^. *HvOtx1* expression is observed predominantly in the ectodermal epithelial cells and relatively less in the endodermal epithelial cells of the hypostome region. Furthermore, comparison with the single-cell RNA-seq data from Siebert et al.^33^ strongly corroborates the ectodermal expression pertinent to the head region (Supplementary Fig. 5b). Three *otx* genes were found as a cluster in *Nematostella* genome and were speculated to play a role in the anterior neural development and endomesoderm formation similar to bilaterians^35^. In bilaterian model organisms *otp* has been shown to play a critical role in the differentiation of neuronal cell types and thus development of parts of the central nervous system (CNS)^36,37^. This suggests that *HvOtp1* plays a role in neuronal differentiation at the oral end of the polyp. Bilaterian *otx* genes are pivotal in the development of the brain and the eye^38^. Expression analysis of *otp* and *otx* homologues in *Saccoglossus kowalevskii* in the prosome and the mesosome/anterior metasome epithelial cells respectively and not in the neurons suggests their primary role could be in patterning the anterior and central parts of the brain ectoderm^39^. Though comparable structures such as brain are not observed in *Hydra*, the expression pattern suggests that *HvOtp1* and *HvOtx1* might be involved in the fate determination of hypostome epithelial cell types and additionally *HvOtp1* in some neuronal populations. The expression pattern of *HvOtp1* and *HvOtx1* associated with the organizer region is also evident as distinct spots upon activation of Wnt signalling, similar to *HvWnt3a* (Fig. 2d, Supplementary Fig. 6).

The paired homeobox gene, *Margin*, without a clear homology to any known bilaterian member of the class (Supplementary Fig. 4) exhibits four major domains of expression namely, the hypostome, base of the tentacles, tip of the tentacles and the basal disc. This signature expression pattern at the margins of morphological features prompted us to name it as *Margin*. The expression is localized to endoderm except at the tip of the hypostome (Fig. 2c). *Margin* displays spotted expression upon activation of Wnt signalling, similar to *HvOtp1* (Fig. 2d, Supplementary Fig. 7c). The expression is localized presumably to the regions where tissue folding occurs, suggesting a role in tissue morphogenesis. A Tlx-like homeobox TF is upregulated upon activation of Wnt signalling which has no clear homologue in the bilaterian phyla. In situ expression of this transcript is restricted to the zone around base of the tentacles excluding hypostome (Fig. 2c). We named this gene *Gorget* based on its expression pattern that resembles a gorget, the piece of a soldier’s armour used to protect region around the neck and collar. Activation of Wnt signalling led to the ectopic expression of *Gorget* in circular patches all over the body column with a clear zone of exclusion in the centre of each patch that possibly demarcates the region from which a new tentacle will emerge (Fig. 2d, Supplementary Fig. 7c). The expression pattern of *Gorget* around the tentacle and presumptive tentacle formation zones observed in the ectopic Wnt activated condition suggests its possible role in tentacle formation. The expression patterns of both *Margin* and *Gorget* are also corroborated by a recent study on stem cell differentiation trajectories using single-cell RNA-seq^33^ (Supplementary Fig. 5c, d).

### β-catenin mediated regulation of TFs by Wnt signalling

**Fig. 3:**
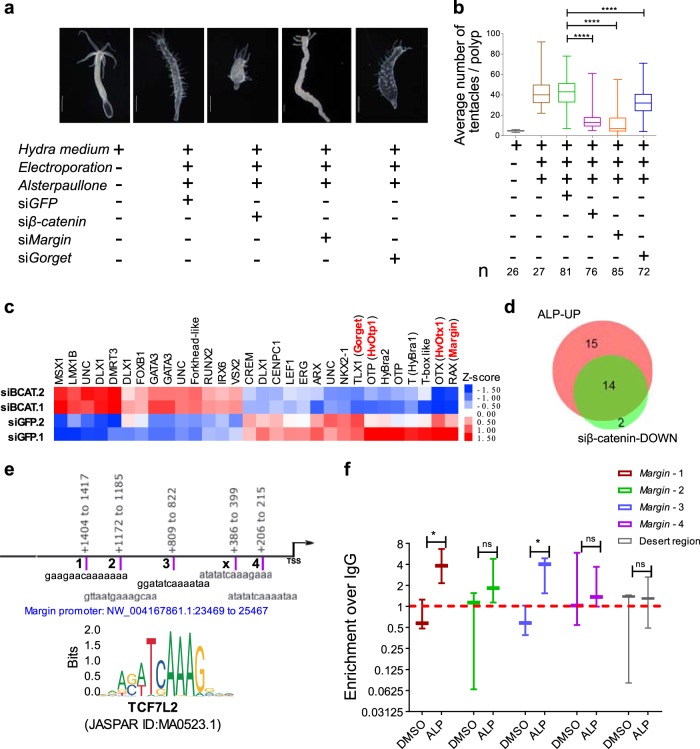
Inhibition of ectopic tentacle phenotype by knockdown of *β-catenin* and identification of Wnt/β-catenin target genes. To identify the *β-catenin-* mediated gene regulation by Wnt signalling, transcriptome analysis was performed following ectopic activation of Wnt signalling and knockdown of *β-catenin*. **a**, **b** Inhibition of the ectopic tentacle phenotype caused by systemic activation of Wnt signalling at 62%, 72% and 25% upon knockdown of *β-catenin*, *Margin* and *Gorget*, respectively compared to siGFP controls. Scale bar in **a**, 500 µm; *N* = 3; *n* = x in **b**, number of polyps; ***p < 0.001. **c** Heatmap of significantly differentially regulated TFs by Wnt signalling in β-catenin-dependent manner. siGFP.1 and siGFP.2, are the biological replicates of the controls where siGFP was used; siBCAT.1 and siBCAT.2 are the biological replicates of *β-catenin* knockdown samples; intensity of red and blue colours indicates respectively the higher and lower expression of genes compared to the controls; significantly dysregulated genes were selected based on log_2_ fold change cut-off of ±0.58 with a *p* value of 0.05; TFs highlighted in red colour are investigated in this study; UNC, uncharacterized genes with no clear homology to known genes. **d** Venn diagram depicts the overlap of TFs upregulated by activation of Wnt signalling and downregulated by *β-catenin* knockdown. ALP-UP, upregulated TFs upon Alsterpaullone treatment; siβ-catenin-DOWN, downregulated TFs after *β-catenin* knockdown. **e** TCF7L2-binding motif used to scan the *Margin* promoter (2 kb upstream to TSS). Binding motifs are highlighted in purple coloured bars where the numbers adjacent to these bars denote the motif containing region selected for ChIP qRT-PCR; TCF7L2-binding motif sequences are given below the bars and genomic locations relative to TSS are given above the bars. **f** ChIP qRT-PCR performed with different sets of primers designed to amplify the TCF7L2-binding motifs in *Margin* promoter after Alsterpaullone (5 µm) treatment and in DMSO solvent control. Plot displays log_2_ fold enrichment of *β-catenin* occupancy over IgG control; Desert denotes the region with no variable occupancy under control and treatment conditions; *N* = 3; Students two-tailed *t*-test was performed; *p value < 0.1.

To identify the genes that are regulated by Wnt signalling in a β-catenin-dependent manner we activated Wnt signalling after siRNA mediated knockdown of *β-catenin* and performed differential expression analysis (Supplementary Fig. 7). Additionally, we performed knockdown of *Margin* and *Gorget* (Supplementary Fig. 7). Lack of ectopic tentacle phenotype confirmed an effective functional knockdown of *β-catenin* mediated Wnt signalling (Fig. 3a, b, Supplementary Fig. 8). We also observed reduction in the body axis confirming the critical role of Wnt/β-catenin signalling in body axis patterning (Supplementary Fig. 8). *Margin* knockdown resulted in the complete absence of ectopic tentacles whereas *Gorget* knockdown yielded a partial reduction (Fig. 3a, b, Supplementary Fig. 8). Differential gene expression analysis preceding morphological changes exhibited a total of 989 transcripts that are differentially regulated, out of which 459 are upregulated and 530 are downregulated (Supplementary Fig. 9a, Supplementary Data 3). From these we identified 30 putative TFs to be dysregulated (Fig. 3c, Supplementary Data 4). Comparative analysis of the TFs that are upregulated upon activation of the Wnt signalling and downregulated under *β-catenin* knockdown condition and vice versa was performed to decipher the direct Wnt/β-catenin targets. Such analysis revealed that 14 of the 29 upregulated (Fig. 3d) and 11 of the 59 downregulated TFs (Supplementary Fig. 9b) were regulated by β-catenin-dependent Wnt signalling. *Margin* and *Gorget* are also downregulated upon *β-catenin* knockdown suggesting their regulation by Wnt/β-catenin signalling. Next, to monitor the binding of β-catenin to these targets we performed chromatin immunoprecipitation (ChIP) using anti-β-catenin antibody followed by a ChIP-qPCR analysis of the predicted binding sites of TCF7L2 at the *Margin* promoter. We demonstrate that *β-catenin* binds to three out of four putative TCF7L2-binding sites on the *Margin* promoter. This binding is further enhanced on two sites upon activation of Wnt signalling suggesting direct regulation of *Margin* expression by *β-catenin* (Fig. 3e). Furthermore, the occurrence of TCF7L2-binding motifs on the promoters of *HvOtp1, HvOtx1* and *Gorget* indicates possible regulation by *β-catenin* (Supplementary Information). Analysis of expression of the 16 TFs identified as putative targets of Wnt/β-catenin signalling from a published study on transcriptome analysis of regenerating tips of *Hydra* polyps^22,40^ revealed that most of these TFs exhibit elevated expression during head regeneration (Supplementary Fig. 10). These results strongly argue in favour of the role of these candidate TFs in organizer formation and axis patterning during regeneration.

Another important class of TFs that play a critical role in axis patterning are Hox components. In the present study, upon systemic activation of Wnt signalling, downregulation of *CnoxB_HVUL* is observed and a previous study has shown ectopic expression of *Hox1_HVUL* gene by in situ hybridization^8^, under similar experimental conditions. However, we did not find any change in the expression of these genes following the knockdown of *β-catenin*. Additionally, studies in another cnidarian model organism *Nematostella* have shown the downregulation of *Nv-anthox1* (homologous to *CnoxB_HVUL*)^25^. Based on these observations we propose that the anterior Hox gene in *Hydra* and aboral genes in both *Hydra* and *Nematostella* are regulated by the non-canonical Wnt signalling. Collectively, these results indicate that Wnt regulates the anterior Hox gene in Cnidaria suggesting an earlier crosstalk of the Wnt and the Hox components. Here, it should be noted that the aboral *Nv-anthox1* and its *Hydra* homologue (*CnoxB_HVUL*) which are downregulated probably by the non-canonical Wnt signalling exhibit weak phylogenetic affinities with the central/posterior Hox genes^8,10^. Therefore, it is difficult to extend the observations made in Cnidaria to bilaterians pertaining to the regulation of posterior Hox genes by Wnt signalling. A recent report has shown that the *Hydra* SP5 homologue (*HySp5*—accession number: MG437301) is a target of Wnt/β-catenin signalling^22^. However, in the transcriptome analysis we did not observe any expression level changes in *HySp5* upon systemic Wnt activation condition or upon knockdown of *β-catenin* under the same condition. The corresponding gene ID for the same in our study is XM_004206770.2_DENV_188204.

### Wnt/β-catenin signalling regulates genes associated with dorsoventral axis patterning

**Fig. 4:**
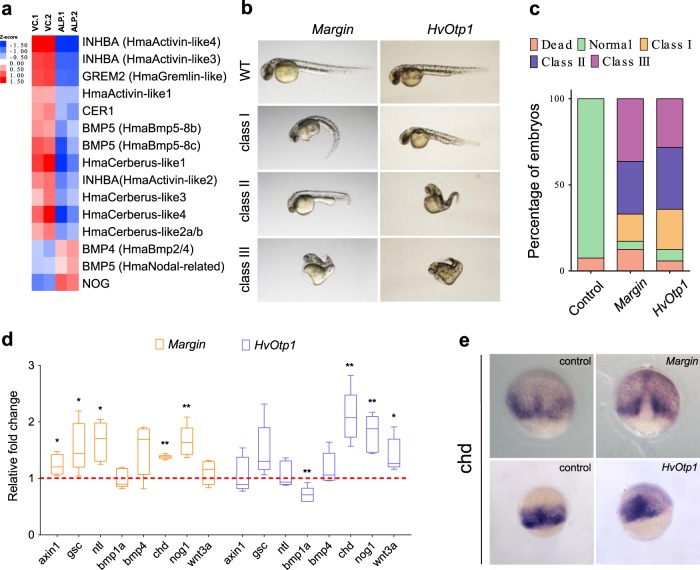
*Hydra* Wnt signalling targets regulate genes that play a crucial role in dorsoventral patterning. Upregulation of Wnt signalling results in negative regulation of bilaterian-specific ventral markers. To test the role of Wnt/β-catenin targets in dorsoventral patterning we have used a heterologous model system, zebrafish. *Hydra Margin* and *HvOtp1* genes were selected and in vitro synthesized capped mRNAs were injected into one-cell stage zebrafish embryos. **a** Heatmap displays the significantly differential regulated TGF-β family members upon activation of Wnt signalling. Red and blue colours denote significantly up- and down- regulated genes with log_2_ fold change cut-off of ±0.58 with a *p* value of 0.01 respectively. **b** Morphological changes upon injections of *Margin* and *HvOtp1* mRNA. Embryos were imaged at 30 hpf (lateral view, anterior to the left) and further classified based on the extent of phenotypic changes into three sub-classes. **c** Percentage of total embryos observed with different classes of phenotypic changes (*N* = 3). **d** Expression levels of axis determining genes upon injections of *Hydra Margin* and *HvOtp1* mRNAs are represented as fold change with respect to antisense mRNA-injected control embryos at 75% epiboly stage. *N* = 3; Students two-tailed *t*-test was performed; *p value < 0.03; **p value < 0.002. **e** Whole-mount in situ hybridization analysis of dorsal organizer gene *chordin* (dorsal view).

We observed a *β-catenin-*mediated positive regulation of the Wnt signalling components (Wnt ligands, Axin1 and LEF1) and the Notch signalling components (Supplementary Data 3). This infers the existence of a Wnt-positive autoregulatory loop as suggested by Nakamura et al.^23^. However, to our surprise most of the Frizzled receptors are negatively regulated and this could be due an existing negative feedback mechanism which requires further investigation. The role of canonical Wnt signalling has been shown during dorsalization in the bilaterians^43^. Members of the TGF-β signalling have been shown to play a critical role during patterning of the ventral axis in vertebrates^44^. We also observed that most of the members of the TGF-β signalling pathway are negatively regulated by Wnt signalling, whereas its antagonist Noggin was positively regulated (Fig. 4a, Supplementary Data 3). This suggests that the conserved BMP-Noggin antagonism^41,42^ is regulated by Wnt signalling in a β-catenin-dependent manner (Supplementary Data 3).

To test whether *Margin* and *HvOtp1* participate in Wnt/β-catenin- mediated axis patterning in the vertebrate model systems, we separately injected *Margin* and *HvOtp1* mRNA in zebrafish embryos at the one-cell stage. We observe severe morphological changes at 30 hpf in both test systems (Fig. 4b). Interestingly, *Margin* injected embryos exhibit mild heart oedema and curled tail (class I), shortened anterior–posterior axis (class II) and severe heart oedema, dorsalized embryos and curled tail (class III) phenotypes. The *HvOtp1*-injected embryos exhibit shortening of body axis (class1) and severe reduction in the AP axis, dorsalization with curled body (class II and class III) (Fig. 4c). To understand the molecular mechanism(s) underlying these drastic phenotypic changes we analysed the expression of the dorsoventral axis determinants at the mid-gastrula stage (75% epiboly) after injection of *Margin* and *HvOtp1* mRNA (Fig. 4d). Notably, genes which are responsible for the specification of the dorsal organizer such as chordin (chd) and noggin1 (nog1) are upregulated upon the ectopic expression of both *Margin* and *HvOtp1*. Ectopic expression of *Margin* also resulted in the upregulation of *axin1*, *goosecoid (gsc)* and *no tail (ntl/ta)* whereas overexpression of *HvOtp1* results in the increased expression of *wnt3a* and decreased expression of the *chd* antagonist *bmp1a*. Further, whole-mount in situ hybridization (WISH) analysis revealed a mis-localized expression pattern of chd upon overexpression of both *Margin* and *HvOtp1* (Fig. 4e, dorsal view). Though the homologue of *Margin* is absent in zebrafish, the dorsalization observed upon its overexpression indicates its primary role in axis patterning. Similarly, *HvOtp1* also leads to dorsalization upon overexpression suggesting its conserved role in Wnt-dependent dorsoventral patterning.

### Crosstalk between dorsoventral patterning genes and Wnt/β-catenin signalling in *Hydra*

**Fig. 5:**
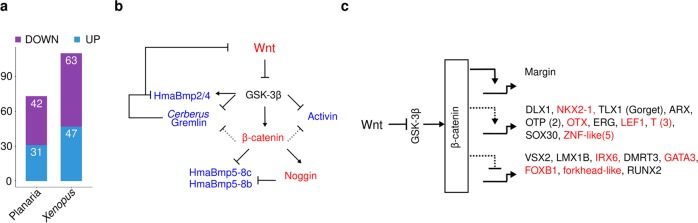
Conserved and novel homologues of bilaterian genes involved in axis patterning and regulated by the Wnt/β-catenin signalling pathway in *Hydra*. **a** Stacked bar plot depicts shared homologues of planaria and *Xenopus* that are differentially regulated by the Wnt/β-catenin signalling pathway in *Hydra*. **b** Predicted gene regulatory network of dorsoventral patterning genes based on differentially expressed gene under wnt activation condition. Genes highlighted in red colour are upregulated and genes highlighted in blue are downregulated; dotted line, β-catenin-dependent or -independent regulation. **c** Transcription factor targets of Wnt/β-catenin signalling identified in *Hydra*. TFs highlighted in red are also observed as part of the Spemann’s organizer signature^46^.

To identify the conserved molecular players involved in the organizer function and their evolution we compared the Wnt/β-catenin signalling targets identified in our study with that of planaria^45^ and *Xenopus*^46^. We found a conserved theme in functional categories of targeted genes by Wnt/β-catenin signalling in all the three systems (Supplementary Data 5). An important finding of this study is that the Wnt signalling pathway regulates molecules associated with cell–cell adhesion, extracellular matrix and cytoskeleton which are conserved and play a critical role in planar cell polarity and morphogenesis (Supplementary Data 5). Additionally, many of the TFs regulated by this pathway modulate cellular functions such as proliferation and differentiation in a context-dependent manner (Supplementary Data 5). A detailed comparison of the TFs was carried out to gain insight into their evolutionary adaption into the Wnt/β-catenin signalling pathway. Surprisingly, *Hydra* shares a higher extent of similarity in the genes regulated by Wnt/β-catenin signalling with the vertebrate organizer signature (*Xenopus*) than the phylogenetically closely related bilaterian (planaria) (Fig. 5a, Supplementary Data 5, Supplementary Information). Among these, the regulation of dorsoventral patterning components suggests a clear evolution of a regulatory crosstalk between the Wnt and BMP signalling pathways (Fig. 5b). The most striking conservation in the Wnt/β-catenin signalling TF targets is observed in the homologues of *Brachyury* (a T-Tbox member), *Otx* (a member of the PRD class of homeobox) and *Nkx* members (a sub-class of ANTP homeobox gene) apart from the well-characterized effector LEF1 (Fig. 5c). The current study also resulted in the identification of additional TFs such as the homologues/closely related members of ANTP class (DLX and TLX [*Gorget*]), PRD class (Arx, Otp and *Margin*) and SOX-like (Fig. 5c), suggesting their possible role in organizer functions or associated pattern formation in complex organisms.

### Wnt signalling determines posterior or anterior polarity?

There is an unresolved controversy over the similarity of cnidarian oral–aboral axis to the anterior–posterior axis of bilaterians^47^. The prevailing hypothesis is that the oral side is equivalent to the bilaterian posterior side^48^. This is primarily based on the bilaterian posterior polarization signals such as Wnt signalling^17^, localized expression of *Brachyury*^31^ to the oral side in Cnidaria and non-expression of head-specific markers such as *Otx*^49^ and *Nkx*^50^ in the oral domain. However, recent studies in *Nematostella* have shown that the oral side shares similarity with the anterior side based on the expression pattern of a conserved anterior Hox homologue at the oral pole and its regulation by Wnt signalling^25,51,52^. Further, it also exhibits gastrulation and formation of blastopore (future oral side) from animal pole unlike bilaterians^53,54^. Observations from this study and a previous report on gastrulation in *Hydra*^55^ are in concurrence with studies from *Nematostella*. The master regulatory TFs such as *Otx, Otp, Arx* and *Nkx* are involved in head formation in bilaterians^56^. The expression patterns of the homologues of these genes as revealed by our study suggest that the oral pole of *Hydra* and the bilaterian head share a similar signature of TF expression. This is also supported by the observations made in the first report of the role of Wnt signalling in the head organizer activity in *Hydra*, wherein it was demonstrated that *Hydra* β-catenin can induce a secondary axis and most anterior structures in *Xenopus*^17^. However, the genes that are downregulated by activation of Wnt signalling and upregulated in *β-catenin* knockdown condition did not include the TFs associated with posterior patterning. Therefore, we still need to understand the role of bilaterian posterior gene expression at the oral pole of *Hydra*. This can be resolved by studying the primary function of these molecules without prejudice based solely on the observations made in bilateria and focus on how they achieved bilaterian-specific functions. Based on conservation of the Wnt-BMP antagonism observed in our work we propose that there are strong similarities between the oral–aboral axis of *Hydra* and the dorsoventral axis of vertebrates. This observation also corroborates one of the perspectives expressed in the earliest report of the Wnt signalling in *Hydra*^17^. Additionally, expression of homologues of the bilaterian anteroposterior markers at the oral pole of *Hydra* indicates that these molecular players might have been opted by different signalling networks specifying their roles in anteroposterior axis pattering in the bilaterian lineage which appears to be spatially unresolved in *Hydra* (Cnidaria).

In summary, we have identified the gene repertoire regulated by the activation of Wnt signalling that plays a critical role in multiple biological processes including morphogenesis, cell differentiation and planar cell polarity. We found that many of the TFs along with TGF-β and Notch signalling components are integrated into the Wnt/β-catenin regulatory network. Further, we have demonstrated direct binding and regulation of the homeodomain TF *Margin* by β-catenin. The results presented here delineate the molecular signature of the organizer in *Hydra*. Additionally, comparative analysis of *Hydra* Wnt/β-catenin targets with bilaterian organizers suggests an unprecedented close similarity with vertebrates. Thus, the studies reported here have led to the identification of the molecular players important for formation of a definitive body plan in early metazoans.

## Methods

### Hydra culture

A clonal culture of *Hydra vulgaris* Ind-Pune was maintained at 18 °C using standard protocols described previously^57^. Hydra polyps were fed with freshly hatched *Artemia nauplii* larvae daily and cleaned 6–8 h post feeding.

### Alsterpaullone treatment

Twenty-four hours starved *Hydra* polyps were taken and subjected to 5 µM Alsterpaullone (Sigma—A4847) in *Hydra* medium for 24 h. Control treatments with *Hydra* medium and DMSO as a vehicle control were also performed. Alsterpaullone was removed after the pulse and this time point is considered as 0 h post treatment. The polyps were incubated in *Hydra* medium for an additional 24 h after which point they were harvested for either RNA isolation or WISH.

### RNA sequencing

Total RNA was extracted using Trizol (Thermo Fischer Scientific) and poly(A) RNA was purified. A minimum of two biological replicates were processed for each experimental condition. RNA-seq libraries were prepared using the TruSeq RNA library kit (Illumina). Briefly, purified mRNA was fragmented for 4 min at 94 °C in the presence of divalent cations and reverse transcribed using Superscript III Reverse transcriptase with random hexamers. After second-strand cDNA was synthesized and cleaned up using Agencourt Ampure XP SPR1 beads (Beckman Coulter). Illumina adapters were ligated followed by the end repair. After performing the cleanup, the library was amplified by running 11 cycles of PCR. The library was quantified using Nanodrop and quality was determined using High Sensitivity Bioanalyzer Chip (Agilent) and sequenced on Illumina-GAIIx sequencing platform.

### Hybrid transcriptome assembly and annotation

After performing quality control using FASTQC^58^ first 10 and last 2 bases of were trimmed using FASTX-toolkit (Gordon, A. & Hannon, G. Fastx-toolkit. *FASTQ/A Short-reads Preprocessing Tools.* http://hannonlab. cshl. edu/fastx_toolkit **5** (2010), unpublished). These sequence files were used to build de novo transcriptome assembly. Here, transAbyss tool was used to build the transcriptome with different k-mers (35–88 with the interval of 3). Assemblies for different k-mers were filtered by ‘abyss-ta-filter’ function. Processed assemblies were merged and duplicated were removed by ‘abysss-rmdups-iterative’ function. Open reading frames were predicted using TransDecoder^59^ and the selected coding sequences were used from this de novo assembly. To make more comprehensive reference transcriptome assembly we have compared in-house de novo assembly with *H. magnipapillata* RefSeq (NCBI) by performing reciprocal BLAST. Longer matching sequences were selected from de novo or RefSeq along with unique sequences which did not yield any hit in comparison (Supplementary Data 6—*Hydra vulgaris* Ind-Pune Hybrid assembly_cds.fsa). Functional annotation of this hybrid assembly was carried out along with RefSeq (*H. magnipappilata*) using IntproScan5 and BLASTP to UniRef90 (Supplementary Data 7—*Hydra vulgaris* Ind-Pune Hybrid assembly Annotation.gaf). The extracted gene ontology terms were plotted for comparing the annotation coverage using WEGO^60^. An independent analysis was carried out to estimate the completeness of the hybrid assembly using BUSCO^61^. This hybrid assembly was used for differential gene expression analysis. Details of commands used are included in Supplementary Information.

### Differential expression analysis

Differential expression analysis was performed using the EdgeR^62^ method integrated in Trinity pipeline^63^. Here, raw reads from experimental conditions were aligned to hybrid transcriptome (in-house build) by the bowtie2 and RSEM method using ‘align_and_estimate_abudance.pl’ script. Read count matrix was generated using ‘abundance_estimate_to_matrix.pl’ script. This read count matrix was used in EdgeR by running ‘run_DE_analysis.pl’. Differentially expressed genes were selected using a log2 fold change cut-off of ±0.58 (~1.5 absolute fold change) and adjusted *p* value of 0.01 (for ALP-treated samples) and 0.05 (*β-catenin* knockdown samples). Normalized count tables were generated in R and used to plot heatmaps of differentially expressed genes or functional categories based subsets in Cluster3^64^. Domain architectures of differentially regulated protein sequences were assigned by hmmscan and classified by neighbour joining method using DoMosaics tool^65^.

### siRNA mediated knockdown

The siRNA oligo sequences for *Margin* and *Gorget* were predicted using the siDESIGN Center tool (https://dharmacon.horzondiscovery.com/design-center/). The siGFP and siβ-catenin sequences were used from previous reports^29,66^. Forty non-budding polyps were taken for each condition and incubated in water for 30 min. The polyps were then placed into an electroporation cuvette with a 4 mm gap (Bio-Rad), and water was aspirated. Two hundred microliters of sterilized 10 mM HEPES (pH 7.0) containing 2 µM siRNA was then added. After the animals relaxed in the cuvettes, two consequent pulses of 100 V, 750 Ω, and 75 µF were given using the Bio-Rad Gene Pulser II electroporation system. The animals were then immediately transferred to 10 ml of restoration medium (20% dissociation medium (6 mM CaCl_2_, 1.2 mM MgSO_4_, 3.6 mM KCl, 12.5 mM *N*-Tris-[hydroxymethyl]methyl-2 aminoethanesulphonic acid (TES), 6 mM sodium pyruvate, 6 mM sodium citrate, 6 mM glucose) +80% *Hydra* medium) and allowed to recover for 1 day. On the following day, the animals were transferred to *Hydra* medium. This process was repeated thrice on alternate days following feeding and cleaning of the animals. After the third pulse of siRNA electroporation, an ALP treatment was performed, and animals were harvested for RNA isolation by disrupting them in Trizol (Thermo Fischer Scientific). For observing the morphology the polyps were relaxed for 2 min in 2% urethane and fixed in 4% paraformaldehyde before mounting in 50% glycerol and imaged using a Leica M205 FA stereomicroscope.

### Annotation of downregulated genes upon knockdown of *β-catenin*

Putative protein sequences of candidate genes from *Xenopus*^46^, planaria^45^ and current study were extracted from the in-house dataset. These protein sequences were mapped to their eukaryotic orthologues using default parameters by eggNOG-mapper tool^67^.

### Quantitative RT-PCR

The RNA from polyps and zebrafish embryos was isolated using Trizol (Thermo Fischer Scientific) and cDNA was prepared using high capacity cDNA synthesis kit (Applied Biosystems, Foster City, CA). Quantitative RT-PCR was performed using the FastStart Sybr Green Master Mix (Roche) and real-time PCR cycler (ViiA 7; Applied Biosystems). Relative expression was calculated using the delta-delta Ct method. The mean expression of histone H4 was used as an endogenous control for normalization for *Hydra* and that of EF1α was used for zebrafish. One-way ANOVA method was used to test for statistical significance. All qRT-PCRs were carried out using a minimum of three biological replicates. Primers used are listed in Supplementary Data 8.

### Whole-mount in situ hybridization

Target genes were cloned into pCR-Blunt II-TOPO (Invitrogen)/pGEM-T Easy (Promega); primer details are given in Supplementary Data 8. Digoxigenin-labelled sense and antisense RNA probes were prepared by in vitro transcriptions from templates amplified from recombinant plasmids containing the target genes using PCR. WISH was performed on the polyps as previously described^32^ with the following modifications. The animals were relaxed for 2 min in 2% urethane. Treatment with proteinase-K was performed for 15 min and heat-inactivation of the endogenous alkaline phosphatases was done at 70 °C for 15 min in 1× SSC. Digoxigenin-labelled RNA probes at a concentration of 400 ng/ml was used for hybridization at 59 °C. The post-hybridization washes were performed using 1× SSC-hybridization solution gradients (prepared in hybridization solution). After staining with BM-purple AP substrate for 1 h at room temperature, the animals were mounted in 80% glycerol for imaging. Imaging was performed using a stereomicroscope (M165 FC; Leica). WISH analysis for zebrafish embryos was performed as previously described^68^.

### Zebrafish care and staging

Zebrafish were maintained using standard protocols^69^. Embryos from WT (TU) strain were collected in E3 embryo medium and grown at 28.5 °C. For phenotype analysis embryos were grown till 30 hpf and for qRT-PCR and ISH analysis embryos were harvested at 75% epiboly following standard procedures.

### *Margin* and *HvOtp1* mRNA injections into zebrafish embryos

*Margin* and *HvOtp1* gene templates were used for capped mRNA synthesis using mMessage mMachine SP6/T7 kits. For generating sense mRNA for *Margin*, plasmid DNA was linearized with *Sal*I and transcribed in vitro using T7 polymerase. For *Hydra HvOtp1* sense mRNA, plasmids were digested with *Xba*I and transcribed in vitro using SP6 polymerase. EGFP mRNA transcribed from pCS2 vector was used as injection control. Four hundred picograms of *Margin* and 300 pg of *HvOtp1* (in 1 nl) were injected in the zebrafish embryos at one-cell stage.

### Chromatin immunoprecipitation

One thousand *Hydra* Polyps were crosslinked with 1% formaldehyde, lysed and sonicated in sonication buffer (10 mM Tris-HCl pH 7.5, 200 mM NaCl, 1% SDS, 4% NP-40, 1 mM PMSF) to obtain an average chromatin size of 300 bp. Chromatin was pre-cleared using 10 µl of the Dynabeads™ M-280 sheep anti-mouse IgG (Invitrogen) slurry for 1 h at 4 °C with gentle inverting. Immunoprecipitations for *β-catenin* was carried out in 1 ml of ChIP buffer (20 mM Tris-HCl pH 8.0, 150 mM NaCl, 2 mM EDTA, 1% Triton-X 100) with 2 µg of anti-active-β-catenin antibody (cat # 05-665; Sigma-Aldrich). An equal amount of the appropriate IgG control (Normal mouse IgG) was also used (cat # 12-371; Sigma-Aldrich). Immunoprecipitations were carried out with inverting at 4 °C for 14–16 h. The samples were then incubated with 20 µl of the Dynabeads™ M-280 sheep anti-mouse IgG (Invitrogen) for 3 h at 4 °C with gentle inverting. ChIP samples were reverse-crosslinked and the DNA was purified using the phenol–chloroform–isoamyl based purification method. Input chromatin was obtained after preclearing, by de-crosslinking and purifying input DNA using phenol–chloroform–isoamyl alcohol extraction based purification method. Purified DNA was subjected to qRT-PCRs using target region specific primers listed in Supplementary Data 8. Motif search for TCF7L2 was carried out on regions 2 kb upstream to the transcription start sites (TSS) of *Margin, HvOtp1, HvOtx1* and *Gorget* using FIMO tool^70^. After screening multiple genomic regions, we found that the H4 coding genomic region gave us relatively less variable binding across the experimental condition. Hence, this region was used as a desert. To verify the specificity of the amplification the melt curve plots for the predicted promoter regions of *Margin* and the desert regions are included in the Supplementary Information.

### Statistics and reproducibility

Significantly differentially expressed genes were identified by EdgeR using R version 3.4.1, after performing RNA sequencing under different biological conditions. Here, samples from two biological replicates were used. All qRT-PCRs were carried out using a minimum of three biological replicates. The statistical analyses were conducted as mentioned in the figure legends using the GraphPad Prism version 7.0c software. Domain organization was plotted using DoMosaics tool and heatmaps of normalized gene expression were visualized using cluster 3.0.

### Reporting summary

Further information on research design is available in the Nature Research Reporting Summary linked to this article.

## Supplementary information

Supplementary information

Descriptions of Additional Supplementary Files

Supplementary Data 1

Supplementary Data 2

Supplementary Data 3

Supplementary Data 4

Supplementary Data 5

Supplementary Data 6

Supplementary Data 7

Supplementary Data 8

Supplementary Data 9

Reporting Summary

## Data Availability

All NGS data are publicly available at NCBI SRA at SRR7051325-SRR7051332 (PRJNA451264). Nucleotide sequences of transcription factors investigated in this study have been deposited at NCBI GenBank (MH940207-MH940211). Source data for Figs. 3b, 3f, 4c, 4d and Supplementary Fig. 7b can be found in Supplementary Data 9.

## Code availability

Custom codes/one liners that are used in this study are available in Supplementary information and also will be provided upon reasonable request.
