# Supplementary material for: Molecular signature of an ancient organizer regulated by Wnt/β-catenin signalling during primary body axis patterning in Hydra
Source: Commun Biol. 2019 Nov 26;2:434. doi: 10.1038/s42003-019-0680-3 (PMC6879750; doi:10.1038/s42003-019-0680-3)
Supplement: Supplementary file 1 — Supplementary information [file 42003_2019_680_MOESM1_ESM.pdf]

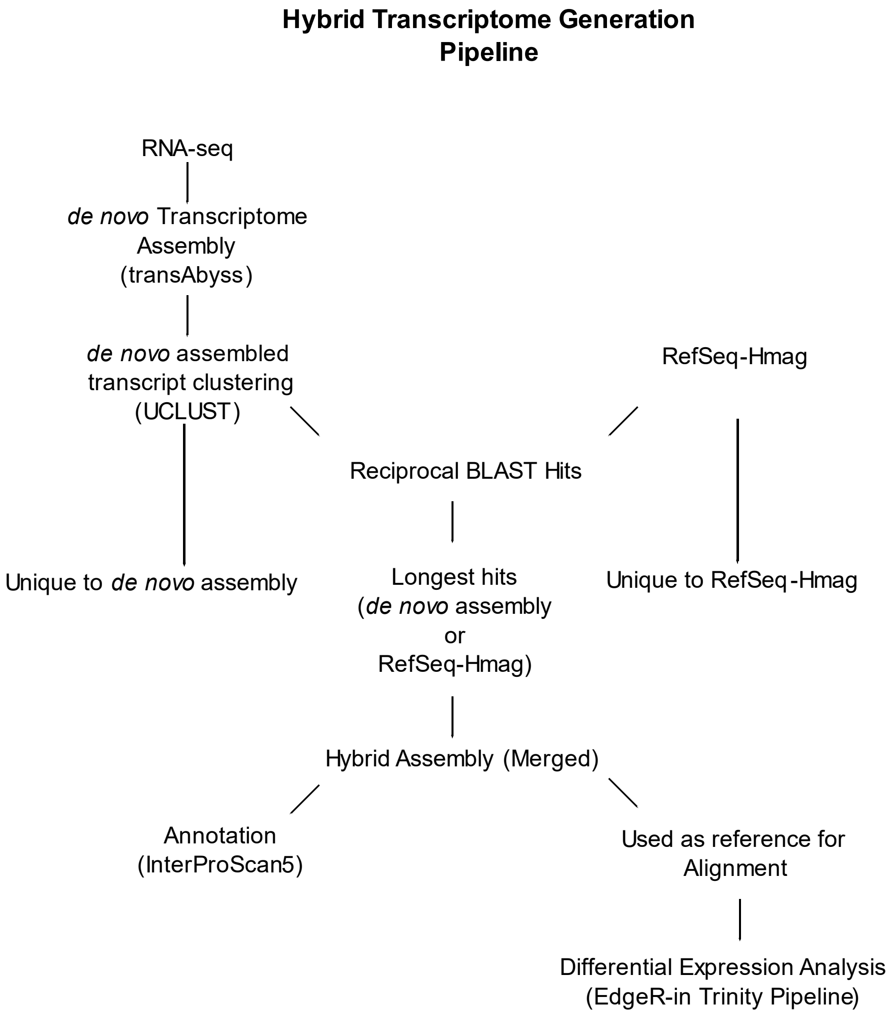

Supplementary Figure 1: Generation of a hybrid transcriptome assembly. After RNA sequencing raw reads from all experimental conditions were used to build the *de novo* assembly. A hybrid assembly was generated by merging missing sequences or longer transcripts from *Hydra magnipapillata* RefSeq.

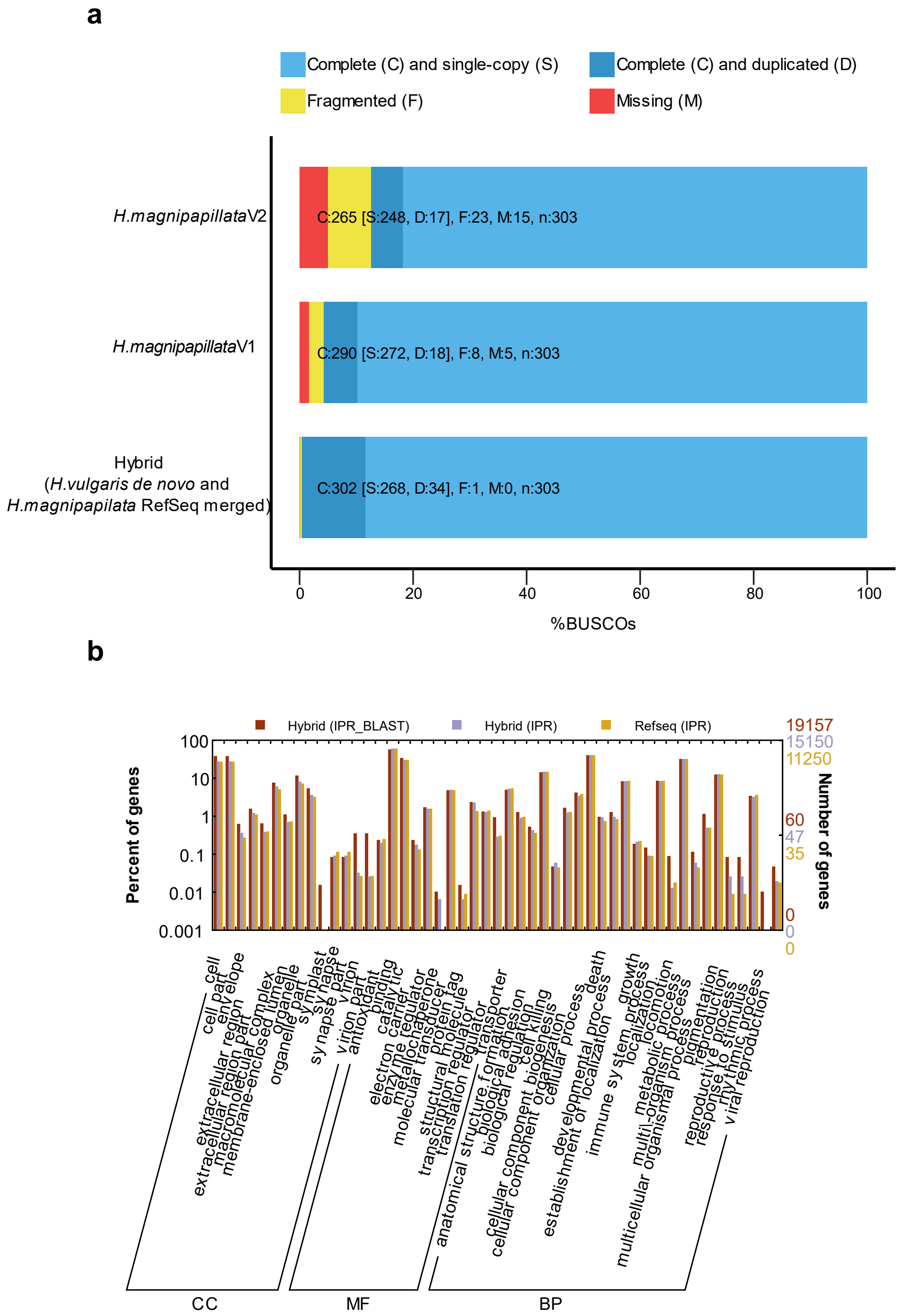

Supplementary Figure 2: Quality assessment and annotation of hybrid assembly. a, Evaluation of the quality of hybrid assembly and comparison with RefSeq data of *H. magnipapillata* and Hydra2.0 genome (*H. magnipapillata*). **b,** Comparison of different functional annotation methods on hybrid and RefSeq data. Barplot shows the coverage of gene ontology (GO) terms coverage. Hybrid assembly with InterProScan and BLAST based method gave best coverage.

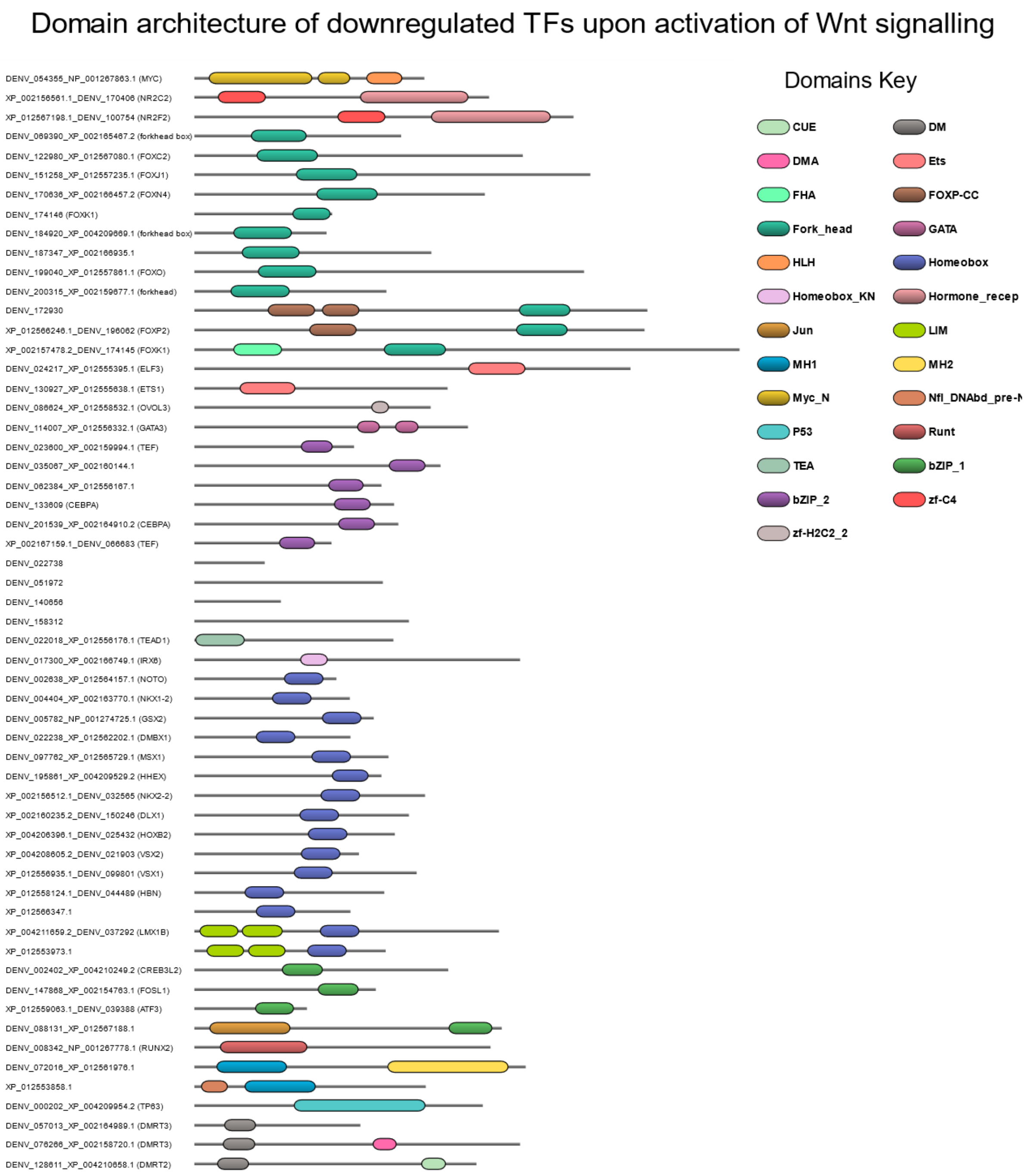

Supplementary Figure 3: Domain organization of downregulated TFs upon activation of Wnt signalling by inhibition of GSK3-β. Colour coded key labels indicate the corresponding Pfam domain.

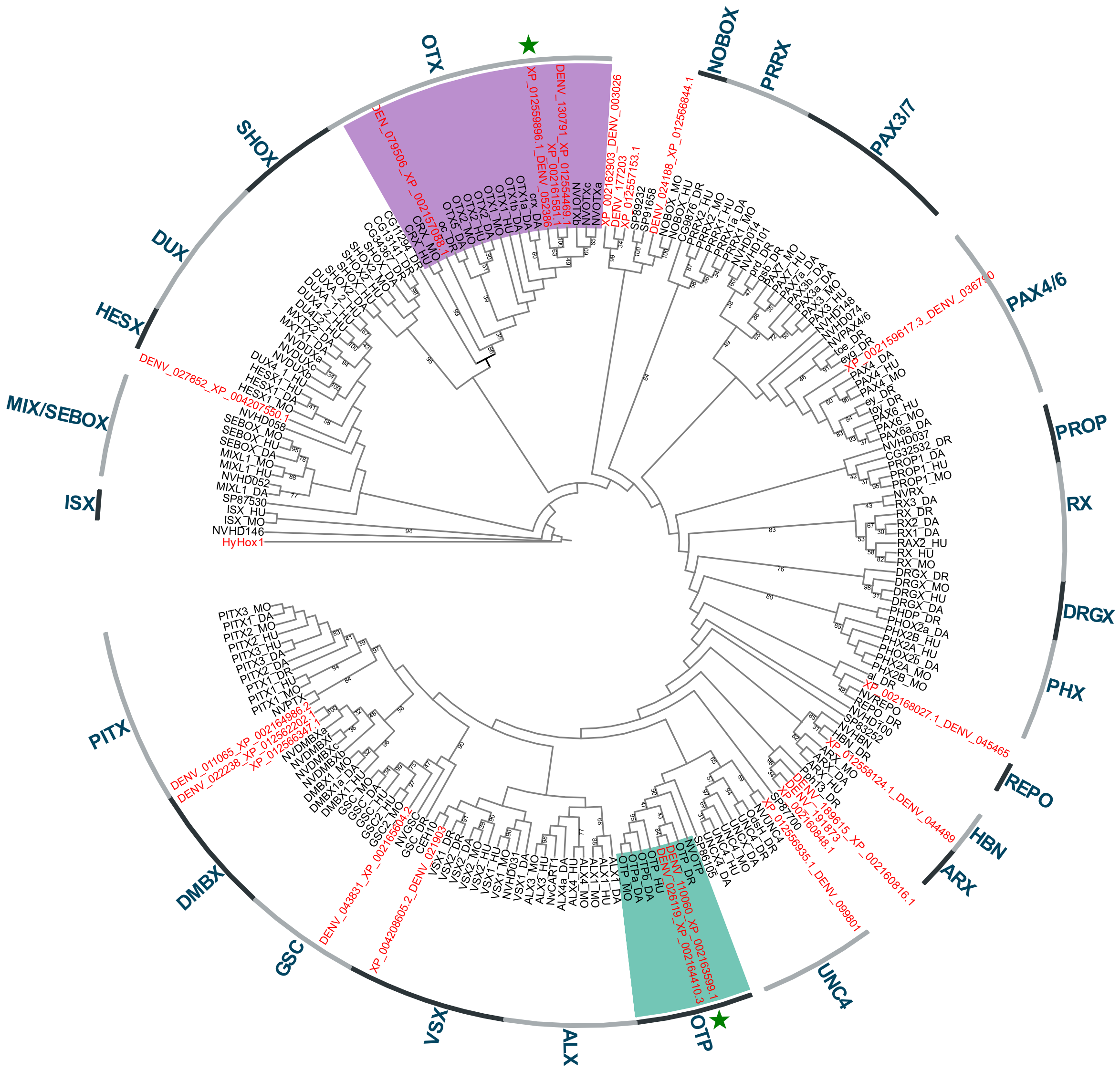

★ OTX and OTP homologues investigated in this study

Supplementary Figure 4: Phylogenetic analysis of PRD class of homeodomain proteins. Phylogenetic analysis of homeodomains from PRD members of selected animal phyla along with *Hydra* components was carried out by RAxML method. Here, the sequence alignments were performed using MUSCLE default parameters and after manually trimming they were used in analysis. Values on branches indicate the percentage confidence in branching point after 1000 bootstrap iterations. The sequence details are given in Supplementary Information. Sequences denoted with green star were used for further study in current work.

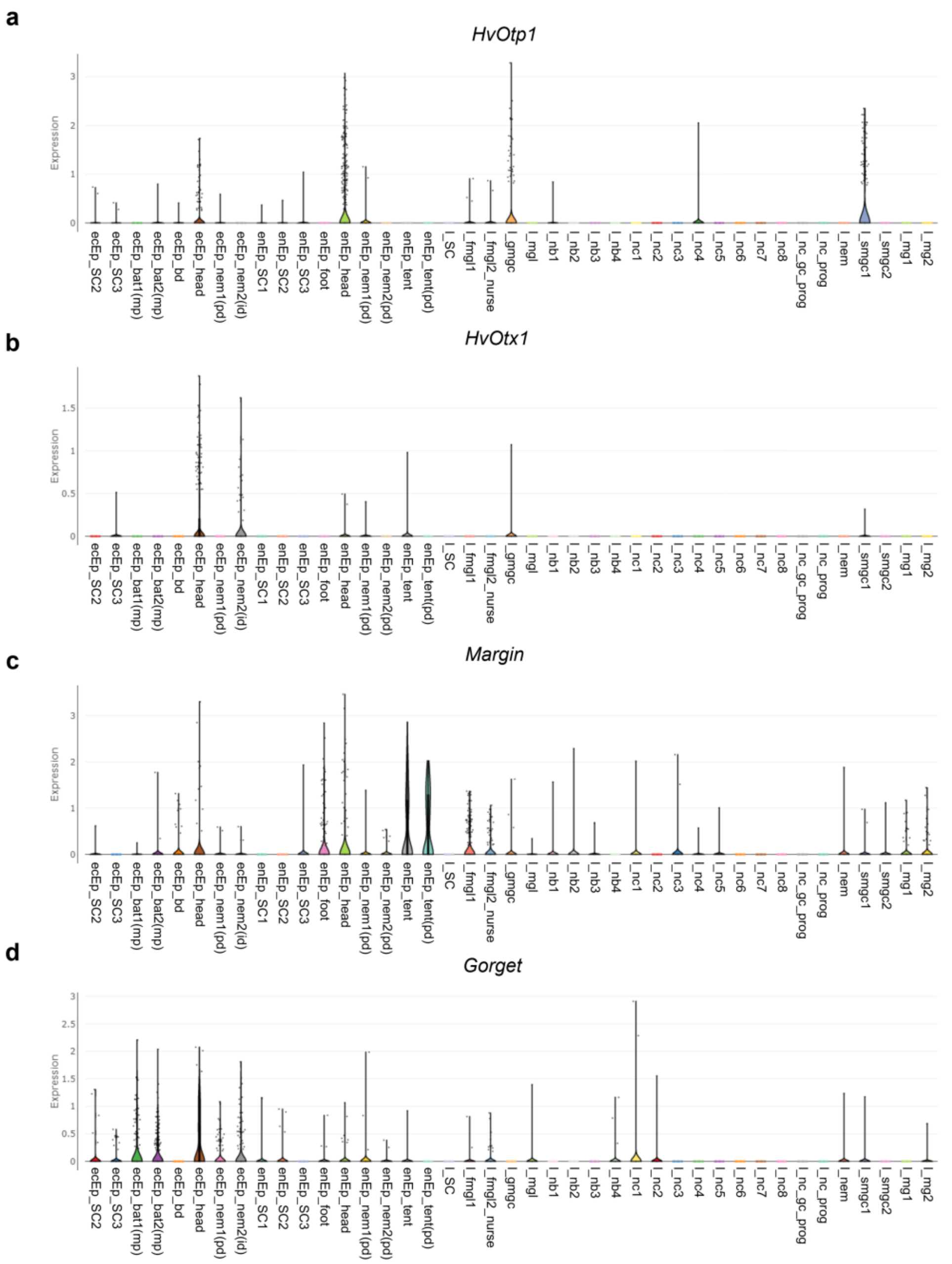

bat: battery cell, **bd**: basal disk, **db**: doublet cluster, **ecEP**: ectodermal epithelial cell, **enEP**: endodermal epithelial cell, **fmgl**: female germ-line, **gc**: gland cell, **gmgc**: granular mucous gland cell, **i**: cell of the interstitial lineage, **id**: integration double, **mgl**: male germline, **mp**: multiplet, **nb**: nematoblast, nc: neuronal cell, **nem**: nematocyte, **pd**: suspected phagocytosis doublet, **prog**: progenitor, **SC**: stem cell, **smgc**: spumous mucous gland cell, **tent**: tentacle, **zmg**: zymogen gland cell

Supplementary Figure 5: Expression of *HvOtp1*, *HvOtx1*, *Margin* and *Gorget* in different cell type cluster based on single cell transcriptome analysis. The expression plots were used from the data available online (https://portals.broadinstitute.org/single_cell/study/SCP260/stem-cell-differentiation-trajectories-in-hydra-resolved-at-single-cell-resolution). This data was generated by Siebert et. al., (2019) [1] for tracing the stem cell lineages in *Hydra* using single cell RNA-seq. **a**, Expression of *HvOtp1* in different cell-type clusters. **b**, Expression of *HvOtx1* in different cell-type clusters. **c**, Expression of *Margin* in different cell-type clusters. **d**, Expression of *Gorget* in different cell-type clusters. x-axis, names of the different cell-type clusters; y-axis, expression in the cells of different cell-type clusters.

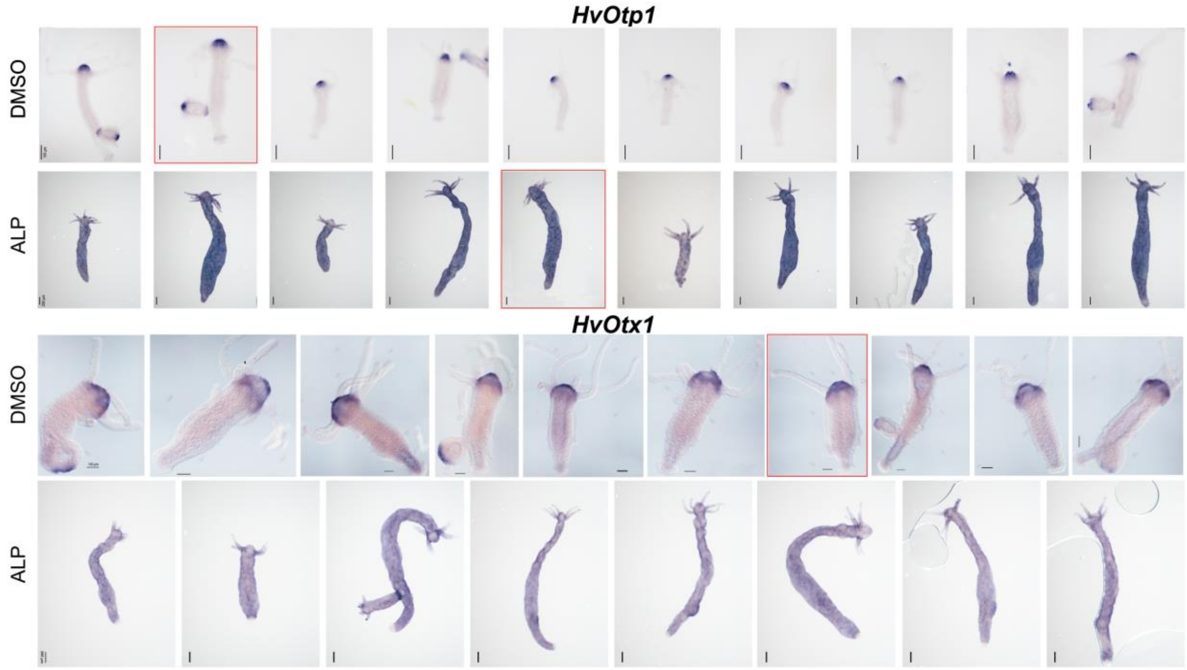

Supplementary Figure 6: Expression pattern of *HvOtp1* and *HvOtx1* upon activation of Wnt signalling. Family pictures of expression pattern of *HvOtp1* and *HvOtx1* by *in situ* hybridization after treatment with Alsterpaullone with respective controls. DMSO, vehicle control; ALP, 5 µM Alsterpaullone. The images with Red border line were used as representative images in Figure 2d.

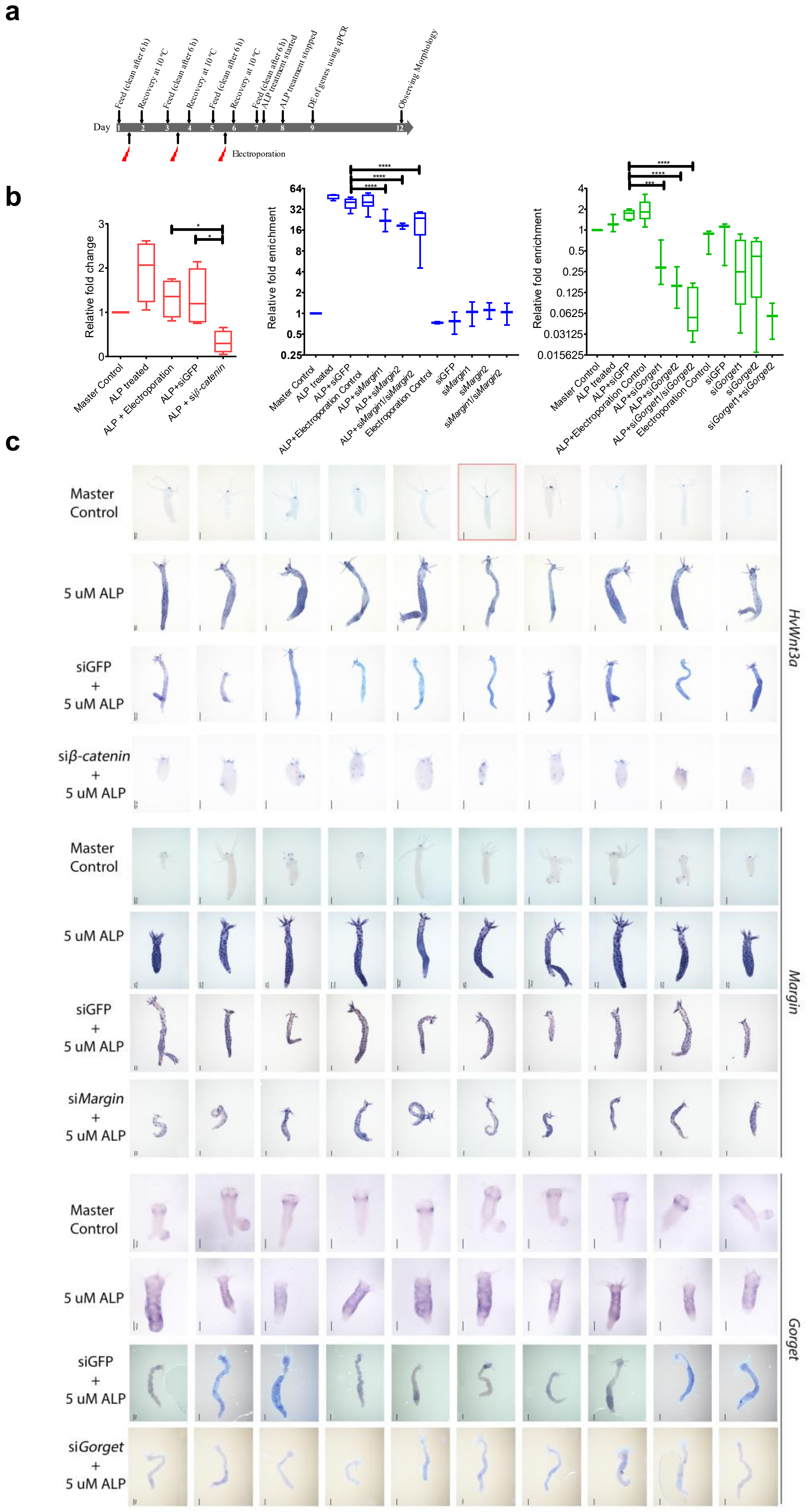
a
b
c
Day
Feed (clean after 6 h)
Recovery at 10°C
ALP treatment started
ALP treatment stopped
DE of genes using qPCR
Observing Morphology
Electroporation
Relative fold change
Relative fold enrichment
Master Control
ALP treated
ALP + Electroporation
ALP+siGFP
ALP + siβ-catenin
ALP+siGFP
ALP+Electroporation Control
ALP+siMargin1
ALP+siMargin2
ALP+siMargin1/siMargin2
Electroporation Control
siGFP
siMargin1
siMargin2
siMargin1/siMargin2
ALP+siGorget1
ALP+siGorget2
ALP+siGorget1/siGorget2
siGorget1
siGorget2
siGorget1+siGorget2
Master Control
5 uM ALP
siGFP + 5 uM ALP
siβ-catenin + 5 uM ALP
siMargin + 5 uM ALP
siGorget + 5 uM ALP
HvWnt3a
Margin
Gorget

Supplementary Figure 7: Knockdown of *β-catenin*, *Margin* and *Gorget*. Experimental design of siRNA mediated knockdown of *β-catenin*, *HvRx1* and *Gorget* followed by the activation of Wnt signalling. **b,** Validation of *β-catenin*, *HvRx1* and *Gorget* knockdown by qRT-PCR; ***, $p < 0.001$; **, $p < 0.01$; *, $p < 0.05$. **c**, Family pictures of expression scored by *in situ* hybridization upon knockdown of *β-catenin, Margin* and *Gorget* with controls. Image with red line border was used as the representative image in Figure 1c.

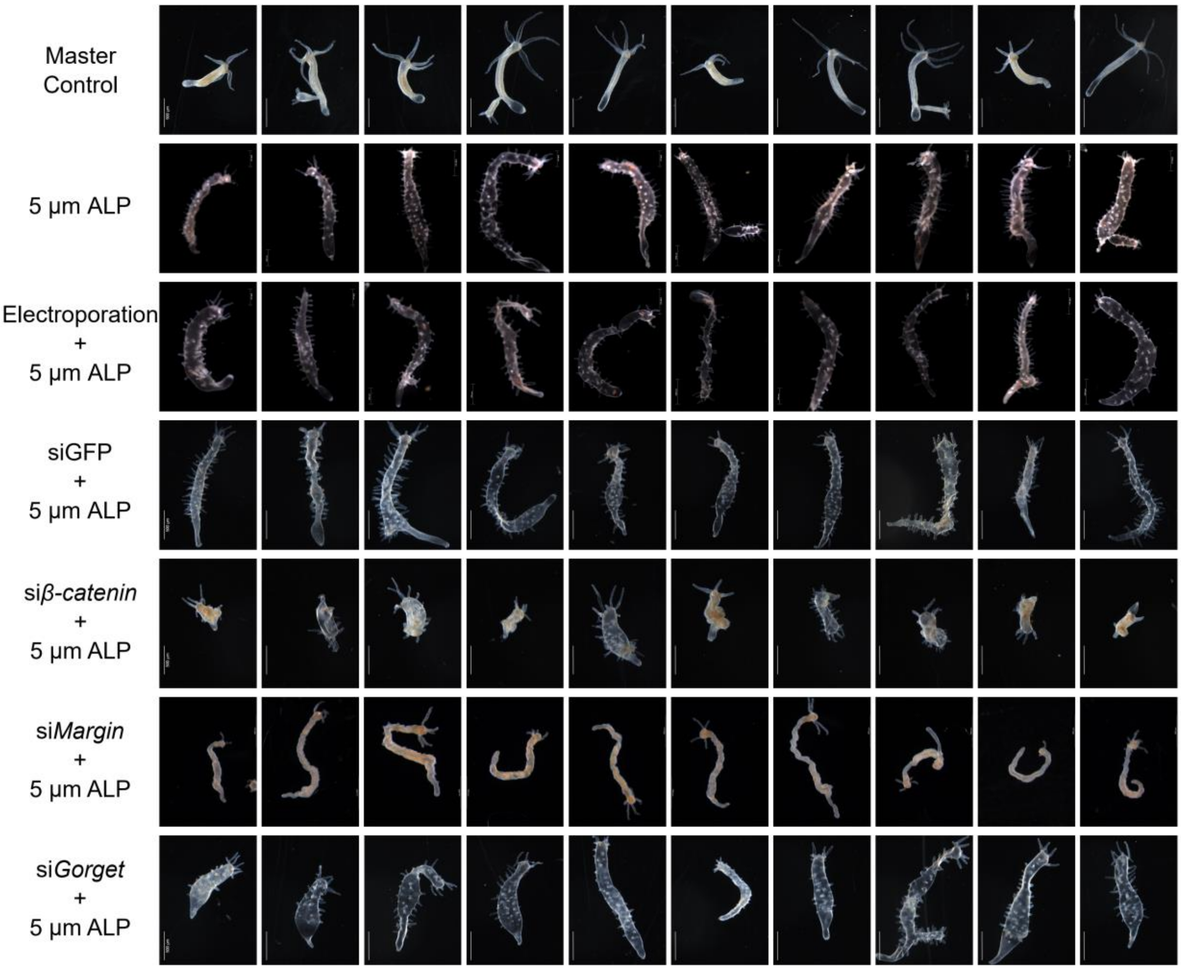

Supplementary Figure 8: Morphology of *Hydra* polyps following Knockdown of *β-catenin*, *Margin* and *Gorget*. Pictures of morphological changes followed by knockdown of *β-catenin*, *Margin* and *Gorget* in the background of Alsterpaullone treatment.

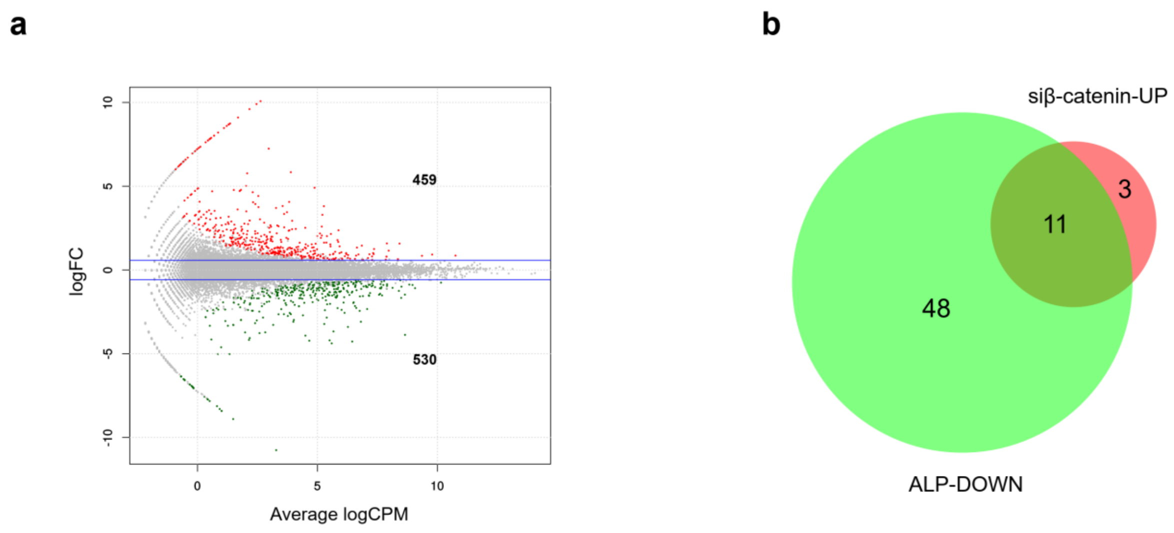

Supplementary Figure 9: Differential gene expression analysis upon *β-catenin* knockdown. a, Smear plot showing the differentially expressed genes upon knockdown of *β-catenin* followed by activation of Wnt signalling. Red and Green colour dots denote significantly up and down regulated genes respectively. **b,** Venn diagram showing the overlap of TFs downregulated by activation of Wnt signalling and TFs upregulated by *β-catenin* knockdown. ALP-DOWN, downregulated TFs upon Alsterpaullone treatment; siβ-catenin-UP, upregulated TFs after *β-catenin* knockdown.

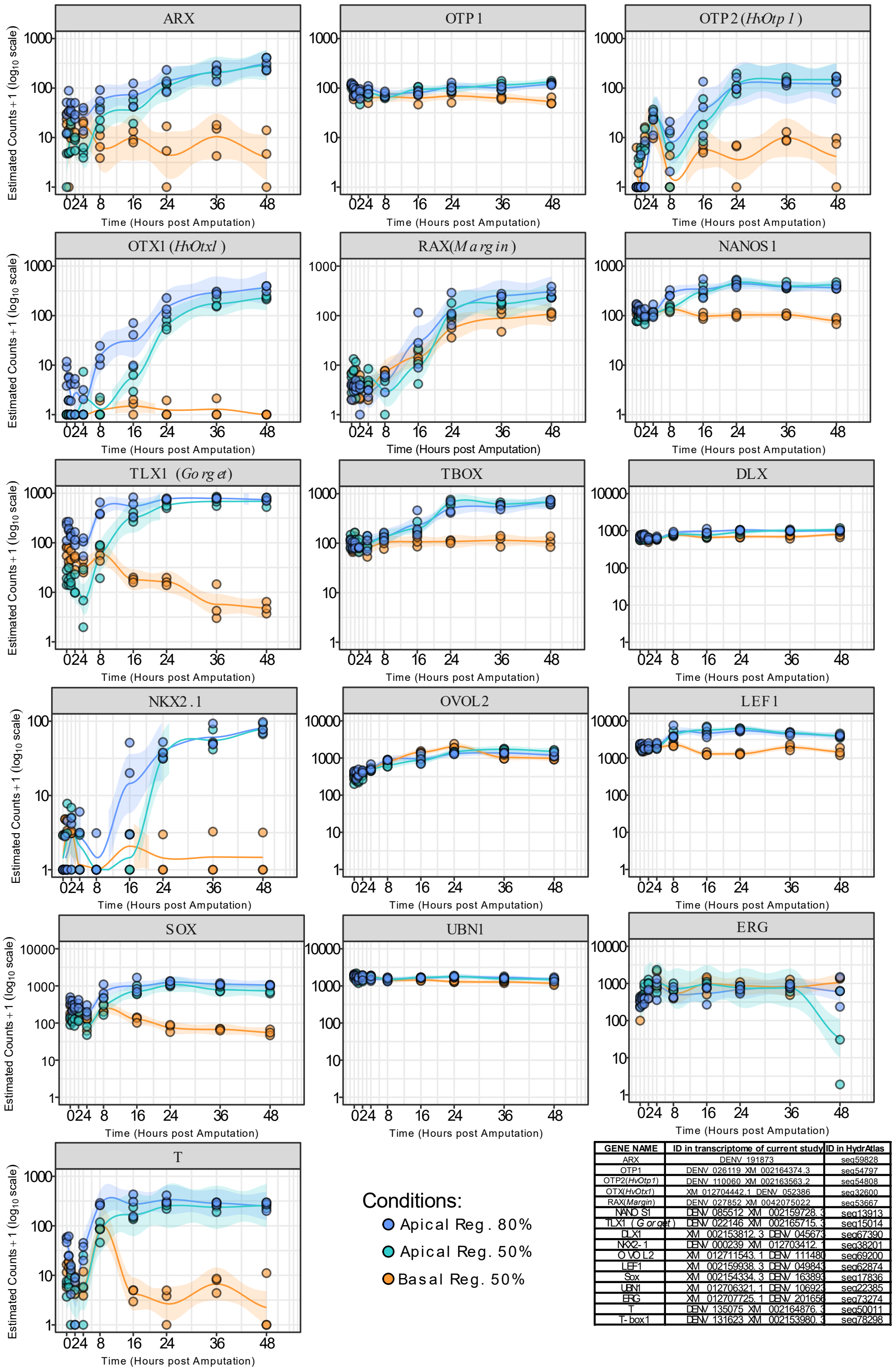

GENE NAME	ID in transcriptome of current study	ID in HydrAtlas
ARX	DENV 191873	seq59828
OTP1	DENV 026119 XM 002164374.3	seq54797
OTP2(*HvOtp1*)	DENV 110060 XM 002163563.2	seq54808
OTX(*HvOtx1*)	XM 012704442.1 DENV 052386	seq32600
RAX(*Margin*)	DENV 027852 XM 0042075022	seq53667
NANOS1	DENV 085512 XM 002159728.3	seq13913
TLX1 (*Gorget*)	DENV 022146 XM 002165715.3	seq15014
DLX1	XM 002153812.3 DENV 045673	seq67390
NKX2-1	DENV 000239 XM 012703412.1	seq38201
OVOL2	XM 012711543.1 DENV 111480	seq69200
LEF1	XM 002159938.3 DENV 049843	seq62874
Sox	XM 002154334.3 DENV 163893	seq17836
UBN1	XM 012706321.1 DENV 106923	seq22385
ERG	XM 012707725.1 DENV 201656	seq73274
T	DENV 135075 XM 002164876.3	seq50011
T-box1	DENV 131623 XM 002153980.3	seq78298

Supplementary Figure 10: Expression dynamics of Wnt/β-catenin regulated TFs identified in this study during regeneration of Hydra. Expression plots for selected genes were extracted from HydrATLAS server (https://hydratlas.unige.ch/)[2,3]. Plots depict the estimated counts (y-axis) for transcripts during different time points of regeneration (y-axis). The table in the bottom right corner includes details of gene names, identification numbers (IDs) of our hybrid transcriptome assembly and corresponding IDs in HydrATLAS.

Information regarding the versions of programs and command lines used in the *de novo* transcriptome assembly, functional annotation and differential expression analysis.

1) Data processing by FASTX toolkit (version 36.06)

I) Trimming first 10 and last 2 bases using fastx_trimmer

$fastx_trimmer -Q 33 -f 10 -l 99 -i $file -o $file.trimmed

II) Read correction using SEECER

$seecer Read1.fastq Read2.fastq

2) Transcriptome assembly by abyss-pe (version 3.81) and transAbyss (version 1.4.8)

I) Assembly at different k-mers (35 to 88 in the interval of 3)

```
#!/bin/bash
for k in {35..88..3}
do
echo "Processing k-mer ******** : $k"
mkdir k$k
echo "Running k-mer: $k" >> kmer.log
abyss-pe -C k$k np=80 name=illumina k=$k lib='VC_rep1 VC_rep2 AP_rep1
AP_rep2' \
VC_rep1='/data/transcriptome/SO_2135/VC_control/rep1/SO_2135_Set_I_B_VC_R
1.fastq.trimmed_corrected.fa
/data/transcriptome/SO_2135/VC_control/rep1/SO_2135_Set_I_B_VC_R2.fastq.trim
med_corrected.fa' \
VC_rep2='/data/transcriptome/SO_2135/VC_control/rep2/SO_2135_Set_II_A_VC_R
1.fastq.trimmed_corrected.fa
/data/transcriptome/SO_2135/VC_control/rep2/SO_2135_Set_II_A_VC_R2.fastq.tri
mmed_corrected.fa' \
AP_rep1='/data/transcriptome/SO_2135/AP_treated/rep1/SO_2135_Set_I_B_AP_R
1.fastq.trimmed_corrected.fa
/data/transcriptome/SO_2135/AP_treated/rep1/SO_2135_Set_I_B_AP_R2.fastq.trim
med_corrected.fa' \
AP_rep2='/data/transcriptome/SO_2135/AP_treated/rep2/SO_2135_Set_II_B_AP_R
1.fastq.trimmed_corrected.fa
/data/transcriptome/SO_2135/AP_treated/rep2/SO_2135_Set_II_B_AP_R2.fastq.tri
mmed_corrected.fa'
done
```

II) Filtering of the assemblies (FEM)

Transcriptome libraries:

• junction contigs and indel bubbles are extended

• short contigs and short islands are removed
• overlapping/redundant sequences are merged
• sequences shorter than read length are removed

```
for k in 35 38 41 44 47 50 53 56 59 62 65 68 73 78 83 88
do
        bash ../wrappers/abyss-ta-filter -n illumina -i illumina/trans-abyss-v1.4.8/assembly/k$k -o illumina/trans-abyss-v1.4.8/filter/k$k -k $k
done
```

```
bash ../wrappers/abyss-rmdups-iterative -n illumina -i illumina/trans-abyss-v1.4.8/filter/ -o illumina/trans-abyss-v1.4.8/merge/ -p 80
```

3) Prediction of ORFs from the transcripts

```
$perl /home/amol/trinityrnaseq_r2012-10-05/trinity-plugins/transdecoder/transcripts_to_best_scoring_ORFs.pl -t traabyss_transcripts.fa -m 50 --CPU 30
```

4) *De novo* transcript clustering

The UCLUST algorithm divides a set of sequences into clusters. The cluster_fast and cluster_smallmem commands are based on UCLUST. A cluster is defined by one sequence, known as the centroid or representative sequence. Every sequence in the cluster must have similarity above a given identity threshold with the centroid, as shown in the figure below.

```
$./usearch7.0.1001_i86linux32 -cluster_fast best_candidates.eclipsed_orfs_removed.pep -id 0.9 -centroids centroid.fasta -uc clusters.uc -fastapairs fastapairs.txt -userout hits.m8 -userfields query+target+id+alnlen+mism+opens+qlo+qhi+tlo+thi+evalue+bits
```

5) Prediction of ORFs from the reference based transcripts

```
$perl /home/amol/trinityrnaseq_r2012-10-05/trinity-plugins/transdecoder/transcripts_to_best_scoring_ORFs.pl -t reference_transcripts.fa -m 50 --CPU 30
```

6) Merging *de novo* (*Hydra vulgaris* Ind-Pune) and RefSeq (*Hydra magnipapillata*) assemblies

#refseq source

ftp://ftp.ncbi.nlm.nih.gov/genomes/refseq/invertebrate/Hydra_vulgaris/latest_assembly_versions/GCF_000004095.1_Hydra_RP_1.0/

#make blast database

```
$makeblastdb -in ./blastdb/refseq_protein.fasta -dbtype prot -out ./blastdb/refseq_protein

$makeblastdb -in ./blastdb/denovo_pep.fasta -dbtype prot -out ./blastdb/denovo_pep

$makeblastdb -in ./blastdb/uniref90.fasta -dbtype prot -out ./blastdb/uniref90
```

#run BLASTP reciprocally

```
$blastp -num_threads 12 -query ./blastdb/denovo_pep.fasta -db ./blastdb/refseq_protein -out ./RBH/denovo_vs_refseq -evalue 0.01 -outfmt 7 -max_target_seqs 1;

$blastp -num_threads 12 -query ./blastdb/refseq_protein.fasta -db ./blastdb/denovo_pep -out ./RBH/refseq_vs_denovo -evalue 0.01 -outfmt 7 -max_target_seqs 1
```

#select top hits

```
$awk '/^DENV*/' ./RBH/denovo_vs_refseq|awk '{if($3>=70) print$0}'|awk '{print$1,$2}'|sort -u > ./RBH/denovo_refseq_table

$awk '/^NP|^XP/' ./RBH/refseq_vs_denovo|awk '{if($3>=70) print$0}'|awk '{print$1,$2}'|sort -u > ./RBH/refseq_denovo_table
```

select same pair

```
$awk 'NR==FNR{a[$1,$2];next} ($2,$1) in a' ./RBH/denovo_refseq_table ./RBH/refseq_denovo_table > ./RBH/selected_matches
```

calculate the length of the protein sequences of denovo assembly and refseq

```
$awk '/^>/ {if (seqlen)print seqlen;print;seqlen=0;next} { seqlen+= length($0)}END{print seqlen}' ./blastdb/denovo_pep.fasta | awk 'NR%2{s=$0; next} {print s,$0}' OFS="\t" > ./RBH/denovo_pep_length

$awk '/^>/ {if (seqlen)print seqlen;print;seqlen=0;next} { seqlen+= length($0)}END{print seqlen}' ./blastdb/refseq_protein.fasta| awk 'NR%2{s=$0; next} {print s,$0}' OFS="\t" > ./RBH/refseq_protein_length
```

calculate the RBH ratio and select better covered refseq

```
$join -j 1 -o 1.2,1.1,2.2 <(sort -k1 ./RBH/selected_matches) <(sort -k1
./RBH/refseq_protein_length_mod) > ./RBH/selected_matches_refseq_length
```

```
$join -j 1 -o 1.1,1.2,2.2,1.3 <(sort -k1 ./RBH/selected_matches_refseq_length) <(sort
-k1 ./RBH/denovo_pep_length)|awk '{ratio=$4/$3; if(ratio>1) print $0, ratio}' >
./RBH/selected_matches_sigRBHratio_refseq
```

get the protein fasta sequences from the selected refseqIDs [RBH ratio based]

```
$awk '{print $2}' ./RBH/selected_matches_sigRBHratio_refseq >
./RBH/finla_assembly/selected_matches_sigRBHratio_refseq_IDs
```

#fetch FASTA sequences with better RBH ratio from refseq data

```
$awk
'BEGIN{while((getline<"./RBH/finla_assembly/selected_matches_sigRBHratio_refseq
_IDs")>0)l[">"$1]=1}/^>/{f=l[$1]?1:0}f' ./blastdb/refseq_protein.fasta >
./RBH/finla_assembly/sigRBHratio_refseq_protein.fasta
```

#fetch FASTA sequences unique to refseq data by romoving matching IDs (orthologs from RBH)

```
$awk
'BEGIN{while((getline<"./RBH/selected_matches")>0)l[">"$1]=1}/^>/{f=!l[$1]?1:0}f'
./blastdb/refseq_protein.fasta> ./RBH/finla_assembly/unique_refseq.fasta
```

#fetch FASTA sequences common to *de novo* and refseq data [from denovo assembly] (orthologs from RBH)

```
$awk
'BEGIN{while((getline<"./RBH/selected_matches")>0)l[">"$2]=1}/^>/{f=l[$1]?1:0}f'
./blastdb/denovo_pep.fasta > ./RBH/finla_assembly/common_denovo.fasta
```

#fetch FASTA sequences common to denovo and refseq data after filtering significant RBH hits from refseq [from *de novo* assembly] (orthologs from RBH)

```
$awk
'BEGIN{while((getline<"./RBH/selected_matches_sigRBHratio_refseq")>0)l[">"$1]=1}
/^>/{f=!l[$1]?1:0}f' ./RBH/finla_assembly/common_denovo.fasta >
./RBH/finla_assembly/common_denovo_denovo.fasta
```

#fetch FASTA sequences unique to *de novo* data by romoving matching IDs (orthologs from RBH)

```
$awk
'BEGIN{while((getline<"./RBH/selected_matches")>0)l[">"$2]=1}/^>/{f=!l[$1]?1:0}f'
./blastdb/denovo_pep.fasta > ./RBH/finla_assembly/unique_denovo.fasta
```

get the CDS fasta sequences from the selected refseqIDs [RBH ratio based]

```
$join -1 3 -2 1 -o 1.2 <(sort -k3
~/mouli/projects/hydra_regeneration/hmag_genome/ID_maps/IDs_map_mod) <(sort
```

```
-k1 ./RBH/finla_assembly/selected_matches_sigRBHratio_refseq_IDs) |sort -u >
./RBH/finla_assembly/selected_matches_sigRBHratio_refseq_XM_NM_IDs
```

#fetch FASTA sequences with better RBH ratio from refseq data

```
$awk
'BEGIN{while((getline<"./RBH/finla_assembly/selected_matches_sigRBHratio_refseq
_XM_NM_IDs")>0)l[">"$1]=1}/^>/{f=l[$1]?1:0}f' ./blastdb/refseq_rna.fasta >
./RBH/finla_assembly/sigRBHratio_refseq_cds.fasta
```

```
$join -1 3 -2 1 -o 1.2 <(sort -k3
~/mouli/projects/hydra_regeneration/hmag_genome/ID_maps/IDs_map_mod) <(sort
-k1 ./RBH/selected_matches) |sort -u > ./RBH/selected_matches_XM_NM_IDs
```

#fetch FASTA sequences unique to refseq data by romoving matching IDs (orthologs from RBH)

```
$awk
'BEGIN{while((getline<"./RBH/selected_matches_XM_NM_IDs")>0)l[">"$1]=1}/^>/{f=!
l[$1]?1:0}f' ./blastdb/refseq_rna.fasta >
./RBH/finla_assembly/unique_refseq_cds.fasta
```

#fetch FASTA sequences common to *de novo* and refseq data [from denovo assembly] (orthologs from RBH)

```
$grep ">" ./RBH/finla_assembly/common_denovo.fasta| awk '{split($1,a,">"); print
a[2]}' > ./RBH/finla_assembly/common_denovo_IDs
```

```
$awk
'BEGIN{while((getline<"./RBH/finla_assembly/common_denovo_IDs")>0)l[">"$1]=1}/^
>/{f=l[$1]?1:0}f' ./blastdb/denovo_cds.fasta >
./RBH/finla_assembly/common_denovo_cds.fasta
```

#fetch FASTA sequences common to *de novo* and refseq data after filtering significant RBH hits from refseq [from *de novo* assembly] (orthologs from RBH)

```
$join -1 3 -2 2 -o 2.1 <(sort -k3
~/mouli/projects/hydra_regeneration/hmag_genome/ID_maps/IDs_map_mod) <(sort
-k2 ./RBH/selected_matches_sigRBHratio_refseq) |sort -u >
./RBH/finla_assembly/selected_matches_sigRBHratio_denovo_IDs
```

```
$awk
'BEGIN{while((getline<"./RBH/finla_assembly/selected_matches_sigRBHratio_denov
o_IDs")>0)l[">"$1]=1}/^>/{f=!l[$1]?1:0}f'
./RBH/finla_assembly/common_denovo_cds.fasta >
./RBH/finla_assembly/common_denovo_denovo_cds.fasta
```

#fetch FASTA sequences unique to denovo data by romoving matching IDs (orthologs from RBH)

```
$awk
'BEGIN{while((getline<"./RBH/selected_matches")>0)l[">"$2]=1}/^>/{f=!l[$1]?1:0}f'
./blastdb/denovo_cds.fasta > ./RBH/finla_assembly/unique_denovo_cds.fasta
```

remove line breaks to protein fasta files

```
$awk '!/^>/ { printf "%s", $0; n = "\n" }  /^>/ { print n $0; n = "" } END { printf "%s", n }'
./RBH/finla_assembly/sigRBHratio_refseq_protein.fasta >
./RBH/finla_assembly/sigRBHratio_refseq_protein_nolinebreaks.fasta
```

```
$awk '!/^>/ { printf "%s", $0; n = "\n" }  /^>/ { print n $0; n = "" } END { printf "%s", n }'
./RBH/finla_assembly/unique_refseq.fasta>./RBH/finla_assembly/unique_refseq_nili
nebreak.fasta
```

```
$awk '!/^>/ { printf "%s", $0; n = "\n" }  /^>/ { print n $0; n = "" } END { printf "%s", n }'
./RBH/finla_assembly/common_denovo.fasta >
./RBH/finla_assembly/common_denovo_nolinebreak.fasta
```

```
$awk '!/^>/ { printf "%s", $0; n = "\n" }  /^>/ { print n $0; n = "" } END { printf "%s", n }'
./RBH/finla_assembly/common_denovo_denovo.fasta >
./RBH/finla_assembly/common_denovo_denovo_nolinebreaks.fasta
```

```
$awk '!/^>/ { printf "%s", $0; n = "\n" }  /^>/ { print n $0; n = "" } END { printf "%s", n }'
./RBH/finla_assembly/unique_denovo.fasta >
./RBH/finla_assembly/unique_denovo_nolinebreak.fasta
```

```
$awk '!/^>/ { printf "%s", $0; n = "\n" }  /^>/ { print n $0; n = "" } END { printf "%s", n }'
./blastdb/denovo_pep.fasta > ./RBH/finla_assembly/denovo_pep_nolinebreaks.fasta
```

remove line breaks to CDS fasta files

```
$awk '!/^>/ { printf "%s", $0; n = "\n" }  /^>/ { print n $0; n = "" } END { printf "%s", n }'
./RBH/finla_assembly/sigRBHratio_refseq_cds.fasta >
./RBH/finla_assembly/sigRBHratio_refseq_cds_nolinebreaks.fasta
```

```
$awk '!/^>/ { printf "%s", $0; n = "\n" }  /^>/ { print n $0; n = "" } END { printf "%s", n }'
./RBH/finla_assembly/unique_refseq_cds.fasta >
./RBH/finla_assembly/unique_refseq_cds_nolinebreaks.fasta
```

```
$awk '!/^>/ { printf "%s", $0; n = "\n" }  /^>/ { print n $0; n = "" } END { printf "%s", n }'
./RBH/finla_assembly/common_denovo_cds.fasta >
./RBH/finla_assembly/common_denovo_cds_nolinebreaks.fasta
```

```
$awk '!/^>/ { printf "%s", $0; n = "\n" }  /^>/ { print n $0; n = "" } END { printf "%s", n }'
./RBH/finla_assembly/common_denovo_denovo_cds.fasta >
./RBH/finla_assembly/common_denovo_denovo_cds_nolinebreaks.fasta
```

```
$awk '!/^>/ { printf "%s", $0; n = "\n" }  /^>/ { print n $0; n = "" } END { printf "%s", n }'
./RBH/finla_assembly/unique_denovo_cds.fasta >
./RBH/finla_assembly/unique_denovo_cds_nolinebreaks.fasta
```

modify protein fasta headers of refseq [remove description and ">" and keep IDs]

```
$sed 's/\ .*$//g'
./RBH/finla_assembly/sigRBHratio_refseq_protein_nolinebreaks.fasta|sed 's/>//g' >
./RBH/finla_assembly/sigRBHratio_refseq_protein_nolinebreaks_modIDs.fasta
```

modify cds fasta headers of refseq [remove description and ">" and keep IDs]

```
$sed 's/\ .*$//g' ./RBH/finla_assembly/sigRBHratio_refseq_cds_nolinebreaks.fasta|
sed 's/>//g' >
./RBH/finla_assembly/sigRBHratio_refseq_cds_nolinebreaks_modIDs.fasta
```

```
$sed 's/\ .*$//g' ./RBH/finla_assembly/unique_refseq_cds_nolinebreaks.fasta|sed
's/>//g' > ./RBH/finla_assembly/unique_refseq_cds_nolinebreaks_modIDs.fasta
```

convert no line break protein fasta to tab limited

```
$awk 'NR%2{s=$0; next} {print s,$0}' OFS="\t"
./RBH/finla_assembly/sigRBHratio_refseq_protein_nolinebreaks_modIDs.fasta >
./RBH/finla_assembly/sigRBHratio_refseq_protein_nolinebreaks_modIDs_tablimited.
fasta
```

```
$sed 's/>//g' ./RBH/finla_assembly/common_denovo_denovo_nolinebreaks.fasta
|awk 'NR%2{s=$0; next} {print s,$0}' OFS="\t"
>./RBH/finla_assembly/common_denovo_denovo_nolinebreaks_tablimited.fasta
```

```
$awk'NR%2{s=$0; next} {print s,$0}' OFS="\t"
./RBH/finla_assembly/denovo_pep_nolinebreaks.fasta >
./RBH/finla_assembly/denovo_pep_nolinebreaks_tablimited.fasta
```

convert no line break cds fasta to tab limited

```
$awk 'NR%2{s=$0; next} {print s,$0}' OFS="\t"
./RBH/finla_assembly/sigRBHratio_refseq_cds_nolinebreaks_modIDs.fasta>
./RBH/finla_assembly/sigRBHratio_refseq_cds_nolinebreaks_modIDs_tablimited.fast
a
```

```
$sed 's/>//g'
./RBH/finla_assembly/common_denovo_denovo_cds_nolinebreaks.fasta| awk
'NR%2{s=$0; next} {print s,$0}' OFS="\t" >
./RBH/finla_assembly/common_denovo_denovo_cds_nolinebreaks_tablimited.fasta
```

add *de novo* IDs to RBH best hit IDs (protein)

```
$join -j 1 -o 1.1,1.2,2.2 <(sort -k1 ./RBH/selected_matches) <(sort -k1
./RBH/finla_assembly/sigRBHratio_refseq_protein_nolinebreaks_modIDs_tablimited.
fasta) | awk '{print">"$1"_"$2,$3}' OFS="\n" >
./RBH/finla_assembly/sigRBHratio_refseq_proteinID_denovoID.fasta
```

add *de novo* IDs to RBH best hit IDs (cds)

```
$join -1 3 -2 1 -o 1.2 2.2 <(sort -k3
~/mouli/projects/hydra_regeneration/hmag_genome/ID_maps/IDs_map_mod) <(sort
-k1 ./RBH/selected_matches) |sort -u > ./RBH/selected_matches_NM_XM_denv_IDs
```

```
$join -j 1 -o 1.1,1.2,2.2 <(sort -k1 ./RBH/selected_matches_NM_XM_denv_IDs)
<(sort -k1
./RBH/finla_assembly/sigRBHratio_refseq_cds_nolinebreaks_modIDs_tablimited.fast
a) | awk '{print">"$1"_"$2,$3}' OFS="\n" >
./RBH/finla_assembly/sigRBHratio_refseqID_denovoID.fasta
```

add refseqIDs to *de novo* matching sequences (protein)

```
$join -1 2 -2 1 -o 1.2,1.1,2.2 <(sort -k2 ./RBH/selected_matches) <(sort -k1
./RBH/finla_assembly/common_denovo_denovo_nolinebreaks_tablimited.fasta) |
awk '{print">"$1"_"$2,$3}' OFS="\n" >
./RBH/finla_assembly/common_denovo_refseq.fasta
```

add refseqIDs to *de novo* matching sequences (cds)

```
$join -1 2 -2 1 -o 1.2,1.1,2.2 <(sort -k2 ./RBH/selected_matches_NM_XM_denv_IDs)
<(sort -k1
./RBH/finla_assembly/common_denovo_denovo_cds_nolinebreaks_tablimited.fasta)
| awk '{print">"$1"_"$2,$3}' OFS="\n" >
./RBH/finla_assembly/common_denovoID_refseqID.fasta
```

#merge all sequences

```
$cat ./RBH/finla_assembly/protein_sequences/*.* >
./RBH/finla_assembly/protein_sequences/hybrid_protein.fasta
```

```
$cat ./RBH/finla_assembly/CDS_sequences/*.* >
./RBH/finla_assembly/CDS_sequences/hybrid_cds.fasta
```

7) Calculation of completeness of different transcriptome assemblies by BUSCO (version 3.0.2)

a) training augustus

Initially gene prediction algorithm was trained to get custom specific models for running AGUSTUS. This was achieved through BUSCO wrapper. Here, I have used filtered gene sets from RefSeq genome annotation containing multi exon and single copy genes after removing UTRs.

split test and training set (~1:5)

```
$./scripts/randomSplit.pl ./gene_set_filter4_mod_withoutUTR.gb 200
grep -c LOCUS
./gene_filter4_mod_withoutUTR.gb*
```

create new file for hydra vulgaris Ind-Pune

```
$./scripts/new_species.pl --species=HvulIP
```

#etrain

$./bin/etraining --species=HvulIP ./ gene_set_filter4_mod_withoutUTR.gb.train

#test
$./bin/augustus --species=HvulIP ./ gene_set_filter4_mod_withoutUTR.gb.test | tee firsttest.out

#optimize
$./scripts/optimize_augustus.pl --cpus=10 --species=HvulIP ./MAKER_filter4_mod_withoutUTR.gb.train --metapars=./config/species/HvulIP/HvulIP_metapars.cfg

b) Run BUSCO for each transcriptome assembly or refseq data

#busco
$./scripts/run_BUSCO.py -c 10 --long -sp HvulIP -o Hmag1 -i /home/hydra/mouli/projects/Hydra_Genome/BUSCO/busco/data/hma.fasta -l /home/hydra/mouli/projects/Hydra_Genome/BUSCO/busco/data/eukaryota_odb9 -m genome -o out_hmag1

$python scripts/generate_plot.py -wd ./BUSCO_summaries_hydra_assemblies

8) Functional annotation by InterProScan (version 5.14-53.0) and BLAST (version 2.2.28+)

functional annotation by InterProScan

$./interproscan-5.14-53.0/interproscan.sh -i /home/hydra/mouli/projects/wnt/new_analysis/denovo_refseq_merge/RBH/finla_assembly/protein_sequences/hybrid_protein.fasta -appl ProDom,ProSiteProfiles,PRINTS,Pfam,TIGRFAM --goterms -o /home/hydra/mouli/projects/wnt/new_analysis/denovo_refseq_merge/annotation/hybrid_protein_interproscan -f TSV

$awk 'BEGIN {FS="\t"};{print$1"\t"$14}' ./hybrid_protein_interproscan | awk '/GO:/{print$0}'| sort -u > ./hybrid_protein_interproscan_go_raw

$awk '{FS="\t"} {for(j=2;j<=NF;j++) a[$1]=a[$1]"\t"$j}END{for(i in a) {print i""a[i]}}' ./annotation/hybrid_protein_interproscan_go_raw > ./annotation/hybrid_protein_interproscan_go_WEGO_native_format

#remove repeted GO IDs per record

$awk '{ while(++i<=NF) printf (!a[$i]++) ? $i FS:""; i=split("",a); print ""}' ./annotation/hybrid_protein_interproscan_go_WEGO_native_format > ./annotation/hybrid_protein_interproscan_go_WEGO_native_format_mod

annotation by performing BLAST against ubniref90

```
$blastp -query ./RBH/finla_assembly/protein_sequences/hybrid_protein.fasta -db
./blastdb/uniref90 -num_threads 11 -max_target_seqs 1 -outfmt 6 >
./annotation/hybrid_protein_blastp_uniref90.outfmt6
```

```
$awk '{print $2}' ./annotation/hybrid_protein_blastp_uniref90.outfmt6 >
./annotation/hybrid_protein_blastp_hits_uniref90_IDs
```

```
#go to uniprot retrieve/ID mapping and paste the list of UniRef90 hits and converts
IDs (UniRef) to UniProtKB, download as tab limited file and change UNIREF90 to
UniRef90.
```

```
$join -1 2 -2 1 -o 1.1 2.4 -t $'\t' <(sort -k2
./annotation/hybrid_protein_blastp_uniref90.outfmt6) <(sort -k1
./annotation/uniref90_go_IDs.tab)| awk '/GO:/{print $0}' |awk '$1=$1' FS=";" OFS="\t"
|sort -u >./annotation/hybrid_protein_uniref90blastP_go_raw
```

```
$awk '{FS="\t"} {for(j=2;j<=NF;j++) a[$1]=a[$1]"\t"$j}END{for(i in a) {print i""a[i]}}'
./annotation/hybrid_protein_uniref90blastP_go_raw >
./annotation/hybrid_protein_uniref90blastP_go_raw_WEGO_native_format
```

```
#remove repeated GO IDs per record
```

```
$awk '{ while(++i<=NF) printf (!a[$i]++) ? $i FS:""; i=split("",a); print ""}'
./annotation/hybrid_protein_uniref90blastP_go_raw_WEGO_native_format >
./annotation/hybrid_protein_uniref90blastP_go_raw_WEGO_native_format_mod
```

```
$cat
./annotation/hybrid_protein_uniref90blastP_go_raw_WEGO_native_format_mod
./annotation/hybrid_protein_interproscan_go_WEGO_native_format_mod >
./annotation/merged_GO_IDs
```

```
$awk '{FS=" "} {for(j=2;j<=NF;j++) a[$1]=a[$1]"\t"$j}END{for(i in a) {print i""a[i]}}'
./annotation/merged_GO_IDs > ./annotation/merged_GO_IDs_unique
```

```
#remove repeated GO IDs per record
```

```
$awk '{ while(++i<=NF) printf (!a[$i]++) ? $i FS:""; i=split("",a); print ""}'
./annotation/merged_GO_IDs_unique > ./annotation/merged_GO_IDs_mod
```

9) **Read alignment and quantitation by Trinity pipeline (version 2.1.1)**

```
#!/bin/bash

for i in $(ls
/home/hydra/mouli/projects/wnt/new_analysis/refseqbased_DE/tophat/*.fastq|awk -
F"/" '{split($NF,a,"."); print a[1]}'|cut -c -11| sort -u)

do ./util/align_and_estimate_abundance.pl --thread_count 10 --transcripts
/home/hydra/mouli/projects/wnt/new_analysis/trinity_DE/hybrid_cds_mod.fasta --
seqType fq --left
/home/hydra/mouli/projects/wnt/new_analysis/refseqbased_DE/tophat/${i}_R1.fastq -
-right
```

/home/hydra/mouli/projects/wnt/new_analysis/refseqbased_DE/tophat/${i}_R2.fastq --est_method RSEM --aln_method bowtie --prep_reference --output_dir /home/hydra/mouli/projects/wnt/new_analysis/trinity_DE/alignment_out/${i}

done;

Note: similar command line was used for analysis using different experimental samples. The final count matrix was used in edgeR for differential expression analysis. All the commands listed above are generic and require additional details (directory structure and file names) in order to re-execute them.

Phylogenetic analysis of PRD class of homeobox genes.

Phylogenetic analysis was carried out using the homeobox genes collected from HomeoDB for human, mouse, zebrafish, fruitfly and nematode [4](http://homeodb.zoo.ox.ac.uk). Sequences from *Nematostella* and sponges were included for comparative analysis based on previous classification [5]. Here, initial broad classification was carried out using CLANS to segregate PRD class members based on BLAST similarity scores using bioinformatic toolkit available at htpps://toolkit.tubingen.mpg.de [6]. Homeobox domain regions were collected from these sequences after performing hmmscan using HMMER v3.1. All the fetched sequences from these group were aligned using ETC3 tool kit [7]. These alignments were cured manually in Jalview v2 and used for further analysis (Waterhouse et. al., 2009). Phylogentic tree was computed using randomized accelerated maximum likelihood (RAxML-HPC v.8 on XSEDE) implemented in CIPRES Science Gateway [8]. Here, protein CAT model with LG substitution matrix was used with 1000 iterations of rapid bootstrap analysis and HyHox1 is used as an outgroup. The phylogenetic tree was visualized and annotated in iTOL and branch support values above 30 were displayed on branches [9].

1) PRD class protein sequence files

```
>al_DR

MGISEEIKLEELPQEAKLAHPDAVVLVDRAPGSSAASAGAALTVSMSVSGGAPSGASGASGGTNSPVSDGNSDCE
ADEYAPKRKQRRYRTTFTSFQLEELEKAFSRTHYPDVFTREELAMKIGLTEARIQVWFQNRRAKWRKQEKVGPQS
HPYNPYLPGGAATMQTVVGAALPPNPFTHLGFQLRKPFDAQHAANLAAFRYPHLSAAPMIPSGYFNQFQRAPPHM
LPHGMAGMYSPSSSFQSLLANMTAVPRGTPLGKPPALLVGSPDLHSPNHMLASPPTSPASGHASQHQQHPTAHPP
PPQAPPQMPVGVQPAQLSPQHLVGIALTQQASSLSPTQTSPVALTLSHSPQRQLPPPSHQAPPPPPRAATPPEDR
RTSSIAALRLKAREHELKLELLRQNGHGNDVVS

>ALX1_DA

MEYLSDKFSLKSPAIKGSDYYMDQVMDTLDNVQYYNKASPKCVQAFPMQSNDQHSSMDRSSPCDNQSSVTYCAPK
SEESSLHAMENCCSLRVSPATSGPDKTDLDELGEKCDSNVSSSKKRRHRTTFTSAQLEELEKVFQKTHYPDVYVR
EQLAMRTELTEARVQVWFQNRRAKWRKRERYGQIQQAKSHFAATYDISMLPRTDSYSQISNNLWTGPSAGSSVVS
SCMIPRGSPPCVTSPYPHSPRAAEHGYVGFPNHQQNQFGVNHVSLNNFFADSLLASSANSHAAFETKPEFERRSS
SIAVLRMKAKEHTANISWAM
```

>ALX1_HU

MEFLSEKFALKSPPSKNSDFYMGAGGPLEHVMETLDNESFYSKASAGKCVQAFGPLPRAEHHVRLERTSPCQDSS
VNYGITKVEGQPLHTELNRAMDNCNSLRMSPVKGMQEKGELDELGDKCDSNVSSSKKRRHRTTFTSLQLEELEKV
FQKTHYPDVYVREQLALRTELTEARVQVWFQNRRAKWRKRERYGQIQQAKSHFAATYDISVLPRTDSYPQIQNNL
WAGNASGGSVVTSCMLPRDTSSCMTPYSHSPRTDSSYTGFSNHQNQFSHVPLNNFFTDSLLTGATNGHAFETKPE
FERRSSSIAVLRMKAKEHTANISWAM

>ALX1_MO

MEFLSEKFALKSPPSKNSDFYMGTGGALEHVMETLDNESFYGKATAGKCVQAFGPLPRAEHHVRLDRTSPCQDSS
VNYGITKVEGQPLHTELNRAMDNCNNLRMSPVKGMPEKSELDELGDKCDSNVSSSKKRRHRTTFTSLQLEELEKV
FQKTHYPDVYVREQLALRTELTEARVQVWFQNRRAKWRKRERYGQIQQAKSHFAATYDISVLPRTDSYPQIQNNL
WAGNASGGSVVTSCMLPRDASSCMTPYSHSPRTDSSYTGFSNHQNQFSHVPLNNFFTDSLLTGATNGHAFETKPE
FERRSSSIAVLRMKAKEHTANISWAM

>ALX3_HU

MDPEHCAPFRVGPAPGPYVASGDEPPGPQGTPAAAPHLHPAPPRGPRLTRFPACGPLEPYLPEPAKPPAKYLQDL
GPGPALNGGHFYEGPAEAEEKTSKAASFPQLPLDCRGGPRDGPSNLQGSPGPCLASLHLPLSPGLPDSMELAKNK
SKKRRNRTTFSTFQLEELEKVFQKTHYPDVYAREQLALRTDLTEARVQVWFQNRRAKWRKRERYGKIQEGRNPFT
AAYDISVLPRTDSHPQLQNSLWASPGSGSPGGPCLVSPEGIPSPCMSPYSHPHGSVAGFMGVPAPSAAHPGIYSI
HGFPPTLGGHSFEPSSDGDYKSPSLVSLRVKPKEPPGLLNWTT

>ALX3_MO

MDPERCAPFSVGPAAGPYAAAGDEAPGPQGTPDAAPHLHPAPPRGPRLSRFPACGPLEPYLPEPAKPPAKYLQDL
GPGPVLNGGHFYEGSAEAEEKASKAASFPQLPVDCRGGPRDGPSNVQASPGPCLASLSVPLSPGLPDSMELAKTK
SKKRRNRTTFSTFQLEELEKVFQKTHYPDVYAREQLALRTDLTEARVQVWFQNRRAKWRKRERYGKMQEGRNPFT
TAYDISVLPRTDSHPQLQNSLWPSPGSGSPGGPCLMSPEGIPSPCMSPYSHSHGNVAGFMGVPASPAAHPGIYSI
HGFPPALGGHSFEPSPDGDYKSPSLVSLRMKPKEPPGLLNWTT

>ALX4_HU

MNAETCVSYCESPAAAMDAYYSPVSQSREGSSPFRAFPGGDKFGTTFLSAAAKAQGFGDAKSRARYGAGQQDLAT
PLESGAGARGSFNKFQPQPSTPQPQPPPQPQPQQQQPQPQPPAQPHLYLQRGACKTPPDGSLKLQEGSSGHSAAL
QVPCYAKESSLGEPELPPDSDTVGMDSSYLSVKEAGVKGPQDRASSDLPSPLEKADSESNKGKKRRNRTTFTSYQ
LEELEKVFQKTHYPDVYAREQLAMRTDLTEARVQVWFQNRRAKWRKRERFGQMQQVRTHFSTAYELPLLTRAENY
AQIQNPSWLGNNGAASPVPACVVPCDPVPACMSPHAHPPGSGASSVTDFLSVSGAGSHVGQTHMGSLFGAASLSP
GLNGYELNGEPDRKTSSIAALRMKAKEHSAAISWAT

>ALX4_MO

MNAETCVSYCESPAAAMDAYYSPVSQSREGSSPFRGFPGGDKFGTTFLSAGAKGQGFGDAKSRARYGAGQQDLAA
PLESSSGARGSFNKFQPQPPTPQPPPAPPAPPAHLYLQRGACKTPPDGSLKLQEGSGGHNAALQVPCYAKESNLG
EPELPPDSEPVGMDNSYLSVKETGAKGPQDRASAEIPSPLEKTDSESNKGKKRRNRTTFTSYQLEELEKVFQKTH
YPDVYAREQLAMRTDLTEARVQVWFQNRRAKWRKRERFGQMQQVRTHFSTAYELPLLTRAENYAQIQNPSWIGNN
GAASPVPACVVPCDPVPACMSPHAHPPGSGASSVSDFLSVSGAGSHVGQTHMGSLFGAAGISPGLNGYEMNGEPD
RKTSSIAALRMKAKEHSAAISWAT

>ALX4a_DA

MNAETCVSYCEMTSMDSYYSPSAPQGRDHQANPFRTFQASDTKYSPAFLTNKGQGYGEKSGSPFQQECQSLDATA
GEGTFNKYHLFMQRSSCKTPPDSSKLQQENSGHNGGLIACYGKDSTGLTDSELPQNSDPAGMDGSYLSVKDSGVK
SPQQATSELASPLDKTEGESNKGKKRRNRTTFTSYQLEELEKVFQKTHYPDVYAREQLALRTDLTEARVQVWFQN
RRAKWRKRERFGQMQQVRTHFSTAYELPLLTRPENYAQIQNPSWIGGSSAASPVPGCVVPCDSVTSCMPPHPHAA
SGVSDFLGVPSPGSHMGQTHMGSLFGSPGMGTGINGYDLNMDPDRKSSSIAALRMKAKEHSAAISWAT

>ARX_DA

MSSQYDDDSRDRSECKSKSPTVLSSYCIDSILGRRSPCKVRQLGAQSLPAPVRPDHEMTTEVTSKENSFDSDMHL
PPKLRRLYGPGGKYLDSGRGFHEHLEKGERERLLDQACESLKISQAPQVSISRSKSYRENAPFSQSDEGQSPEHM

AQELVELSTLKFEEDVVKEEACGDNSLSPKDEESLHNDGDVKDGEDSVCLSAGSDSEEGMLKRKQRRYRTTFTSY
QLEELERAFQKTHYPDVFTREELAMRLDLTEARVQVWFQNRRAKWRKREKAGVQAHPTGLPFPGPLAAAHPLSHY
LEGGPFPPHPHPALESAWTAAAAAAAAFPGLAPPPNSSALPPATPLGLGTFLGTAMFRHPAFIGPTFGRLFSSMG
PLTSASTAAALLRQTAPPVESPVQPSAALPEPPSSSSSTAADRRASSIAALRLKAKEHSAQLTQLNILPSGTAGK
EVC

>ARX_HU

MSNQYQEEGCSERPECKSKSPTLLSSYCIDSILGRRSPCKMRLLGAAQSLPAPLTSRADPEKAVQGSPKSSSAPF
EAELHLPPKLRRLYGPGGGRLLQGAAAAAAAAAAAAAAATATAGPRGEAPPPPPPTARPGERPDGAGAAAAAAAA
AAAAAWDTLKISQAPQVSISRSKSYRENGAPFVPPPPALDELGGPGGVTHPEERLGVAGGPGSAPAAGGGTGTED
DEEELLEDEEDEDEEEELLEDDEEELLEDDARALLKEPRRCPVAATGAVAAAAAAAVATEGGELSPKEELLLHPE
DAEGKDGEDSVCLSAGSDSEEGLLKRKQRRYRTTFTSYQLEELERAFQKTHYPDVFTREELAMRLDLTEARVQVW
FQNRRAKWRKREKAGAQTHPPGLPFPGPLSATHPLSPYLDASPFPPHHPALDSAWTAAAAAAAAAFPSLPPPPGS
ASLPPSGAPLGLSTFLGAAVFRHPAFISPAFGRLFSTMAPLTSASTAAALLRQPTPAVEGAVASGALADPATAAA
DRRASSIAALRLKAKEHAAQLTQLNILPGTSTGKEVC

>ARX_MO

MSNQYQEEGCSERPECKSKSPTLLSSYCIDSILGRRSPCKMRLLGAAQSLPAPLASRADQEKAMQGSPKSSSAPF
EAELHLPPKLRRLYGPGGGRLLQGAAAAAAAAAAAAAAATATGTAGPRGEVPPPPPPAARPGERQDSAGAVAAAA
AAAAWDTLKISQAPQVSISRSKSYRENGAPFVPPPPALDELSGPGGVAHPEERLSAASGPGSAPAAGGGTGAEDD
EEELLEDEEDEEEEEELLEDDDEELLEDDARALLKEPRRCSVATTGTVAAAAAAAAAAVATEGGELSPKEELLLH
PEDAEGKDGEDSVCLSAGSDSEEGLLKRKQRRYRTTFTSYQLEELERAFQKTHYPDVFTREELAMRLDLTEARVQ
VWFQNRRAKWRKREKAGAQTHPPGLPFPGPLSATHPLSPYLDASPFPPHHPALDSAWTAAAAAAAAAFPSLPPPP
GSASLPPSGAPLGLSTFLGAAVFRHPAFISPAFGRLFSTMAPLTSASTAAALLRQPTPAVEGAVASGALADPATA
AADRRASSIAALRLKAKEHAAQLTQLNILPGTSTGKEVC

>CG11294_DR

MAGPFISPLPGDLLTEYMFGRRRQRRNRTTFTPQQLQELEALFQKTHYPDVFLREEVALRISLSEARVQVWFQNR
RAKWRKQARLQLLQDAWRMRCLSLGTPPVMGGGAVQGGSGNGATARPPSQTPENLSSASKDSELAEVGNGPNSGS
FTMMHPAFQQQHQQQQHQGHQQATDQDKLSKTYTELKLYKAPSHGMELGGMAALSGHSDEGSDGSDSEEIDLTSG
ACIDFSQSSKLQQQQQQQQQQGTQGAAGSAEANGGL

>CG13141_DR

MNIEKTITFSDPKMSGLPTASITECTEDSNTPKLAPDKPLLPEECVSPEPSRNREHCDPLDTSLVNTKQRRSRTN
FTLDQLNELERLFEETHYPDAFMREELSQRLGLSEARVQVWFQNRRAKCRKHENQMHKGFLVGSRSPPIATPLEP
CRVAPYVSLAALRSSSVPSHPAAATSSNPQAPGVSGTSSSGKVVTVDSPHNHNPAISRSAIKQFSSTVAAAAAFS
AFDPAIISVAAHQYAAAITNGTVPAGLFSVPQYSINLAAFAAAHSKSSSIADLRMKAKKHSESLGLQADMVL

>CG32532_DR

MIRATSCALSTRPLRSPKAPSHRHKIRSIKTGARSPRDSFQLDYSIDFFKTYIDTQRFYAGHKLDFKHQSCAGGP
GSGGAGGPGPGGGGGASGVVVVGGTSNSNNPAAAQQQQQQQQQQQQQQQQQSGGNNPSNGSNSAVALSLAHPHAH
ASHAHSSHAHGSHAHALAIAHGHHGLLPSVVPQQQQQQQQQQQQQQQQQQAQQLPPGVGVASPLQQQQQQQQQQQ
QQQQQQQQQQQLINNGGVSAGTPTLLIGNGPSSSGAGTGSVSAAAAAAAAAAAASSSSSSLPHVAPHSLGLSPQS
SADSQQFNIFPAIFSRQLNFSAGGQGGKLSMDELRPNLVGGLLGLQQGLLEDHASMQHGGVGQDTKFMSFQDQRL
MGIGGSHENRLLSLASSVQDTRSPITTLEKSSSSSLNHQRKCSSTPEDFSALYSGLPTPGMDSSSHHHTPAHTPP
SRLSDHTISAEGAFKKLKPEPNSGLSTVSAGITSPGSGLSSLSQHAGHTPTTASCPTPARRRHRTTFTQEQLAEL
EAAFAKSHYPDIYCREELARTTKLNEARIQVWFQNRRAKYRKQEKQLQKALAPSVIPSCNGMMRNIQGYSVSRGY
QPYPHHNTMNRYPQDLFQMGASSYPGMTQPFSMAHSTNMGSVGVRQDSMGEFHGMSPEDEWYNKSLSALRMNSSH
HPNLSAPMLQYQT

>CG34367_DR

MEQLAQFVTKSFEFNLTNNSVHALFNSMTGARLKKPTEVKNSIFINSISSGGSYADTCASGEESVDIDITDLSVS
ESNTILAIDPEPTEESHSQREEHVETYSLSPTLHKAAVEPKPKNWLISEDLDVDSQPEDPKMSGLPTASITECTE
DSNTPKLAPDKPLLPEECVSPEPSRNREHCDPLDTSLVNTKQRRSRTNFTLDQLNELERLFEETHYPDAFMREEL
SQRLGLSEARVQVWFQNRRAKCRKHENQMHKGFLVGSRSPPIATPLEPCRVAPYVSLAALRSSSVPSHPAAATSS

NPQAPGVSGTSSSGKVVTVDSPHNHNPAISRSAIKQFSSTVAAAAAFSAFDPAIISVAAHQYAAAITNGTVPAGL
FSVPQYSINLAAFAAAHSKSSSIADLRMKAKKHSESLGLQADMVL

>CG9876_DR

MLNYQQQLHSLPVGNPGNFYYGPTVSGEIYSSHQSHNLESEDKLEDREESGRNLDKIHRFSVDNIMEMKHDAYSK
GKMAMELSSNFGPTGAGCGGADRPAPCSGNLPAGGGHHSRKPRRNRTTFSSAQLTALEKVFERTHYPDAFVREEL
ATKVHLSEARVQVWFQNRRAKFRRNERSVGSRTLLDTAPQLVPAPISNNMHKYANIPHPHPQPPPPPGAYALNFG
PLELRSCQNYTNCYGGFGSSGASGSGVCSFFGATNYCVAANYAKNAYPPL

>crx_DA

MMSYIKQPHYAVNGLTLSASGMDLLHTAVGYPATPRKQRRERTTFTRTQLDILEALFTKTRYPDIFMREEVALKI
NLPESRVQVWFKNRRAKCRQQQQTSGQPKPRPPKKKSSPPPDLTSDPGTSSSVVAAPTPTVPPSVSAGTAPVSV
WSPTSLSPLPDPLCGSGTPCVQRPAPYPMSYGQPSSYSQGYGSSPYFSGLDCSPYLSPMTTQLSASGGALSPLTV
PSMGGSLSQSPSLSSQGYSTASLGFSSVDCLDYKDQQAWKLNFSTVDCLDHKFQVL

>CRX_HU

MMAYMNPGPHYSVNALALSGPSVDLMHQAVPYPSAPRKQRRERTTFTRSQLEELEALFAKTQYPDVYAREEVALK
INLPESRVQVWFKNRRAKCRQQRQQQKQQQQPPGGQAKARPAKRKAGTSPRPSTDVCPDPLGISDSYSPPLPGPS
GSPTTAVATVSIWSPASESPLPEAQRAGLVASGPSLTSAPYAMTYAPASAFCSSPSAYGSPSSYFSGLDPYLSPM
VPQLGGPALSPLSGPSVGPSLAQSPTSLSGQSYGAYSPVDSLEFKDPTGTWKFTYNPMDPLDYKDQSAWKFQIL

>CRX_MO

MMAYMNPGPHYSVNALALSGPNVDLMHQAVPYSSAPRKQRRERTTFTRSQLEELEALFAKTQYPDVYAREEVALK
INLPESRVQVWFKNRRAKCRQQRQQQKQQQQPPGAQTKARPAKRKAGTSPRPSTDVCTDPLGISDSYSPSLPGPS
GSPTTAVATVSIWSPASEAPLPEAQRAGLVASGPSLTSAPYAMTYAPASAFCSSPSAYASPSSYFSGLDPYLSPM
VPQLGGPALSPLSGPSVGPSLAQSPTSLSGQSYSTYSPVDSLEFKDPTGTWKFTYNPMDPLDYKDQSAWKFQIL

>DMBX1_HU

MQHYGVNGYSLHAMNSLSAMYNLHQQAAQQAQHAPDYRPSVHALTLAERLAGCTFQDIILEARYGSQHRKQRRSR
TAFTAQQLEALEKTFQKTHYPDVVMRERLAMCTNLPEARVQVWFKNRRAKFRKKQRSLQKEQLQKQKEAEGSHGE
GKAEAPTPDTQLDTEQPPRLPGSDPPAELHLSLSEQSASESAPEDQPDREEDPRAGAEDPKAEKSPGADSKGLGC
KRGSPKADSPGSLTITPVAPGGGLLGPSHSYSSSPLSLFRLQEQFRQHMAATNNLVHYSSFEVGGPAPAAAAAAA
AVPYLGVNMAPLGSLHCQSYYQSLSAAAAAHQGVWGSPLLPAPPAGLAPASATLNSKTTSIENLRLRAKQHAASL
GLDTLPN

>DMBX1_MO

MQHYGVNGYSLHAMNSLSAMYNLHQQAAQQAQHAPDYRPSVHALTLAERLAGCTFQDIILEARYGSQHRKQRRSR
TAFTAQQLEALEKTFQKTHYPDVVMRERLAMCTNLPEARVQVWFKNRRAKFRKKQRSLQKEQLQKQKEAEGSHGE
GKVEAPASDTQLETEQPPGLPSGDPPAELQLSLSEQSASESAPEDQLDREEDSRAEEPKAEKSPGSESKVPGCKR
GSPKADSPGSLAITPAAPGGGLLGPSHSYSSSPLSLFRLQEQFRQHMAATNNLMHYSSFEVGGPAPAAAAAAAAA
VPYLGVNMAPLSSLHCQSYYQSLSAAAAAHQGVWGSPLLPAPPTGLAPASAALNSKTTSIENLRLRAKQHAASLG
LDTLPN

>DMBX1a_DA

MQHYGVNGYSLHAMNSLSAMYNLHQQAAQQAQHAPDYRPSVHALTLAERLADIILEARYGSQHRKQRRSRTAFTA
QQLEALEKTFQKTHYPDVVMRERLAMCTNLPEARVQVWFKNRRAKFRKKQRSLQKEQLQKQKDVSTDGALAASDK
DEAPSTLNLENQPPSSTSSSSSMEAEAAPHALGSELSVELNVTSAEQSGSESATEDNATDKEEEIKQHREDLKVE
KEPAPGNLSPLCKRLSPKPDSPLGSPAISSSSSGVTGGISQSHSYSSSPLSLFRLQEQFRQHMAATNNLVHYPSF
DMATPSSIPYLGMNVNMPAPLGSLPCQSYYQSLSHHAQQVWNSPLLQASGGLPSHNSKTTSIENLRLRAKQHAAS
LGLDTLPN

>DRGX_DA

MFYFHCPPQLEGECCRTNTLNTNGFGNHASGDFDDGFLRRKQRRNRTTFTLQQLEALEAVFAQTHYPDVFTREEL
AMKINLTEARVQVWFQNRRAKWRKTERGTSEQDGGKEQMNEGNPPARNLNQSPVDHSRSKKEPMELQQNINRVVG

SGGPFFPSCLPGTLLNTATYAQALSQVATLKGSPLCSCCVPDPMGLSFLPPYGCQSNRTASVAALRMKAREHSEA
VLQSANLLGTGSTTGPAAGPALPLSQNTGGDSSPNSSTSKITSLRRTPDKHLHCQKEGPDKK

>DRGX_DR

MFCYQCPPALHPCGPHPPRLPTLDYPFAATHPYTSYSYHPAIHDETFVRRKQRRNRTTFTLQQLEELETAFAQTH
YPDVFTREDLAMKINLTEARVQVWFQNRRAKWRKAERLKDEQRKRENGESSSSLDKLHDSRESSPDITGEIDDDM
DDLPPRQRSHSPLANGQMEQQHSHSHSHSHSRSPGGGMHLDSSDNERPLSSNQLTATPHSASQSLGSISAGSPSP
SGMHREREHTPLVGGGGQGPSSPSNSRNTDSPIEVGGPMSLTTGSRMAASSNNSASSTPTPTTPHAPQMPHSSAA
AAAAFGSHIFGNFGGGSNASDSNCGFRPVLSEQSAVAAAAAAAAAQRSANHPPLFLPPHLAAQFTHQPLFPGLKG
VSPFQSLCSCCSLKPPPPPGSSVVAPLSIPVSSSSAASSPESPKSSGQGSVHDPRSNSVAELRRKAQEHSAALLQ
SLHAAAAAGLAFPGLHLPPLSFAHHPALGQHVVNHNNNNTMRMKHEAQDMTMNGLGPGSGSGSGSGSAGGGTSSA
ALLDLAESAVAYQQQQHATLSPPTTPTQQSSGGVAATEGSPGSGAIAGSGSLNGNVVLTKME

>DRGX_HU

MFYFHCPPQLEGTATFGNHSSGDFDDGFLRRKQRRNRTTFTLQQLEALEAVFAQTHYPDVFTREELAMKINLTEA
RVQVWFQNRRAKWRKTERGASDQEPGAKEPMAEVTPPPVRNINSPPPGDQARSKKEALEAQQSLGRTVGPAGPFF
PSCLPGTLLNTATYAQALSHVASLKGGPLCSCCVPDPMGLSFLPTYGCQSNRTASVATLRMKAREHSEAVLQSAN
LLPSTSSSPGPVAKPAPPDGSQEKTSPTKEQSEAEKSV

>DRGX_MO

MFYFHCPPQLEGTAPFGNHSTGDFDDGFLRRKQRRNRTTFTLQQLEALEAVFAQTHYPDVFTREELAMKINLTEA
RVQVWFQNRRAKWRKTERGASDQEPGAKEPMAEVTPPPVRNINSPPPGDQTRSKKEALEAQQSLGRTVGPTGPFF
PSCLPGTLLNTATYAQALSHVASLKGGPLCSCCVPDPMGLSFLPTYGCQSNRTASVAALRMKAREHSEAVLQSAN
LLPSTSSSPGPASKQAPPEGSQDKTSPTKEQSEGEKSV

>DU4L2_HU

MALPTPSDSTLPAEARGRGRRRRLVWTPSQSEALRACFERNPYPGIATRERLAQAIGIPEPRVQIWFQNERSRQL
RQHRRESRPWPGRRGPPEGRRKRTAVTGSQTALLLRAFEKDRFPGIAAREELARETGLPESRIQIWFQNRRARHP
GQGGRAPAQAGGLCSAAPGGGHPAPSWVAFAHTGAWGTGLPAPHVPCAPGALPQGAFVSQAARAAPALQPSQAAP
AEGVSQPAPARGDFAYAAPAPPDGALSHPQAPRWPPHPGKSREDRDPQRDGLPGPCAVAQPGPAQAGPQGQGVLA
PPTSQGSPWWGWGRGPQVAGAAWEPQAGAAPPPQPAPPDASASARQGQMQGIPAPSQALQEPAPWSALPCGLLLD
ELLASPEFLQQAQPLLETEAPGELEASEEAASLEAPLSEEEYRALLEEL

>DUX4_HU

MALPTPSDSTLPAEARGRGRRRRLVWTPSQSEALRACFERNPYPGIATRERLAQAIGIPEPRVQIWFQNERSRQL
RQHRRESRPWPGRRGPPEGRRKRTAVTGSQTALLLRAFEKDRFPGIAAREELARETGLPESRIQIWFQNRRARHP
GQGGRAPAQAGGLCSAAPGGGHPAPSWVAFAHTGAWGTGLPAPHVPCAPGALPQGAFVSQAARAAPALQPSQAAP
AEGISQPAPARGDFAYAAPAPPDGALSHPQAPRWPPHPGKSREDRDPQRDGLPGPCAVAQPGPAQAGPQGQGVLA
PPTSQGSPWWGWGRGPQVAGAAWEPQAGAAPPPQPAPPDASASARQGQMQGIPAPSQALQEPAPWSALPCGLLLD
ELLASPEFLQQAQPLLETEAPGELEASEEAASLEAPLSEEEYRALLEEL

>DUXA_HU

MAEDTYSHKMVKTNHRRCRTKFTEEQLKILINTFNQKPYPGYATKQKLALEINTEESRIQIWFQNRRARHGFQKR
PEAETLESSQSQGQDQPGVEFQSREARRCRTTYSASQLHTLIKAFMKNPYPGIDSREELAKEIGVPESRVQIWFQ
NRRSRLLLQRKREPVASLEQEEQGKIPEGLQGAEDTQNGTNFTSDSHFSGARTW

>ey_DR

MRNLPCLGTAGGSGLGGIAGKPSPTMEAVEASTASHRHSTSSYFATTYYHLTDDECHSGVNQLGGVFVGGRPLPD
STRQKIVELAHSGARPCDISRILQVSNGCVSKILGRYYETGSIRPRAIGGSKPRVATAEVVSKISQYKRECPSIF
AWEIRDRLLQENVCTNDNIPSVSSINRVLRNLAAQKEQQSTGSGSSSTSAGNSISAKVSVSIGGNVSNVASGSRG
TLSSSTDLMQTATPLNSSESGGASNSGEGSEQEAIYEKLRLLNTQHAAGPGPLEPARAAPLVGQSPNHLGTRSSH
PQLVHGNHQALQQHQQQSWPPRHYSGSWYPTSLSEIPISSAPNIASVTAYASGPSLAHSLSPPNDIESLASIGHQ
RNCPVATEDIHLKKELDGHQSDETGSGEGENSNGGASNIGNTEDDQARLILKRKLQRNRTSFTNDQIDSLEKEFE
RTHYPDVFARERLAGKIGLPEARIQVWFSNRRAKWRREEKLRNQRRTPNSTGASATSSSTSATASLTDSPNSLSA
CSSLLSGSAGGPSVSTINGLSSPSTLSTNVNAPTLGAGIDSSESPTPIPHIRPSCTSDNDNGRQSEDCRRVCSPC

PLGVGGHQNTHHIQSNGHAQGHALVPAISPRLNFNSGSFGAMYSNMHHTALSMSDSYGAVTPIPSFNHSAVGPLA
PPSPIPQQGDLTPSSLYPCHMTLRPPPMAPAHHHIVPGDGGRPAGVGLGSGQSANLGASCSGSGYEVLSAYALPP
PPMASSSAADSSFSAASSASANVTPHHTIAQESCPSPCSSASHFGVAHSSGFSSDPISPAVSSYAHMSYNYASSA
NTMTPSSASGTSAHVAPGKQQFFASCFYSPWV

>eyg_DR

MSQHRLLEFSRFGGLRGYDIAQHMLTQQGAVSKLLGSLRPPGLIGGSKPKVATPTVVSKIEQYKRENPTIFAWEI
RERLISEGVCTNATAPSVSSINRILRNRAAERVATEFARTAAYGLYPPPPHPYGSFTWHPAGNVPGGQGVPPPPP
PSALWSVAAPTLANLPPSAASAVPVSTCGSLSSAHLMAGGAGGTPTNRAISPGSGSHDTLESADENRHIDSDYLD
DDDEPKFRRNRTTFSPEQLEELEKEFDKSHYPCVSTRERLSSRTSLSEARVQVWFSNRRAKWRRHQRMNLLKRQR
SSPANPLHSQQSNDAPASSPTPSNHSSASTSAPVAPVPPPQQPLPLCGDHSPQGPPPSVLLHPLHGPPGGHHHHH
PLHLPTHPAALQLLSHYHQQQQQQQQQHQQQQQQHHQQQQQLSPSGCNPAANHLSMGGERSAFRSLVSSPSAPA
FLGLARQYAVAASLTVAEEQRSRLHYSAGGLGSGGEGTGPNTNTTQSGGSTMTVDNSSSDSDEEINVHDDSRW

>gsb_DR

MAVSALNMTPYFGGYPFQGQGRVNQLGGVFINGRPLPNHIRRQIVEMAAAGVRPCVISRQLRVSHGCVSKILNRF
QETGSIRPGVIGGSKPRVATPDIESRIEELKQSQPGIFSWEIRAKLIEAGVCDKQNAPSVSSISRLLRGSSGSGT
SHSIDGILGGGAGSVGSEDESEDDAEPSVQLKRKQRRSRTTFSNDQIDALERIFARTQYPDVYTREELAQSTGLT
EARVQVWFSNRRARLRKQLNTQQVPSFAPTSTSFGATPTTSAAPAPNMGMSLYSSQSWPSSGAYENHAAYGGSVA
SMSPASSTSGTSSAAHSPVQTQAQQPGTGSEFMTSTYGVGSSNATYPSAAYSMPQTPATSAEQLRSQFASAAASG
SHHPSTWDSYNFAGSFFPPASAAGNHISGYHHQVDQKSSMMTTAPTYPYFGF

>GSC_DA

MPAGMFSIDSILAGRPSCKDSVLLHRNAPVVFSNLTESLYTAAGDFNGLYSHTGPPAPNLQSVNGRIGYNNYYYG
QLHVQGPTGPACCGAIPTLGSQQCPCIPTGYDSAGSVLISPVPHQMMSYMNVGTLSRTELQLLNQLHCRRKRRHR
TIFTDEQLEALENLFQETKYPDVGTREQLARKVHLREEKVEVWFKNRRAKWRRQKRSSSEESENSQKWNKSTKTT
SEKIEEGKSDVDSDS

>GSC_DR

MLAQMVETNSPPAGYTLKRSPSDLGEQQQPPRQISRSPGNTAAYHLTTAMLLNSQQCGYLGQRLQSVLQQQHAQH
QQSQSQTPSSDDGSQSGVTILEEERRGGAAAASLFTIDSILGSRQQGGGTAPSQGSHISSNGNQNGLTSNGISLG
LKRSGAESPASPNSNSSSSAAASPIRPQRVPAMLQHPGLHLGHLAAAAASGFAASPSDFLVAYPNFYPNYMHAAA
VAHVAAAQMQAHVSGAAAGLSGHGHHPHHPHGHPHHPHLGAHHHGQHHLSHLGHGPPPKRKRRHRTIFTEEQLEQ
LEATFDKTHYPDVVLREQLALKVDLKEERVEVWFKNRRAKWRKQKREEQERLRKLQEEQCGSTTNGTTNSSSGTT
SSTGNGSLTVKCPGSDHYSAQLVHIKSDPNGYSDADESSDLEVA

>GSC_HU

MPASMFSIDNILAARPRCKDSVLPVAHSAAAPVVFPALHGDSLYGASGGASSDYGAFYPRPVAPGGAGLPAAVSG
SRLGYNNYFYGQLHVQAAPVGPACCGAVPPLGAQQCSCVPTPPGYEGPGSVLVSPVPHQMLPYMNVGTLSRTELQ
LLNQLHCRRKRRHRTIFTDEQLEALENLFQETKYPDVGTREQLARKVHLREEKVEVWFKNRRAKWRRQKRSSSEE
SENAEKWNKTSSSKASPEKREEEGKSDLDSDS

>GSC_MO

MPASMFSIDNILAARPRCKDAVLPVAPSAAAPVVFPALHGDSLYGAGGGTSSDYGAFYPRPVAPGGAGLPAAVGS
SRLGYNSYFYGQLHVQAAPVGPACCGAVPPLGAQQCSCVPTPPGYEGPGSVLVSPVPHQMLPYMNVGTLSRTELQ
LLNQLHCRRKRRHRTIFTDEQLEALENLFQETKYPDVGTREQLARKVHLREEKVEVWFKNRRAKWRRQKRSSSEE
SENAEKWNKTSSKASPEKREEEGKSDLDSDS

>GSC2_HU

MAAAAGGAASRRGAGRPCPFSIEHILSSLPERSLPARAACPPQPAGRQSPAKPEEPGAPEAAPCACCCCCGPRAA
PCGPPEAAAGLGARLAWPLRLGPAVPLSLGAPAGGSGALPGAVGPGSQRRTRRHRTIFSEEQLQALEALFVQNQY
PDVSTRERLAGRIRLREERVEVWFKNRRAKWRHQKRASASARLLPGVKKSPKGSC

>GSC2_MO

MATAGSAASRRDPGRPCPFSIEHILSSLPERRPATRPPQPVGGRNPAELDEPEAPVPAAPCACCCCCNPRAATRG
TPETAGLRLAWPLRLAPATPSPLTAPRAGSPALTGTSGPGPQRRTRRHRTIFSEEQLQALEALFVQNQYPDVGTR
ERLAVRIRLREERVEVWFKNRRAKWRHQKRASSSRLLPGTKKTPKESC

>HBN_DR

MMTTTTSQHHQHHPIMPPAMRPAPVQESPVSRPRAVYSIDQILGNQHQIKRMYTPSEVLITHPHHGHPHHIHHLH
SSNSNGSNHLSHQQQQQHSQQQHHSQQQQQQQQLQVQAKREDSPTNTDGGLDVDNDDELSSSLNNGHDLSDMERP
RKVRRSRTTFTTFQLHQLERAFEKTQYPDVFTREDLAMRLDLSEARVQVWFQNRRAKWRKREKFMNQDKAGYLLP
EQGLPEFPLGIPLPPHGLPGHPGSMQSEFWPPHFALHQHFNPAAAAAAGLLPQHLMAPHYKLPNFHTLLSQYMGL
SNLNGIFGAGAAAAAAAASAGYPQNLSLHAGLSAMSQVSPPCSNSSPRESPKLVPHPTPPHATPPASGNGGGGLL
TGGLISTAAQSPNSAAGASSNASTPVSVVTKGED

>HESX1_DA

MASLANSPSVFTIDSILGLDRPEQRTCPYRPWTDVKPACQNRRVVTENDAPVDVRGNEDGKSFSKSPTDSYRRTL
NWYIGRRPRTAFSSVQIKILESVFQVNSYPGIDIREELAKKLHLDEDRIQIWFQNRRAKLKRSHRESQFLMVKNV
LSDLQTGREEH

>HESX1_HU

MSPSLQEGAQLGENKPSTCSFSIERILGLDQKKDCVPLMKPHRPWADTCSSSGKDGNLCLHVPNPPSGISFPSVV
DHPMPEERASKYENYFSASERLSLKRELSWYRGRRPRTAFTQNQIEVLENVFRVNCYPGIDIREDLAQKLNLEED
RIQIWFQNRRAKLKRSHRESQFLMAKKNFNTNLLE

>HESX1_MO

MSPSLREGAQLRESKPAPCSFSIESILGLDQKKDCTTSVRPHRPWTDTCGDSEKGGNPPLHAPDLPSETSFPCPV
DHPRPEERAPKYENYFSASETRSLKRELSWYRGRRPRTAFTQNQVEVLENVFRVNCYPGIDIREDLAQKLNLEED
RIQIWFQNRRAKMKRSRRESQFLMAKKPFNPDLLK

>XP_002159617.3_DENV_036790

MLEQWSDDNKMEIFKPQQSGELTCNVDKRSINEDGVGQVICLENPHLLKTLQEHSLAAATVSNLNINIKRFNDAK
ENKNTCRDNHGGINQLGGTFVNGRPLIEPVRRKIVELAHQGVRPCDISRQLRVSHGCVSKILSRFYETGSVRPGV
IGGSKPKVATPSVVAKIQEYKQHNPTMFAWEIRDKLLSEQICDSDSVPSVSSINRIVRNRLGSSSHASMADLNSP
LLKIDPDMTHYFVHPGLPIQIGAISTNFPGSIHAHLPRTTSGSYSISGILGMAVPSSQGNLGTLIMCSDYSIPNV
QQIHSHVLNDEKGNVHIMQEGEIAPSIDHPTQVNANEYVLESSDSFHDSSAASSSSLSNEEDKFSNEQINKPYYM
KMDLNFDSENEKNTNKKTSMQINDVDDSKFDMSLQSTIHHAFSHSNEQTNHTFKKAMRRVRTTFSLEQRRALEDA
FEKTPYPDAEQREEISIQCDLPEPRVQVWFSNKRAKLRRQDRNEDKQDSSKNKEMISSHLDFQRIQNHIKQYDCK
NNSEENNQSCFPDEYQDQINVHDDVKNCLLNSELNTSTTDHRFTNRVLSEIKTSDSATNSCSLPPVSTLTKSTNQ
HHDDGWPHQQRKLFVLGNY

>XP_002160848.1

MRELNNHEYQNQRFSNGKRKQRRYRTTFTQFQLDELERAFDKTHYPDVFMREELAVRVHLTEARVQVWFQNRRAK
WRKREKLSYNVHQQQHGSNGEVSSPYHISTSPKIQSQQRPVSSQMPTPLSHHTAFQPWAQSPYCPQSTSPYPQYP
HYQQSSMIGNYARPSSYQYMSHHTGSVISSTPENLSPQDSQFTGHSSFNNLKGQNQLIFA

>XP_002161581.1

MARNYSSEFSNMGWSSPYNGVPSYSSMPSGFPYHSNVNMHYPLAPSCGFPRKQRRERTTFTKSQLEILEELFLKT
HYPDIFMREEAARKINLPESRVQVWFKNRRAKHRQKAKQDKTQKKTSNSISSENSDIKPDTSPAPSLSPKTSVPS
PESPCYKSNNNASTSSTSSPINSIWRPAKSTSLYDNTHSRNYPTSPILSPCANNSVPYNPPSYLSQQPYIRSTHH
YPSYVNINSNGPAGSEHSSHSSNGIIDFPPHYQNQMHSSTPWGYATM

>XP_002162903_DENV_003026

MFTWDEKISREYSLAKCCNDHTIVEDDYYAMCRSKQRRNRTTFSKQQLMELELTFQKKHYPDINTREALAEQIGI
TEARIQVWFQNRRAKWRKLSTTKQRQLQREANNRYSSIDTWLEELKSRKHDISKELLTVKKESPPVNDYANNLYK
DKKIKIDNEEKSVEIRTSPFDSRQRSTEQERVRYLHQFPYMYSSPSPPYPSHFSCPCKTCHVASCFPLIKKPSYV
KKELNFLECLDEHTRRGVRIGYKYPEDLHKNFKEPEIKEEKFLPSRW

>XP_002168027.1_DENV_045465

MIVYNQSHNSEMPELQQEDNAAFDQNMNIDRKKHTSYSIRDILGLKEGIKSNGEVSLGNSFVDAIPSTRSPQFPE
SDSVSSPESSRSPTSPSLRVQSLNIGGSISGDDCETLDSPTLDNDGALTSDEVSPTVLDTYRQQALSMPSKKRRY
RTTFTTHQLDELERVFNRTHYPDIFLREEMAVKLGLTEARIQVWFQNRRAKWRKRNKSTSLSSPTSPPPLPQLNN
YLKPYTPTQPSTPFNNSISNSLFLKSLNHPSQTEKFNRFNGSLSTKLEPWYENNIRYIHRNIYNYDSRSINNGYR
LIDKDQPLSWWPVHGQPV

>XP_004208605.2_DENV_021903

MESQSSKLSFSIERILTTPSTKKHEITRTTHGIFLLKPCEMFFYPSDAVQRPIISCSKNERYKLPYRKKEIKFCK
MMSKKVFMDEKDRFSLSQKAILRQHPTPEHPTPEKIKGQPILCQTGYLRSSLEGSNISYCKVTSNSDDVSNLIEN
NKRNRTIFSKKQSCVLEDVFKKTHYPDLKCRYEISQQTKLAENRIQVWFQNRRAKWRRTEKVWGEGSIMAKYGLY
GAMVRHSLK

>XP_012556935.1_DENV_099801

MMPVSSCKLKYCPCRLPDSSSLPDRKYVENSFSYKFGMETLHLQKVPSYRTVSYRNFNELNSKFINQYSSLKYSI
DDMQERHFLQDKQLRCTSLQDNQSRCTSLQNNQLYCTSLQNKQLYCKKLDTLNSYYCTSQNGYRRIRMRTNFSPW
QLEELETAFKKSYYPDVYTREVLALKLEIAESRIQVWFQNRRAKWRKAERNLLINNNIDNTQVESKSKTENLSLK
HKIPNAAKEELSTVAITDNSAASIDENKTTNLRQDKSVQEWHVKLTYKNKTEHSDDFCKWQQNVFEKNDDFNRVP
PKLIPISKNFGKSKLD

>XP_012557153.1

MFTWDEKISPKCCNDHTIVEDDYYAMCRSKQRRNRTTFSKQQLMELELTFQKKHYPDINTREALAEQIGITEARI
QVWFQNRRAKWRKLSTTKQRQLQREANNRYSSIDTWLEELKSRKHDISKELLTVKKESPPVNDYANNLYKDKKIK
IDNEEKSVEIRTSPFDSRQRSTEQERVRYLHQFPYMYSSPSPPYPSHFSCPCKTCHVASCFPLIKKPSYVKKELN
FLECLDEHTRRGVRIGYKYPEDLHKNFKEPEIKEEKFLPSRW

>XP_012558124.1_DENV_044489

MIHKPMAKMQFSIDMILGNSKSEEKQQNDVKLQREFNQEKGINVSSTIRFNSPISSEIKNDEIEGENRQKLRRNR
TTFTTYQLHQLERSFDKTQYPDVFTRENLALKLDLSEARVQVWFQNRRAKWRKREKLSFGNSPSNSVCSSAIEYH
QDSQISIPEVPILEVTPIVKRSFIANKQSCSCSHITDNKHLGYDNSSLIKPTLPHTCTARSCLYYGGKRHKNRFS
DLRCSHEENFNKRTFHKIIYQEPCNRRYTDLSDHTDHSLRYSYFH

>XP_012559896.1_DENV_052386

MSWSYSPTPPFPLSHHVSNNYSAYFPYNYNGFPRKQRRERTTFTKFQLEILDNLFKETKYPDVFLREDVARRISL
PESRVQVWFKNRRAKHRQKSKKKPFQSDKDCDLEKLCSSTVVNNKQSRNTVSILPAKSVEAVFKANNSESQSSFD
VNQQAIRQNSSFNSSNQQISSAALEKSQTFPCYLPTSLSSYRPFLIEAHSSTYSSAQARSFYSNYAQAPVEYQSC
VVNFNNSQAVSTQWTNYGSSI

>XP_012566347.1

MSRETNLSFSVENILKHKHKKDKCLIYHESNKKNIEVEHFERNSLWLINPTNLSFSINDYSKQRISPDGRLLDKL
NEKQDHLYGRKHRRVRTAFTHHQLTTLERTFETSHYPDVVLRERLASFTGLAESRIQVWFKNRRAKYRKHQLYDS
STTDLNDDTAYFTDNKKSHKKENISRTAKPKHIHHLYSEKTTSLPNLPGYNNLTNLKSVIKSPTIYGTLISF

>DENV_011065_XP_002164986.2

MLTNDKPASSQASNVLKKTESPVQARVKQDVENKEDTSGYTAGVDNRIRRQRTHFTVTQLHRLETCFARNRYPDM
AMREDIAQWCSLTESRVRIWFKNRRAKWRKKERHLERPDICNFAPTYRFESPSPYERRPTFPTTYSSNVSQPTLP
ICSSLNNRSSHASLGHYYSHNTHWYPSDHPVHSLSQRNSPSNSPAGWYGINYNEPVRGYSTCPQSSSSTLNREDE
MMSPISFSQRTHTFSPGDHSYQHSI

>DENV_022238_XP_012562202.1

MSRETNLSFSVENILKHKHKKDKCLIYHESNKKTIEVEHFERNSLWLINPTNLSFSINDFSKQRISPDGRLLDKL
NEKQDHLYGRKHRRVRTAFTHHQLTTLERTFETSHYPDVVLRERLASFTGLAESRIQVWFKNRRAKYRKHQLYDS
STTDLNDDTTYFTDNKKSHKKENISRTAKPKHIHHLYSEKTTSLPNLPGYNNLTNLKSVIKSPTIYGTLISF

>DENV_024188_XP_012566844.1

MHSASVAIMDSKRKKLSTFSVKDILGLDEADTRKETKNNYEEDINEQGKKTRQCFSTTQVEQLEKLFNEINYPDA
YTRQMLAKKMKVSETRIQIWCQNRRAKIRRQRKLHQPLIPQSTDIHNINKFQNFPWHHYLQQTYLPFHHNTELAC
KSCSSDLPLYERRPELLFRNFGTNFTGHKKYFH

>DENV_026119_XP_002164410.3

MSELFCSSNHIDFSGNYIPSYFEETISPSSLLSDFLKELDSTNQLSNREHSHTNFSTLGKSTFNTSKKATKKTKN
KTIDVANSCNATNSCIIVNNNVTNSSINFKNNCSVVNATKSINCSKTVNSILSSVSSNGHSSNVNLIFGSTSNTN
EKKLLIEEGSKDNEKKMKNKDDNSVFNKNSFGDSNEEENEENDHSKQKRHRTRFTQTQLNELERYFNKTHYPDIF
VREELAMRIDLTESRVQVWFQNRRAKWKKRRKSVNMFDNTSNVFSSYLSHSLTENNQLEQPYYENDRTWPSQSPH
FQIDSPDDRQHVFHQQDVNGFTYNQLSSNYGTERNISYMLPQYYIGDNMTSMEFNTPEQSNIILEPNFTNCIGID
RFDRSVDRLCRKEDDQKLLYLERQNLF

>DENV_027852_XP_004207550.1

MRGFTIDEILRSSNDIESKTPGVFYHVERTEASNELKFCRESPNSFCSLSSIEMEEDKCGRRPRTSITLCQRDFL
ECEFQKERYPTLLYINKLSSKINLSQYVIKVWFQNRRAKYRKEGHKRKPTINIDYTDSAKNVRISSTNCVRKLRQ
NFSASNSKILRRHSTSYDLYGPRSLKCDVWFQKDRKCCSSAADKSKCCYFPT

>DENV_043831_XP_002165604.2

MPYQWLTCLLGRCNGTFEKKFEMSSFLMENILKEEFNTKKFSLKNLQQNQPRLEFQCISHNTSPEFKTKTQERYE
RNILLDEYSCFSHSCHLCYGQLNSDPFKLSSKISSELRRQSSQDYPDRNYMERSCFYCPTQHFILNNTNLNTDKP
FKKNSYSSHFLKRRHRTIFSDEQLNVLERLFNKTHYPDVIVREEIAGIINLTEEKVEVWFKNRRARWRKQKKDLS
GIY

>DEN_079506_XP_002157088.1

MTMSDSPLDSPLNMNSGAIKSETPPKRRRERTTFTKAQLDVLEDMFGKTMYPDVFMREEVAKKINLAEARVQVWF
KNRRAKFRRSRENPGRHFDNNKYLGREKLMPEDLIRTFPKSSGGTPHSYPNTSNYPPLWKYPGADHSPPYAGNNI
IEEPVYRQSNPYAPISPYEYNFPSGRPPYFTQPPYYSPSAPSQDYLSNSVSAPSSTNSISSLSFSQHDTKNELNL
PASYWSGAGYMGANM

>DENV_110060_XP_002163599.1

MRKMPRLSPDNKDFSDSANEEDSFKQRSFKVKNLTESEDESDGCLKQKRHRTRFNPSQLNELERYFNKTHYPDIF
VREELALRIGLTESRVQVWFQNRRAKWKKRKKSFGSVQNCELFQPYYARSTTPYNIPTLEQVWSPDLEEVQINQS
NYSDTQCYINKNNSLSNTVLNELYHETRYAGRHDLGDWSQLRNTFNDAVPENNYIFR

>DENV_130791_XP_012554469.1

MARNYSSEFSNMGWSSPYNGVPSYSSMPSGFPYHTNVNMHYPLAPSCGFPRKQRRERTTFTKSQLEILEELFLKT
HYPDIFMREEAARKINLPESRVQVWFKNRRAKHRQKAKQDKTQKKTSNSISSENSDIKPDTSPAPSLSPKTSVPS
PESPCYKSNNNASTSSTSSPINSIWRPAKSTSLYDNTHSRNYPTSPILSPCANNSVPYNPPSYLSQQPYIRSTHH
YPSYVNINSNGPAGSEHSSHSSNGIIDFPPHYQNQMHSSTPWGYATM

>DENV_177203

MFTWDEKISPKCCNDHTIVEDDYYAMCRSKQRRNRTTFSKQQLMELELTFQKKHYPDINTREALAEQIGITEARI
QVWFQNRRAKWRKLSTTKQRQLQREANNRYSSIDTWLEELKSRKHDISKELLTVKRESPPVNDXKKVLKLEHLLS
IQDRDLPNKSEFATFTNSRTCTLLLRHLTQAISHVHVRRVM

>DENV_189615_XP_002160816.1

MIKKQNFQTVIQVHNSISLGVHNVDAKHDGSLLNSEEYQNQRFSNGKRKQRRYRTTFTQFQLDELERAFDKTHYP
DVFMREELAVRVHLTEARVQVWFQNRRAKWRKREKLSYNVHQQQHGSNGEVSSPYHISTSPKIQSQQRPVSSQMP
TPLSHHTAFQPWAQSPYCPQSTSPYPQYPHYQQSSMIGNYARPSSYQYMSHHTGSVISSTPENLSPQDSQFTGHS
SFNNLKGQNQLIFA

>DENV_191873

MTLLGMLPSSQEENSNFSDFKEIKREEYQNQRFSNGKRKQRRYRTTFTQFQLDELERAFDKTHYPDVFMREELAV
RVHLTEARVQVWFQNRRAKWRKREKLSYNVHQQHGSNGEVSSPYHISTSPKIQSQQRPVSSQMPTPLSHHTAFQ
PWAQSPYCPQSTSPYPQYPHYQQSSMIGNYARPSSYQYMSHHTGSVISSTPENLSPQDSQFTGHSSFNNLKGQNQ
LIFA

>ISX_HU

MCAEVGPALCRGMERNSLGCCEAPKKLSLSFSIEAILKRPARRSDMDRPEGPGEEGPGEAAASGSGLEKPPKDQP
QEGRKSKRRVRTTFTTEQLHELEKIFHFTHYPDVHIRSQLAARINLPEARVQIWFQNQRAKWRKQEKIGNLGAPQ
QLSEASVALPTNLDVAGPTWTSTALRRLAPPTSCCPSAQDQLASAWFPAWITLLPAHPWETQPVPGLPIHQTCIP
VLCILPPPHPKWGSICATST

>ISX_MO

MAGPTIHRDMEKSSGYCEAPENLGLSFSIEAILKKPTERRSLPRPQSICKEDSRQTTIPGSKLERPPQDQPQEEK
KNKRRVRTTFTTEQLQELEKLFHFTHYPDIHVRSQLASRINLPEARVQIWFQNQRAKWRKQEKSGNLSAPQQPGE
AGLALPSNMDVSGPVLTPTAMTTLVPPTECCLLSQTQLPSSWFPTQIPLVPWHPWDLQPLPGPLTQHPCVPTFMF
PPLHPKWGSICATST

>MIXL1_DA

MAVVHGNLGNGHLKQFQLFPNADMERRTGMHTSDYALQDRMKFLDGASRADNVSLFTHRRKRTNFTQQQIDVLEK
VYLDTKYPDIYLREKLEALTGLPESRIQVWFQNRRAKSRRQVGISIPHKTSGNILTPNNLLMHQFTTHQNHSGLE
NLQRTSTFTADSFHQQLINSSEEKISTIKGDIYDPTTTPCMFSRTQENPIKTDHLSVVVPRSYTQQYPKEHEQFT
HSANHAKSTMKQFLVEYDNFPPNKTIGPEMKVVIPPLPSQSNFMMSSSSPKHIACSVQNMSVQTQPELFATFSPI
RASEAVEFSDSDSDWERDAMSGFNGFI

>MIXL1_HU

MATAESRALQFAEGAAFPAYRAPHAGGALLPPPSPAAALLPAPPAGPGPATFAGFLGRDPGPAPPPPASLGSPAP
PKGAAAPSASQRRKRTSFSAEQLQLLELVFRRTRYPDIHLRERLAALTLLPESRIQVWFQNRRAKSRRQSGKSFQ
PLARPEIILNHCAPGTETKCLKPQLPLEVDVNCLPEPNGVGGGISDSSSQGQNFETCSPLSEDIGSKLDSWEEHI
FSAFGNF

>MIXL1_MO

MAAAGSQQLQFAEGAAFPIFPAAHPGGQLLPAMRPASGLPAAPHDSRAPAATQCFPNRDSSPTAQTPAGLDPPGP
SKGSAAPSAPQRRKRTSFSSEQLQLLELVFRQTMYPDIHLRERLAALTLLPESRIQVWFQNRRAKSRRQSGKSFQ
PLSSRRGVFLHCPAPGTEARCLKPQLPLEADVNHVPDPSMTGGGVCTSGSQSFETYSSLSEDIGSKLDSWEEHIF
SALGNF

>MXTX1_DA

MWKDSTIDATAKTSGSGAVSRSASRRKRTSFSKEHVELLRATFETDPYPGISLRESLSQTTGLPESRIQVWFQNR
RARTLKCKGGKKPLWQTDLPAYNNMQTSPMQIHRKNNESSGATGTLCSPPPAYPDRIKEEMEKDAVCGCDTPSSM
SISDDSGYCTPSYHQSRAIQSHMSPSPLLSPEHQMPPNWGVRYGRRSPMSSMWSPYHLEAYACNPGFFYSHSEKH
NTLQPLTPVTPDSGCWEAGLDRTSPVDNPDVPRTEGQLESGVHHGPLPELPTLSLQEILGELQGDWWQGEGLNNH
STEDNHVYG

>MXTX2_DA

MKDMWTDCIAQDGNSQASKIAGRRKRTSFTKEHLELLKMAFNVDPYPGISVRESLSQATGLPESRIQVWFQNKRA
RTLKNRAIQSSPQLDNKSPLPSPFLPPHMASVGVSAQQRGIQESPFNIQMSQTSPQHFTFPPSDYSTPVVKPRQT
RLMGTSSCSPSDLQATPDSWSYAGSTQISPESWDVAAENFGNSYKDESPFFLYPPPPYPYGSTRVGCPSSMESLS
TSPASSDSAFWDMGLENCSPSVPYTSCGSPWDRLTEEQPVAPLPDLSSQCLEDVLGEMEPAWWSFNGQMDL

>NOBOX_HU

MALLLTLTSPDLEGTWDTRDKDGFKAQEGPPLAVPEFPVCGLYRIYGVCGSFSSFFIIRCSLCALETLKSPQHDP
LEIPEQSLKLIPLVSGKRELTRGQKAGEKPLAAGPGEEELLRGSAPHAQDTQSEELPPSCTISGEKKPPAVSGEA
TGADAGRLCPPPRSRAPHKDRTLARSRPQTQGEDCSLPVGEVKIGKRSYSPAPGKQKKPNAMGLAPTSSPGAPNS
ARATHNPVPCGSGRGPCHLANLLSTLAQSNQNRDHKQGPPEVTCQIRKKTRTLYRSDQLEELEKIFQEDHYPDSD

KRREIAQTVGVTPQRIMVKGAGSLVAGWSGGGPTIETLELQSERSAVAWVWFQNRRAKWRKMEKLNGKESKDNPA
APGPASSQCSSAAEILPAVPMEPKPDPFPQESPLDTFPEPPMLLTSDQTLAPTQPSEGAQRVVTPPLFSPPPVRR
ADLPFPLGPVHTPQLMPLLMDVAGSDSSHKDGPCGSWGTSITLPPPCSYLEELEPQDYQQSNQPGPFQFSQAPQP
PLFQSPQPKLPYLPTFPFSMPSSLTLPPPEDSLFMFPCGPSGGTSQGYCPGASSGQILMQPPAGNIGTASWSDPC
LPELPFPGPFCPQALGHPPGGDGYFPDLFPTPCPQALGRQPSSALSWMPEGARPGTGPLLSKAKEEPPAASLDQP
SALEEARGDDKNSHVP

>NOBOX_MO

MEPTEKLCKKMQGQEAGDKPRTAALETEGPLQDSALPIQDDQDKQSSLPRASLGKRPLSKTSEELMDAGTCRVHK
APTAAACGPQSEEEGCSPPERKAESLKPSISAVPGQATAGSLNSHEGDLKKESLEVTCQFRKKTRTLYRSDQLEE
LERIFQEDHYPDSDKRHEISQMVGVTPQRIMVWFQNRRAKWRKVEKLNEKETKNGPAAPSADSSQHRSAPELLDP
MPTDLEPGPVPPENILDVFPEPPMLLTSEQTLTPFQNNEGAERVAVTPPLLSPPPIRRANLPLPLGPVQTPQVLP
PMRDVPGSDSIYKDKAYVSWGTSIASPPTYSNLEDLGSQDYQASSQLGSFQLSQAPHLPLFPSLQSQFPYLPPFP
YPIPSSMPFLPPEDSLFSFPFGFSGDSSQDYCPGPPPGQILLQPPAENMGTGPWSGHCLPEPPFPRPHYPQALGQ
PLGAEGYFPNLLPTPYALTMSKQSSLGLNGLLEGTRVETGSSLSKMSDEQTSSSLEQPALEEVRDKNKNSHAAGA
KE

>oc_DR

MAAGFLKSGDLGPHPHSYGGPHPHHSVPHGPLPPGMPMPSLGPFGLPHGLEAVGFSQGMWGVNTRKQRRERTTFT
RAQLDVLEALFGKTRYPDIFMREEVALKINLPESRVQVWFKNRRAKCRQQLQQQQQSNSLSSSKNASGGGSGNSC
SSSSANSRSNSNNNGSSSNNNTQSSGGNNSNKSSQKQGNSQSSQQGGGSSGGNNSNNNSAAAAASAAAAVAAAQS
IKTHHSSFLSAAAAAASGGTNQSANNNSNNNNQGNSTPNSSSSGGGGGSQAGGHLSAAAAAAALNVTAAHQNSSP
LLPTPATSVSPVSIVCKKEHLSGGYGSSVGGGGGGGGASSGGLNLGVGVGVGVGVGVGVSQDLLRSPYDQLKDAG
GDIGAGVHHHHSIYGSAAGSNPRLLQPGGNITPMDSSSSITTPSPPITPMSPQSAAAAAHAAQSAQSAHHSAAHS
AAYMSNHDSYNFWHNQYQQYPNNYAQAPSYYSQMEYFSNQNQVNYNMGHSGYTASNFGLSPSPSFTGTVSAQAFS
QNSLDYMSPQDKYANMV

>OdsH_DR

MQVSGWSSVLNLSMDAPNPEISPNSVTNSSSVYMIRQMALIQQARVAAAAVAMQQQQQQQQQQQRDLNRELGMDP
HSEQRIKLDSVSPTHNIHAGSSRGIKQDPLSDEGADSNLGQNDCTESSKKRRGRTNFNSWQLRELERVFQGSHYP
DIFMREALATKLDLMEGRIAVWFQNRRAKWRKQEHTKKGPGRPAHNAHPQSCSGDPIPLSELRARELAQRSKRMK
KAIDRQAKKLQDKGLEVDYARLEAEYLAVHQENGVDENNWLDDDGYDDLHIDVVGVEPEYVTGDSLDHSFCSSRT
YQTKSTSSELDSNDMGLQGRVETPPPPQPPMQNKTLYNSPFSIESLLGS

>OTP_DR

MQQQDQKRHRTRFTPAQLNELERCFSKTHYPDIFMREEIAMRIGLTESRVQVWFQNRRAKWKKRKKTTNVFRTPG
ALLPSHGLPPFGANITNIAMGDGLCGTGMFGGDRWSVGVNPMTAGFGQLNQSSPLSSSLNSGLNSGINMGSALGA
GSYQHYGLNALGDSMMYQHSVGGVSCGPSGSPSATTPPNMNSCSSVTPPPLSAQPNSSQNELNGEPMPLHQQQQQ
QTHQHQQQQTHQHHPMAPPTPTQQQQQLPQSMQSPSDGANDTLHSIAALRRRASELNAIPSYLQMAPHNYEHYNS
NSNSVY

>OTP_HU

MLSHADLLDARLGMKDAAELLGHREAVKCRLGVGGSDPGGHPGDLAPNSDPVEGATLLPGEDITTVGSTPASLAV
SAKDPDKQPGPQGGPNPSQAGQQQGQQKQKRHRTRFTPAQLNELERSFAKTHYPDIFMREELALRIGLTESRVQV
WFQNRRAKWKKRKKTTNVFRAPGTLLPTPGLPQFPSAAAAAAAAMGDSLCSFHANDTRWAAAAMPGVSQLPLPPA
LGRQQAMAQSLSQCSLAAGPPPNSMGLSNSLAGSNGAGLQSHLYQPAFPGMVPASLPGPSNVSGSPQLCSSPDSS
DVWRGTSIASLRRKALEHTVSMSFT

>OTP_MO

MLSHADLLDARLGMKDAAELLGHREAVKCRLGVGGSDPGGHPGDLAPNSDPVEGATLLPGEDITTVGSTPASLAV
SAKDPDKQPGPQGGPNPSQAGQQQGQQKQKRHRTRFTPAQLNELERSFAKTHYPDIFMREELALRIGLTESRVQV
WFQNRRAKWKKRKKTTNVFRAPGTLLPTPGLPQFPSAAAAAAAAMGDSLCSFHANDTRWAAAAMPGVSQLPLPPA
LGRQQAMAQSLSQCSLAAGPPPNSMGLSNSLAGSNGAGLQSHLYQPAFPGMVPASLPGPSNVSGSPQLCSSPDSS
DVWRGTSIASLRRKALEHTVSMSFT

>OTPa_DA

MLSHADLLDARLGELSTPNSGLQLYWMKDAAAELLGHREALKCRLGGGGTDPVHPGDLAPVSDAVEGATLLPGDD
INSAGSNPPGVAVNSKDQEKQQQQQQNPTQSSSQQSQKQKRHRTRFTPAQLNELERSFAKTHYPDIFMREELALR
IGLTESRVQVWFQNRRAKWKKRKKTTNVFRAPGTLLPTHHGLPQFPSAAAAAAAMGDSLCSFHANDTRWAAAGMP
GVSQLQLPPALGRQQAMAQSLSQCSLGAGPPPNSMSLSNMSSNGSGLQSHLYQPTFPGMVPASLSGPTNVTGSPQ
LCSSPDTDVWRGTSIASLRRKALEHTVSMSFT

>OTPb_DA

MLSHADLLDTRLGMKDAAAELLVHREALKCRLGGTDSGHPGDLTSATETVEGTTLLPGEEISNGGSNPNGMQQVN
AKDQEKQQQQNSNQTGGQQNQQKQKRHRTRFTPAQLNELERSFAKTHYPDIFMREELALRIGLTESRVQVWFQNR
RAKWKKRKKTTNVFRAPGTLLPTHGLPQFPSAAAAAMGDSLCSFHANDTRWAAAGMPGVSQLQIPPSLGRQQAMA
QSLSQCSLGAGPPPNNMGLSGSLSSNGSGLQSHLYQPTFPGMVPASLSGPTNVTGSPQLCSSPDSDMWRGTSIAS
LRRKALEHTVSMSFT

>OTX1_HU

MMSYLKQPPYGMNGLGLAGPAMDLLHPSVGYPATPRKQRRERTTFTRSQLDVLEALFAKTRYPDIFMREEVALKI
NLPESRVQVWFKNRRAKCRQQQQSGSGTKSRPAKKKSSPVRESSGSESSGQFTPPAVSSSASSSSSASSSSANPA
AAAAAGLGGNPVAAASSLSTPAASSIWSPASISPGSAPASVSVPEPLAAPSNTSCMQRSVAAGAATAAASYPMSY
GQGGSYGQGYPTPSSSYFGGVDCSSYLAPMHSHHHPHQLSPMAPSSMAGHHHHHPHAHHPLSQSSGHHHHHHHHH
HQGYGGSGLAFNSADCLDYKEPGAAAASSAWKLNFNSPDCLDYKDQASWRFQVL

>OTX1_MO

MMSYLKQPPYGMNGLGLAGPAMDLLHPSVGYPATPRKQRRERTTFTRSQLDVLEALFAKTRYPDIFMREEVALKI
NLPESRVQVWFKNRRAKCRQQQQSGNGTKTRPVKKKSSPVRESSGSESSGQFTPPAVSSSASSSSSASSASANPA
AAAAAGLGGNPVAAASSLSTPTASSIWSPASISPGSAPTSVSVPEPLAAPSNASCMQRSVAAGAATAAASYPMSY
GQGGSYGQGYPAPSSSYFGGVDCSSYLAPMHSHHHPHQLSPMAPSSMAGHHHHHPHAHHPLSQSSGHHHHHHHHH
HHQGYGGSGLAFNSADCLDYKEPAAAAASSAWKLNFNSPDCLDYKDQASWRFQVL

>OTX1a_DA

MMSYLKQAPYAMNGLGLGGATMDLLHPSVGYPTNPRKQRRERTTFTRTQLDILESLFAKTRYPDIFMREEVALKI
NLPESRVQVWFKNRRAKCRQQQQQSSTNSKIRPAKKKPSPSRESPGSESSGHFTPPAVSSSSSSSSSSSGSNPSA
LSGVGLISSSSSGTPVPSIWSPAPVSPVPAPPSLPDISPPASASCMQRAMSSGGGTTGVPSYPMPYNQAPSYAQG
YPTSNAASYFGGMDCGSYLAPMTGHAHHHPHAHHSQLSTAAAVSGHHHPHHIGQSSGHHPHHQGYGGTALPFSSS
DCLDYKEQVASSWKLNFSSAAADCLDYKDQAAWRFQVL

>OTX1b_DA

MMSYLKQPPYAMNGLGLSGAAMDLLHPSVGYPATPRKQRRERTTFTRSQLDILEALFAKTRYPDIFMREEVALKI
NLPESRVQVWFKNRRAKCRQQQQSGSSTKTRPAKKKSSPTRESTGSESSGQFTPPAVSSAGSSSSSSSSTNNTGI
CSTSTSISTVSSIWSPAISPGSAPPSVSLPEPVAPSNTSCMQRSVSGTASSTYPMPYNQTTGYSQGYPTPSGSYF
SGVDCGSYLAPMHSHHHPHQLSPMTASSMPTHPHHHISQSSGHHHHHHHQAYSGTGLAFNSSDCLDYKEQTASSW
KLNFNTTDCLDYKDQASWRFQVL

>OTX2_DA

MMSYLKQPPYTVNGLSLTTSGMDLLHPSVGYPATPRKQRRERTTFTRAQLDVLEALFAKTRYPDIFMREEVALKI
NLPESRVQVWFKNRRAKCRQQQQQQQNGGQNKVRPAKKKSSPAREASSESGASGQFTPPSSTSVPAISTTTAPVS
IWSPASISPLSDPLSTSSSCMQRSYPMTYTQASGYSQGYAGSTSYFGGMDCGSYLTPMHHQLTGPGSTLSPMSSN
AVTSHLNQSPASLPTQGYGASGLGFNSTADCLDYKDQASSWKLNFNADCLDYKDQTSSWKFQVL

>OTX2_HU

MMSYLKQPPYAVNGLSLTTSGMDLLHPSVGYPATPRKQRRERTTFTRAQLDVLEALFAKTRYPDIFMREEVALKI
NLPESRVQVWFKNRRAKCRQQQQQQQNGGQNKVRPAKKKTSPAREVSSESGTSGQFTPPSSTSVPTIASSSAPVS
IWSPASISPLSDPLSTSSSCMQRSYPMTYTQASGYSQGYAGSTSYFGGMDCGSYLTPMHHQLPGPGATLSPMGTN
AVTSHLNQSPASLSTQGYGASSLGFNSTTDCLDYKDQTASWKLNFNADCLDYKDQTSSWKFQVL

>OTX2_MO

MMSYLKQPPYAVNGLSLTTSGMDLLHPSVGYPATPRKQRRERTTFTRAQLDVLEALFAKTRYPDIFMREEVALKI
NLPESRVQVWFKNRRAKCRQQQQQQQNGGQNKVRPAKKKSSPAREVSSESGTSGQFTPPSSTSVPTIASSSAPVS
IWSPASISPLSDPLSTSSSCMQRSYPMTYTQASGYSQGYAGSTSYFGGMDCGSYLTPMHHQLPGPGATLSPMGTN
AVTSHLNQSPASLSTQGYGASSLGFNSTTDCLDYKDQTASWKLNFNADCLDYKDQTSSWKFQVL

>OTX5_DA

MMSYMKQPHYSVNGLTLSGTGMDLLHSAVGYPNTPRKQRRERTTFTRAQLDVLEALFSKTRYPDIFMREEVALKI
NLPESRVQVWFKNRRAKCRQQQQQTSGQTKPRPPKKKSSPARDSSASEPSASTSGPYSPPPPPPGTAITPSSSS
ATVSIWSPASISPLPDPLSAPSTACLQRSSYPMTYSQAPAYGQSYAASSSYFTGLDCSSYLSPMHPQLSASGGAL
SPMSGALSQSPASLSSQGYTAASLGFGAVDCLDYKDQTSAWKLNFNAPDCLDYKDQNSWKFQVL

>PAX3_HU

MTTLAGAVPRMMRPGPGQNYPRSGFPLEVSTPLGQGRVNQLGGVFINGRPLPNHIRHKIVEMAHHGIRPCVISRQ
LRVSHGCVSKILCRYQETGSIRPGAIGGSKPKQVTTPDVEKKIEEYKRENPGMFSWEIRDKLLKDAVCDRNTVPS
VSSISRILRSKFGKGEEEEADLERKEAEESEKKAKHSIDGILSERASAPQSDEGSDIDSEPDLPLKRKQRRSRTT
FTAEQLEELERAFERTHYPDIYTREELAQRAKLTEARVQVWFSNRRARWRKQAGANQLMAFNHLIPGGFPPTAMP
TLPTYQLSETSYQPTSIPQAVSDPSSTVHRPQPLPPSTVHQSTIPSNPDSSSAYCLPSTRHGFSSYTDSFVPPSG
PSNPMNPTIGNGLSPQVMGLLTNHGGVPHQPQTDYALSPLTGGLEPTTTVSASCSQRLDHMKSLDSLPTSQSYCP
PTYSTTGYSMDPVTGYQYGQYGQSKPWTF

>PAX3_MO

MTTLAGAVPRMMRPGPGQNYPRSGFPLEVSTPLGQGRVNQLGGVFINGRPLPNHIRHKIVEMAHHGIRPCVISRQ
LRVSHGCVSKILCRYQETGSIRPGAIGGSKPKQVTTPDVEKKIEEYKRENPGMFSWEIRDKLLKDAVCDRNTVPS
VSSISRILRSKFGKGEEEEADLERKEAEESEKKAKHSIDGILSERASAPQSDEGSDIDSEPDLPLKRKQRRSRTT
FTAEQLEELERAFERTHYPDIYTREELAQRAKLTEARVQVWFSNRRARWRKQAGANQLMAFNHLIPGGFPPTAMP
TLPTYQLSETSYQPTSIPQAVSDPSSTVHRPQPLPPSTVHQSTIPSNADSSSAYCLPSTRHGFSSYTDSFVPPSG
PSNPMNPTIGNGLSPQVMGLLTNHGGVPHQPQTDYALSPLTGGLEPTTTVSASCSQRLEHMKNVDSLPTSQPYCP
PTYSTAGYSMDPVTGYQYGQYGQSKPWTF

>PAX3a_DA

MTALAGGISRMMRPTPPQNYPRGGYSLEVSTPLGQGRVNQLGGVFINGRPLPNHIRHKIVEMAHHGIRPCVISRQ
LRVSHGCVSKILCRYQETGSIRPGAIGGSKPKQSTTPDVEKKIEEYKRENPGMFSWEIRDKLLKDGICDRNNVPS
VSSISRMLRCKFGGNGDEDEDDDEVEKREIEENERRAKHSIDGILGDRSSHSDEGSDVDSEPGLPLKRKQRRSRT
TFTAEQLEELERAFERTHYPDIYTREELAQRAKLTEARVQVWFSNRRARWRKQAGANQLMAFNHLIPGGFPPSAM
SSLQPYQLADSPYPPSSISQVSEQPSTVHRPQPLPPTSVHQSGLGSGPGAQEGSSAYCLSSGRHGFSGYSDGYVA
APGHANPVNPSISNGLPSQVMGLLNPGAVPHQPQSDFALSPLTGGLEPSTGMPASCHSSQRLEALPGLPSMPALP
SSQSYCPSSYSSPGYSVDHVASYQYSQYGQSKVPFII

>PAX3b_DA

MTAFPGTIPRMMRPSLSPNYPRSGFPMEVSTPMGQGRVNQLGGVFINGRPLPNHIRHKIVEMAHHGIRPCVISRQ
LRVSHGCVSKILCRYQETGSIRPGAIGGTKSKTTSPDVEKRVEEYKRENPGIFSWEIRDKLLKEGICDRNNVPTV
SSISRIMRGRSGTRADEEEEEEDEEDIERREREENAHRGAHSIEGILADRSSPSDEGSEVESEPDLPLKRKQRRS
RTTFTADQLEELERAFERTHYPDIYTREELAQRAKLTEARVQVWFSNRRARWRKQAGANQLMAFNHLIPGGFSHH
AMSSLSPYQLSESPYPPAALPQDSGTVHRPQPLPHSSHQSSGGAGPEGGASYCLSSRHGYTGYSDAFGPHNTHNN
NAINNGLSSQVMGLITPSGASHPTQAEFSLDSGSSVSSSCSQRMESLSSLPALSNLSNPQSYSSASFYSLEHSGH
YQYSQYGQNSFHYLRPEIA

>PAX4_DA

MRKPPSNEISVYFSGEGSVNQLGGVFLNGRPLPVYKRRLMIELATEGMRPCEISRILKVSNGCVSKILGRFQRTG
AIGPKAVGGSRPRLLTPEVISIIVQHKRQNPTLFAWEIRQKLATERACRGDQVPSVSSINRILKKIHQDEDIAYP
NEKHYHTEQLNVQSHFQQQMNTNLPRTAHRSRTAFTADQSGRLEKEFTCGLYPDLLTREKLAEETNLSQDTIKKV
WFSNRRARMRRERKFEHNDCGIARPGFLGNCRNALISLSSTAGCSLDDYRAHHAPSAFPLAAQRDRNTFPLAHLN
SNAVSSCSFDQTESLMPMCHHGNEELAYRLVSPNIDERILLANGTQWMNHKTLTGQNIPMF

>PAX4_HU

MHQDGISSMNQLGGLFVNGRPLPLDTRQQIVRLAVSGMRPCDISRILKVSNGCVSKILGRYYRTGVLEPKGIGGS
KPRLATPPVVARIAQLKGECPALFAWEIQRQLCAEGLCTQDKTPSVSSINRVLRALQEDQGLPCTRLRSPAVLAP
AVLTPHSGSETPRGTHPGTGHRNRTIFSPSQAEALEKEFQRGQYPDSVARGKLATATSLPEDTVRVWFSNRRAKW
RRQEKLKWEMQLPGASQGLTVPRVAPGIISAQQSPGSVPTAALPALEPLGPSCYQLCWATAPERCLSDTPPKACL
KPCWDCGSFLLPVIAPSCVDVAWPCLDASLAHHLIGGAGKATPTHFSHWP

>PAX4_MO

MQQDGLSSVNQLGGLFVNGRPLPLDTRQQIVQLAIRGMRPCDISRSLKVSNGCVSKILGRYYRTGVLEPKCIGGS
KPRLATPAVVARIAQLKDEYPALFAWEIQHQLCTEGLCTQDKAPSVSSINRVLRALQEDQSLHWTQLRSPAVLAP
VLPSPHSNCGAPRGPHPGTSHRNRTIFSPGQAEALEKEFQRGQYPDSVARGKLAAATSLPEDTVRVWFSNRRAKW
RRQEKLKWEAQLPGASQDLTVPKNSPGIISAQQSPGSVPSAALPVLEPLSPSFCQLCCGTAPGRCSSDTSSQAYL
QPYWDCQSLLPVASSSYVEFAWPCLTTHPVHHLIGGPGQVPSTHCSNWP

>PAX6_HU

MQNSHSGVNQLGGVFVNGRPLPDSTRQKIVELAHSGARPCDISRILQVSNGCVSKILGRYYETGSIRPRAIGGSK
PRVATPEVVSKIAQYKRECPSIFAWEIRDRLLSEGVCTNDNIPSVSSINRVLRNLASEKQQMGADGMYDKLRMLN
GQTGSWGTRPGWYPGTSVPGQPTQDGCQQQEGGGENTNSISSNGEDSDEAQMRLQLKRKLQRNRTSFTQEQIEAL
EKEFERTHYPDVFARERLAAKIDLPEARIQVWFSNRRAKWRREEKLRNQRRQASNTPSHIPISSSFSTSVYQPIP
QPTTPVSSFTSGSMLGRTDTALTNTYSALPPMPSFTMANNLPMQPPVPSQTSSYSCMLPTSPSVNGRSYDTYTPP
HMQTHMNSQPMGTSGTTSTGLISPGVSVPVQVPGSEPDMSQYWPRLQ

>PAX6_MO

MQNSHSGVNQLGGVFVNGRPLPDSTRQKIVELAHSGARPCDISRILQVSNGCVSKILGRYYETGSIRPRAIGGSK
PRVATPEVVSKIAQYKRECPSIFAWEIRDRLLSEGVCTNDNIPSVSSINRVLRNLASEKQQMGADGMYDKLRMLN
GQTGSWGTRPGWYPGTSVPGQPTQDGCQQQEGGGENTNSISSNGEDSDEAQMRLQLKRKLQRNRTSFTQEQIEAL
EKEFERTHYPDVFARERLAAKIDLPEARIQVWFSNRRAKWRREEKLRNQRRQASNTPSHIPISSSFSTSVYQPIP
QPTTPVSSFTSGSMLGRTDTALTNTYSALPPMPSFTMANNLPMQPPVPSQTSSYSCMLPTSPSVNGRSYDTYTPP
HMQTHMNSQPMGTSGTTSTGLISPGVSVPVQVPGSEPDMSQYWPRLQ

>PAX6a_DA

MMQNKVYDICNEGHSGVNQLGGVFVNGRPLPDSTRQKIVELAHSGARPCDISRILQTHADAKVQVLDNENVSNGC
VSKILGRYYETGSIRPRAIGGSKPRVATPEVVGKIAQYKRECPSIFAWEIRDRLLSEGVCTNDNIPSVSSINRVL
RNLASEKQQMGADGMYEKLRMLNGQTGTWGTRPGWYPGTSVPGQPNQDGCQQSDGGGENTNSISSNGEDSDETQM
RLQLKRKLQRNRTSFTQEQIEALEKEFERTHYPDVFARERLAAKIDLPEARIQVWFSNRRAKWRREEKLRNQRRQ
ASNSSSHIPISSSFSTSVYQPIPQPTTPVSFTSGSMLGRSDTALTNTYSALPPMPSFTMANNLPMQPSQTSSYSC
MLPTSPSVNGRSYDTYTPPHMQAHMNSQSMAASGTTSTGLISPGVSVPVQVPGSEPDMSQYWPRLQ

>PAX7_HU

MAALPGTVPRMMRPAPGQNYPRTGFPLEVSTPLGQGRVNQLGGVFINGRPLPNHIRHKIVEMAHHGIRPCVISRQ
LRVSHGCVSKILCRYQETGSIRPGAIGGSKPRQVATPDVEKKIEEYKRENPGMFSWEIRDRLLKDGHCDRSTVPS
GLVSSISRVLRIKFGKKEEEDEADKKEDDGEKKAKHSIDGILGDKGNRLDEGSDVESEPDLPLKRKQRRSRTTFT
AEQLEELEKAFERTHYPDIYTREELAQRTKLTEARVQVWFSNRRARWRKQAGANQLAAFNHLLPGGFPPTGMPTL
PPYQLPDSTYPTTTISQDGGSTVHRPQPLPPSTMHQGGLAAAAAAADTSSAYGARHSFSSYSDSFMNPAAPSNHM
NPVSNGLSPQVMSILGNPSAVPPQPQADFSISPLHGGLDSATSISASCSQRADSIKPGDSLPTSQAYCPPTYSTT
GYSVDPVAGYQYGQYGQTAVDYLAKNVSLSTQRRMKLGEHSAVLGLLPVETGQAY

>PAX7_MO

MAALPGAVPRMMRPGPGQNYPRTGFPLEVSTPLGQGRVNQLGGVFINGRPLPNHIRHKIVEMAHHGIRPCVISRQ
LRVSHGCVSKILCRYQETGSIRPGAIGGSKPRQVATPDVEKKIEEYKRENPGMFSWEIRDRLLKDGHCDRSTVPS
VSSISRVLRIKFGKKEDDEEGDKKEEDGEKKAKHSIDGILGDKGNRLDEGSDVESEPDLPLKRKQRRSRTTFTAE
QLEELEKAFERTHYPDIYTREELAQRTKLTEARVQVWFSNRRARWRKQAGANQLAAFNHLLPGGFPPTGMPTLPP
YQLPDSTYPTTTISQDGGSTVHRPQPLPPSTMHQGGLAAAAAAADTSSAYGARHSFSSYSDSFMNPGAPSNHMNP

VSNGLSPQVMSILSNPSAVPPQPQADFSISPLHGGLDSASSISASCSQRADSIKPGDSLPTSQSYCPPTYSTTGY
SVDPVAGYQYSQYGQTAVDYLAKNVSLSTQRRMKLGEHSAVLGLLPVETGQAY

>PAX7a_DA

MATLPGTVPRMMRPAPGQNYPRTGFPLEVSTPLGQGRVNQLGGVFINGRPLPNHIRHKIVEMAHHGIRPCVISRQ
LRVSHGCVSKILCRYQETGSIRPGAIGGSKPRQVATPDVEKRIEEYKRENPGMFSWEIRDKLLKDGVCDRGTVPS
GEASSVSSISRVLRARFGKKDDDDECDKKDEDGEKKTKHSIDGILGDKGNRTDEGSDVESEPDLPLKRKQRRSRT
TFTAEQLEELEKAFERTHYPDIYTREELAQRTKLTEARVQVWFSNRRARWRKQAGANQLAAFNHLLPGGFPPTGM
PTLPTYQLPESTYPSTTLSQDGSSTLHRPQPLPPSSMHQGGLSADSGSAYGLSSNRHTFSSYSETFMSPTASSNH
MNPVSNGLSPQVMSILSNPSAVPSQPQHDFSISPLHGGLEASNPISASCSQRSDPIKSVDSLASTQSYCPPTYSA
TSYSVDPVTAGYQYSQYGQSTYCTVPASHSLYRAAFL

>PHDP_DR

MEFPLLNKANFDKDCLYTANSEFYNSGANGGGLSVANHINHYNLMIDSSYKLCANESAIRGSLNQESSLLFSKIT
TVSEFYPATHNIGSYNTDFHLKSYGDDGLSLTDKSKQRRIRTTFTSNQLNELEKIFLETHYPDIYTREEIASKLH
LTEARVQVWFQNRRAKFRKQERHAIYIMKDKSSKLDGRKNPVAGSKYLGPSLKGPQNGHGRQMKCLYDQI

>PHOX2a_DA

MYKMDYSYLNTYDSCMAAMEASAYADFNSCSQSNGFQYNPIRSGFSSGPACAPLATASCTLGALREHQPAPYSTV
PYKFFSDASGLNEKRKQRRIRTTFTSSQLKELERVFAETHYPDIYTREELALKIDLTEARVQVWFQNRRAKFRKQ
ERAANSKASSSSGGKKPSDARSSSEDDESKESTCSPTPDSTANMSSPGARSSLSPSPAPGPASTFSPASISPALK
SSPWPSLGSGTPGPTHTHTQELLKAWQPADSMSGPFAGVLSSFHRKNNTIKSNLF

>PHOX2b_DA

MYKMEYSYLNSSAYESCMAGMDTSSLASAYADFSSCSQASGFQYNPIRTTFGATSGCPSLTPGSCSLGTLRDHQS
SPYAAVPYKLFTDHGGLNEKRKQRRIRTTFTSAQLKELERVFAETHYPDIYTREELALKIDLTEARVQIKVWFQN
RRAKFRKQERAAAAAAAAAKNGTGKKSDSREEDSKEAKATDPDSTGGPGPNPNPTSNCGGPSPTGGQVSATGNGP
VEQVKTSVAGMGPTAPTQGWASAPGTITSIPDSLGGPFASVLSSLQRQNGAKATLVKTSMF

>PHX2A_HU

MDYSYLNSYDSCVAAMEASAYGDFGACSQPGGFQYSPLRPAFPAAGPPCPALGSSNCALGALRDHQPAPYSAVPY
KFFPEPSGLHEKRKQRRIRTTFTSAQLKELERVFAETHYPDIYTREELALKIDLTEARVQVWFQNRRAKFRKQER
AASAKGAAGAAGAKKGEARCSSEDDDSKESTCSPTPDSTASLPPPPAPGLASPRLSPSPLPVALGSGPGPGPGPQ
PLKGALWAGVAGGGGGGPGAGAAELLKAWQPAESGPGPFSGVLSSFHRKPGPALKTNLF

>PHX2A_MO

MDYSYLNSYDSCVAAMEASAYGDFGACSQPGGFQYSPLRPAFPAAGPPCPALGSSNCALGALRDHQPAPYSAVPY
KFFPEPSGLHEKRKQRRIRTTFTSAQLKELERVFAETHYPDIYTREELALKIDLTEARVQVWFQNRRAKFRKQER
AASAKGAAGATGAKKGEARCSSEDDDSKESTCSPTPDSTASLPPPPAPSLASPRLSPSPLPAALGSGPGPQPLKG
ALWAGVAGGGGGGPGTGAAELLKAWQPAEPGPGPFSGVLSSFHRKPGPALKTNLF

>PHX2B_HU

MYKMEYSYLNSSAYESCMAGMDTSSLASAYADFSSCSQASGFQYNPIRTTFGATSGCPSLTPGSCSLGTLRDHQS
SPYAAVPYKLFTDHGGLNEKRKQRRIRTTFTSAQLKELERVFAETHYPDIYTREELALKIDLTEARVQVWFQNRR
AKFRKQERAAAAAAAAAKNGSSGKKSDSSRDDESKEAKSTDPDSTGGPGPNPNPTPSCGANGGGGGGPSPAGAPG
AAGPGGPGGEPGKGGAAAAAAAAAAAAAAAAAAAAGGLAAAGGPGQGWAPGPGPITSIPDSLGGPFASVLSSLQR
PNGAKAALVKSSMF

>PHX2B_MO

MYKMEYSYLNSSAYESCMAGMDTSSLASAYADFSSCSQASGFQYNPIRTTFGATSGCPSLTPGSCSLGTLRDHQS
SPYAAVPYKLFTDHGGLNEKRKQRRIRTTFTSAQLKELERVFAETHYPDIYTREELALKIDLTEARVQVWFQNRR
AKFRKQERAAAAAAAAAKNGSSGKKSDSSRDDESKEAKSTDPDSTGGPGPNPNPTPSCGANGGGGGGPSPAGAPG
AAGPGGPGGEPGKGGAAAAAAAAAAAAAAAAAAAAGGLAAAGGPGQGWAPGPGPITSIPDSLGGPFASVLSSLQR
PNGAKAALVKSSMF

```
>PITX1_DA

MNVDMASSFHLPRSASSTRETLENTSSESSDTEAAEKERSVEQRSDDGNADDPKKKKQRRQRTHFTSQQLQELEA
TFQRNRYPDMSTREEIAVWTNLTEARVRVWFKNRRAKWRKRERNQQMDLCKNSYLPQFSGLMQPYDDVYPTYTYN
NWTNKGLTPAPLSTKNFTFFNSMSPLTSQSMFSAPSSISSMSMASGMGHSAVPGMPTTGLNNIGNLNGIGGSTIN
PAMSSSTCPYGPPVSPYSVYRDTCNSSLATLRLKSKQHPSFGYSGLQSPGSSLNACQYNS

>PITX1_HU

MDAFKGGMSLERLPEGLRPPPPPPHDMGPAFHLARPADPREPLENSASESSDTELPEKERGGEPKGPEDSGAGGT
GCGGADDPAKKKKQRRQRTHFTSQQLQELEATFQRNRYPDMSMREEIAVWTNLTEPRVRVWFKNRRAKWRKRERN
QQLDLCKGGYVPQFSGLVQPYEDVYAAGYSYNNWAAKSLAPAPLSTKSFTFFNSMSPLSSQSMFSAPSSISSMTM
PSSMGPGAVPGMPNSGLNNINNLTGSSLNSAMSPGACPYGTPASPYSVYRDTCNSSLASLRLKSKQHSSFGYGGL
QGPASGLNACQYNS

>PITX1_MO

MDAFKGGMSLERLPEGLRPPPPPPHDMGPSFHLARAADPREPLENSASESSDADLPDKERGGEAKGPEDGGAGSA
GCGGGAEDPAKKKKQRRQRTHFTSQQLQELEATFQRNRYPDMSMREEIAVWTNLTEPRVRVWFKNRRAKWRKRER
NQQLDLCKGGYVPQFSGLVQPYEDVYAAGYSYNNWAAKSLAPAPLSTKSFTFFNSMSPLSSQSMFSAPSSISSMT
MPSSMGPGAVPGMPNSGLNNINNLTGSSLNSAMSPGACPYGTPASPYSVYRDTCNSSLASLRLKSKQHSSFGYGG
LQGPASGLNACQYNS

>PITX2_DA

MTSMKDPLSLDHHHHNHHVTGSKHAPLSMASSLQPLQRSVDSKHRLDVHTVSDTSSPESVEKEKGQSKNEDSNDD
PSKKKRQRRQRTHFTSQQLQELEATFQRNRYPDMSTREEIAVWTNLTEARVRVWFKNRRAKWRKRERNQQAELCK
NGFGPQFNGLMQPYDDMYPSYTYNNWAAKGLTSASLSTKSFPFFNSMNVNPLSSQTMFSPPNSISSMSMSSSMVP
SAVTGVPGSSLNSLNNLNNLSNPSLNSGVPTPACPYAPPTPPYVYRDTCNSSLASLRLKAKQHSSFGYASVQNPA
SNLSACQYAVDRPV

>PITX2_HU

METNCRKLVSACVQLGVQPAAVECLFSKDSEIKKVEFTDSPESRKEAASSKFFPRQHPGANEKDKSQQGKNEDVG
AEDPSKKKRQRRQRTHFTSQQLQELEATFQRNRYPDMSTREEIAVWTNLTEARVRVWFKNRRAKWRKRERNQQAE
LCKNGFGPQFNGLMQPYDDMYPGYSYNNWAAKGLTSASLSTKSFPFFNSMNVNPLSSQSMFSPPNSISSMSMSSS
MVPSAVTGVPGSSLNSLNNLNNLSSPSLNSAVPTPACPYAPPTPPYVYRDTCNSSLASLRLKAKQHSSFGYASVQ
NPASNLSACQYAVDRPV

>PITX2_MO

METNCRKLVSACVQLGVQPAAVECLFSKDSEIKKVEFTDSPKSRKESASSKLFPRQHPGANEKDKGQQGKNEDVG
AEDPSKKKRQRRQRTHFTSQQLQELEATFQRNRYPDMSTREEIAVWTNLTEARVRVWFKNRRAKWRKRERNQQAE
LCKNGFGPQFNGLMQPYDDMYPGYSYNNWAAKGLTSASLSTKSFPFFNSMNVNPLSSQSMFSPPNSISSMSMSSS
MVPSAVTGVPGSSLNSLNNLNNLSSPSLNSAVPTPACPYAPPTPPYVYRDTCNSSLASLRLKAKQHSSFGYASVQ
NPASNLSACQYAVDRPV

>PITX3_DA

MDFNLLTDSEARSPALSLSDSGTPQHDPGCKGQDNSDTEKSHQNHTDESNPEDGSLKKKQRRQRTHFTSQQLQEL
EATFQRNRYPDMSTREEIAVWTNLTEARVRVWFKNRRAKWRKRERNQQAELCKNGFGAQFNGLMQPYDDMYSGYS
YNNWATKSLASSPLSAKSFPFFNSMNVSPLSSQPMFSPPSSIPSMNMASSMVPSAVAGVPGSGLNNLGNLNNLNS
PTLNSAAVSAAACPYATTAGPYMYRDTCNSSLASLRLKAKQHANFAYPAVQNPVSNLSPCQYAVDRPV

>PITX3_HU

MEFGLLSEAEARSPALSLSDAGTPHPQLPEHGCKGQEHSDSEKASASLPGGSPEDGSLKKKQRRQRTHFTSQQLQ
ELEATFQRNRYPDMSTREEIAVWTNLTEARVRVWFKNRRAKWRKRERSQQAELCKGSFAAPLGGLVPPYEEVYPG
YSYGNWPPKALAPPLAAKTFPFAFNSVNVGPLASQPVFSPPSSIAASMVPSAAAAPGTVPGPGALQGLGGGPPGL
APAAVSSGAVSCPYASAAAAAAAAASSPYVYRDPCNSSLASLRLKAKQHASFSYPAVHGPPPAANLSPCQYAVER
PV
```

>PITX3_MO

MEFGLLGEAEARSPALSLSDAGTPHPPLPEHGCKGQEHSDSEKASASLPGGSPEDGSLKKKQRRQRTHFTSQQLQ
ELEATFQRNRYPDMSTREEIAVWTNLTEARVRVWFKNRRAKWRKRERSQQAELCKGGFAAPLGGLVPPYEEVYPG
YSYGNWPPKALAPPLAAKTFPFAFNSVNVGPLASQPVFSPPSSIAASMVPSAAAAPGTVPGPGALQGLGGAPPGL
APAAVSSGAVSCPYASAAAAAAAAASSPYVYRDPCNSSLASLRLKAKQHASFSYPAVPGPPPAANLSPCQYAVER
PV

>Pph13_DR

MDKGTMKRKQRRYRTTFNTLQLQELERAFQRTHYPDVFFREELAVRIDLTEARVQVWFQNRRAKWRKQEKIGGLG
GDYKEGALDLDVSYDDSAVLGQLDSALGGGGTLLPDTPPQSSNSLDNELKASYGTGAMSPSRLSPNIFLNLNIDH
LGLERGGSGLSMEWSTYPPQTQAQTHPQMDSDNQLQQHPPQQHASDPIHAGSSSHHQQQQQQHQQEQHNPQLHPG
LEFAASLSLDMTDGSSAYDEMKFLSADVDQFTIDSFKADCILSMEQSQMQAYGGHSQLVGSSNELCLDGIGMSSF
GMEEGEPKSPPSLLVLDKSLPSLSIGVEGIADLVEQLHHHQHEGGPVGGGVITGDVM

>prd_DR

MTVTAFAAAMHRPFFNGYSTMQDMNSGQGRVNQLGGVFINGRPLPNNIRLKIVEMAADGIRPCVISRQLRVSHGC
VSKILNRYQETGSIRPGVIGGSKPRIATPEIENRIEEYKRSSPGMFSWEIREKLIREGVCDRSTAPSVSAISRLV
RGRDAPLDNDMSSASGSPAGDGTKASSSCGSDVSGGHHNNGKPSDEDISDCESEPGIALKRKQRRCRTTFSASQL
DELERAFERTQYPDIYTREELAQRTNLTEARIQVWFSNRRARLRKQHTSVSGGAPGGAAASVSHVAASSSLPSVV
SSVPSMAPLAMMPGSLDPATVYQQQYDFYGSHANISVSAAAPMASSNLSPGITTTPPHHHQFYNPSANTASYIMP
GENGNTTPTGNIIVSSYETQLGSVYGTETETHQTMPRNESPNESVSSAFGQLPPTPNSLSAVVSGAGVTSSSGAN
SGADPSQSLANASAGSEELSAALKVESVDLIAASQSQLYGGWSSMQALRPNAPLSPEDSLNSTSSTSQALDVTAH
QMFHPYQHTPQYASYPAPGHAHSHHGHPHAPHPHAHPHPQYAGAHPHYPPPSSSAHFMPQNFNAAAFPSPSKVNY
TTMPPQPFYPSWY

>PROP1_DA

MDIEKPQQQFPENYSEVTVSSSADIDVQIHPRKVSQKSESVRDGGARARPYPSPSRRRHRTTFSNEQLEHLELAF
RQNHYPDIYYREELARVTKLNEARIQAWFQNRRAKQRKQDRITQKSLAVSMMPVRGPLFSTMCVSSSAMGRQCQC
PHSLPHVPQFSPVLPPGGYPPHPSSGSQFSSPSGSLPSQANRQPNDWYNQLCNIGPSPTSVLSLASVPGLESTSH
WN

>PROP1_HU

MEAERRRQAEKPKKGRVGSNLLPERHPATGTPTTTVDSSAPPCRRLPGAGGGRSRFSPQGGQRGRPHSRRRHRTT
FSPVQLEQLESAFGRNQYPDIWARESLARDTGLSEARIQVWFQNRRAKQRKQERSLLQPLAHLSPAAFSSFLPES
TACPYSYAAPPPPVTCFPHPYSHALPSQPSTGGAFALSHQSEDWYPTLHPAPAGHLPCPPPPPMLPLSLEPSKSW
N

>PROP1_MO

MEAQRRSHQEKQTKGHACGRSLPEPRVASGTLISTVDRSSEAYRRLSGTELGRPKLCPQRGRPHSRRRHRTTFNP
AQLEQLESAFGRNQYPDIWAREGLAQDTGLSEARIQVWFQNRRAKQRKQERSLLQPIAHLSTATFSGFLPESSAY
PYTYGTPPPPAPCFPHPYSHSLPSQPSTAASLALPPQPEDWYPTLHPAPTGHLPCPPPPPMFPLSLETPKSWN

>PRRX1_HU

MTSSYGHVLERQPALGGRLDSPGNLDTLQAKKNFSVSHLLDLEEAGDMVAAQADENVGEAGRSLLESPGLTSGSD
TPQQDNDQLNSEEKKKRKQRRNRTTFNSSQLQALERVFERTHYPDAFVREDLARRVNLTEARVQVWFQNRRAKFR
RNERAMLANKNASLLKSYSGDVTAVEQPIVPRPAPRPTDYLSWGTASPYSAMATYSATCANNSPAQGINMANSIA
NLRLKAKEYSLQRNQVPTVN

>PRRX1_MO

MTSSYGHVLERQPALGGRLDSPGNLDTLQAKKNFSVSHLLDLEEAGDMVAAQADESVGEAGRSLLESPGLTSGSD
TPQQDNDQLNSEEKKKRKQRRNRTTFNSSQLQALERVFERTHYPDAFVREDLARRVNLTEARVQVWFQNRRAKFR
RNERAMLANKNASLLKSYSGDVTAVEQPIVPRPAPRPTDYLSWGTASPYSAMATYSATCANNSPAQGINMANSIA
NLRLKAKEYSLQRNQVPTVN

>PRRX1a_DA

MTSSYAHVMDRQATLPNRLETAITANQDHLQAKKNFSVSHLLDLEEAREMVGAQAEESAGEAGRSMLESPGLTSG
SDTTQQENEQLNAEEKKKRKQRRNRTTFNSSQLQALERVFERTHYPDAFVREDLARRVNLTEARVQVWFQNRRAK
FRRNERAMLASKNASLLKSFSGEVTAVEQPIVPRPAPRPNDYLSWGSSPSYSAMATYSPSCANNTTAQGMNMANS
IANLRLKAKEYSLNQNRSKREQLQGTKLS

>PRRX2_HU

MDSAAAAFALDKPALGPGPPPPPPALGPGDCAQARKNFSVSHLLDLEEVAAAGRLAARPGARAEAREGAAREPSG
GSSGSEAAPQDGECPSPGRGSAAKRKKKQRRNRTTFNSSQLQALERVFERTHYPDAFVREELARRVNLSEARVQV
WFQNRRAKFRRNERAMLASRSASLLKSYSQEAAIEQPVAPRPTALSPDYLSWTASSPYSTVPPYSPGSSGPATPG
VNMANSIASLRLKAKEFSLHHSQVPTVN

>PRRX2_MO

MDSAAAAFALDPPAPGPGPPPAPGDCAQARKNFSVSHLLDLEEVAAAGRRAAGPVSGPAEARVGAAREPSGGSSG
TEAAPQDGDCPSPGRGTKRKKKQRRNRTTFNSSQLQALERVFERTHYPDAFVREELARRVNLSEARVQVWFQNRR
AKFRRNERAMLATRSASLLKSYGQEAAIEQPVAPRPTTMSPDYLSWPASSPYSSVPPYSPGGSSPATPGVNMANS
IASLRLKAKEFSLHHSQVPTVN

>PTX1_DR

MDRSSAVGGCGGGGGLGVGVVTSSATALGPGGLTNGGGGVVSGALNGLEAMSAESTGLCLQDLVSAGTANGAGSA
GSAESATTTSTALSSGSTGSSTVNGGGSSTSGTEHLHSHHSLHDSSSSVSISPAISSLMPISSLSHLHHSAGQDL
VGGYSQHPHHTVVPPHTPKHEPLEKLRIWAETGDFRDSHSSMTAVANSLDSTHLNNFQTSSTSSISNRSRDRKDG
NRSVNETTIKTENISSSGHDEPMTTSGEEPKNDKKNKRQRRQRTHFTSQQLQELEHTFSRNRYPDMSTREEIAMW
TNLTEARVRVWFKNRRAKWRKRERNAMNAAVAAADFKSGFGTQFMQPFADDSLYSSYPYNNWTKVPSPLGTKPFP
WPVNPLGSMVAGNHHQNSVNCFNTGASGVAVSMNNASMLPGSMGSSLSNTSNVGAVGAPCPYTTPANPYMYRSAA
EPCMSSSMSSSIATLRLKAKQHASAGFGSPYSAPSPVSRSNSAGLSACQYTGVGVTDVVXENALGALLNQHQHHL
QHFSGGSVGGSGVPNGMQQHSGNMLHHSNLGLDHSDLGLVGGGPSNHQDEGNHSLEGSNKSPMEAPLANSSNNNN
NDNDDEDVID

>RAX2_HU

MFLSPGEGPATEGGGLGPGEEAPKKKHRRNRTTFTTYQLHQLERAFEASHYPDVYSREELAAKVHLPEVRVQVWF
QNRRAKWRRQERLESGSGAVAAPRLPEAPALPFARPPAMSLPLEPWLGPGPPAVPGLPRLLGPGPGLQASFGPHA
FAPTFADGFALEEASLRLLAKEHAQALDRAWPPA

>REPO_DR

MEHDSFDDPIFGEFGGGPLNPLGAKPLMPTTTAMHPVMLGSVHELCSQQQQQQQQQRLPDCNTILPNAVGGGAGS
GGAGGSPNYVTKLDFVNKMGCYSPSQKYEYISAPQKLVEQQQQQQHHHQHHQHYTATPPPSHNGILLHKQSSQQQ
QQQQQQLVGILDYHPLNGKLDYSSSPKDQYEPQQLQHLYGGSPHHLDHLDHGSDGLLQDSSPVMINGGSAGGKLK
KPDEMCSQLEAGGAGVTPPSSSSTVVNGTTNGTGNANSSSSAGVGIQGAAGTAGGVAAPAAKKDGSSSKKKGDPN
GIKKKKTRTTFTAYQLEELERAFERAPYPDVFAREELAIKLNLSESRVQVWFQNRRAKWRKHEPPRKTGYIKTST
PPTATLNPGTLAPPFTSYPQTTTVTPPGSMDSWTSYQTPYELTPQFSLLSPAASPYGTYSGQYGAYVHESQLFPM
RHYEYGSPTRMEMGATTGSVAGNGDESVANGGSYQTAELQTAQQQQLADGTLVTVHAHQQQQQQQQQLNGKYLSA
EEAKYVHLQCHQSGGGLELSPGASCHLVEAQGQHYVTTATGGAASAGGTSSDDNDSGGMQTVIKSEEVSQQQQQQ
QGQSYVLPPFLH

>RX_DR

MSGSGGVANSSSTAAANNTAATFQHIFEQLVQQGGGNHKLPPKQLEQLRHLLGNVRDAKNLQMIVEKFKNLEQFH
EHYAAHLANNNTVISTEDSNDLVKDNARKYGSGGQTLTPRHTIDAILGLKNRNGAANGSGRNPETVSDGSVDPSL
GDDDATDLRCGMTLTQLRSMDNHMASMLQQHAKNGGALPYGPPTPPGGQQPQVPNATPLHHGQQMGGQAGHATHA
GHGHPTHHGHAPFGYHNAFGFGQGGHGYGHPEEAAGNYLNSMHQMVEANQLQTGANGANPPPAPLPPSSFGSHQQ
HLAALAAQAQEQQNQHSKYAKSSPTGAGPPPPPGAYFMESQTAPVAPSQINYDERSMSSASDLEEDDDDAAKLQL
DVTSPPTPSPRGQLAAKRKSAGVFCDDNEPKLANGQLPGGNYGIRPRSMEEVHHQQQSHHHQQQQQQQQQQQQLQ
QQQGFQHDFRNSGNGNPNGNSNSGDHGERLNADSDSLVNGSCASSEDLNQTNSSEQGEKITSGSDDEGQDDNCAK
KKHRRNRTTFTTYQLHELERAFEKSHYPDVYSREELAMKVNLPEVRVQVWFQNRRAKWRRQEKSESLRLGLTHFT

QLPHRLGCGASGLPVDPWLSPPLLSALPGFLSHPQTVYPSYLTPPLSLAPGNLTMSSLAAMGHHHAHNGPPPPHV
GHGGHGQPQPPPPPPPPHGVPHPHGSHHVVPLSHLSPHLSRMSPHATSLGSPHHGVTPLGTPLHSSLPPSSTATTV
AVSSSQSSSSSASLECSGPDVCMSPQNLSIGNADSNGDGRDLSSDLDAGSTSSNPGSSLDKCAASANIELLDVGR
DSPPPPPTPTGKGSSTPPTDMRSNSIATLRIKAKEHLDNLNKGMVSIV

>RX_HU

MHLPGCAPAMADGSFSLAGHLLRSPGGSTSRLHSIEAILGFTKDDGILGTFPAERGARGAKERDRRLGARPACPK
APEEGSEPSPPPAPAPAPEYEAPRPYCPKEPGEARPSPGLPVGPATGEAKLSEEEQPKKKHRRNRTTFTTYQLHE
LERAFEKSHYPDVYSREELAGKVNLPEVRVQVWFQNRRAKWRRQEKLEVSSMKLQDSPLLSFSRSPPSATLSPLG
AGPGSGGGPAGGALPLESWLGPPLPGGGATALQSLPGFGPPAQSLPASYTPPPPPPPFLNSPPLGPGLQPLAPPP
PSYPCGPGFGDKFPLDEADPRNSSIAALRLKAKEHIQAIGKPWQAL

>RX_MO

MHLPGCAPAMADGSFSLAGHLLRSPGGSTSRLHSIEAILGFTKEDGILDTFPAERSSRSSKERDPRLGAQPACPK
APAGGSESSPPAAPGFVPEYEATRPCYPKEQGEARPSPGLSVGPAAGDSKLSEEEPPKKKHRRNRTTFTTYQLHE
LERAFEKSHYPDVYSREELAGKVNLPEVRVQVWFQNRRAKWRRQEKLEVSSMKLQDSPLLSFSRSPPSSALAPLG
TGPGSGSGPPGSALPLEPWLGPPLPGGGATALQSLPGFGPPGQGLPASYTPPPPFLNSAPLGPGLQQLGPPPAYP
CAPAFGDKFSLEEAYPRNSSIAALRLKAKEHIQAIGKPWQAL

>RX1_DA

MHLSLDTMNMVDDSCLSPSNFHDMVKAGGAVVGTRVHSIDVILGFNKDQDSLLNPGDNAVAHKVGGESLGEPGKL
DQRVQPYGHLPPLRDGSEQPTFHDADMFSNKCDGDLGDLRKAIESDSKSPDSADGEQPKKKHRRNRTTFTTYQLH
ELERAFEKSHYPDVYSREELAMKVNLPEVRVQVWFQNRRAKWRRQEKIDASTMKLHDSPMLSFNRPSMHPTVGPM
NNSLPLDPWLPSPLSSATPVHSIPGFMGPTQGLQTGYPGHSFLSPPQPMAQSMQPMAPPPYQCPPPFTDKYPLED
VDQRSSSIAALRMKAKEHIQSMDKTWQPMC

>RX2_DA

MHLSPDTMNMVDDGCLSTESYHDLGKGGVSGISSRVHSIDVILGFSKDQDPLLEPIGTHKVDEDQLEEQEKQVNS
DPYSHLQIPDQTQQQQSVYHDTGLFSTDKCDADLGDPRSNVESDSRSPDIPDEDQPKKKHRRNRTTFTTYQLHEL
ERAFEKSHYPDVYSREELAMKVNLPEVRVQVWFQNRRAKWRRQEKMDTGTMKLHDSPIRSFNRPPMAPNVGPMSN
SLPLDPWLSSPLSSATPMHSIPGFMGPGQSLQPTYTAHPGFLNTSPGMMQNMQPMPPPPYQCQPVFNDKYPLEDV
DRSSSIAALRMKAKEHIQSMDKTWQPM

>RX3_DA

MRLVGSQYKDMEDRLSPSARLVRSPGSQTRIHSIESILGFKGETLFHPAFPYGSGKTGKDTEHLSPKKDSNKHFD
GVCRSTVMVSPDLPDADGGKLSDDENPKKKHRRNRTTFTTFQLHELERAFEKSHYPDVYSREELALKVNLPEVRV
QVWFQNRRAKWRRQEKLEVSSIKLQESSMLSIPRSGPLSLGSGLPLEPWLTGPISTSSSPLQSLPSFITPQQAVP
ASYTPPQFLSSSTLNHSLPHIGAVCPPYQCSGFMDKFSLEEADPRNTSIASLRMKAKEHIQSIGKTW

>SEBOX_DA

MALFYDQSFDLGLVQKINMETESDFIFNTNMLQFTDVTTKHMLSSPELDRTGHVEGQRKRKRTIFSRAQLSELER
AFMITPYPDITLRERLAALTLLPESKIQVWFQNRRARSMKSKKLITPVSRRSPAKDCTFPATHPDLNLEQSPEAN
KSLRHHQQSLIRQALNPWPQNRPPISPDLPEILQWANRNSETPGDSSFSSCPSERIQHPFPNQSSSVWQMNCFAA
HPEGLKSYCTTSQALYSSVSVDQMIPAHPSSLEEALQRQALTHYPQTSLGDISDLIYKAAVVTNLEEC

>SEBOX_HU

MGGSGVGTAWHGPLARPSGTLPFASSMPSPVDASSADGGSGLGSHRRKRTTFSKGQLLELERAFAAWPYPNISTH
EHLAWVTCLPEAKVQVWFQKRWAKIIKNRKSGILSPGSECPQSSCSLPDTLQQPWDPQMPGQPPPSSGTPQRTSV
CRHSSCPAPGLSPRQGWEGAKAVAPWGSAGASEVHPSLERATPQTSLGSLSDLIYALAIVVNVDHS

>SEBOX_MO

MASPVEASPGCASGLGPHRRKRTTFSVGQLVELERVFAARPYPDISTREHLAQVTHLPEAKIQVWFQNRRAKRIK
DRKPGALNSRLELPPNSCSLPDTPQLPWDPGTSSHPLHPTSSAQYTSACPPQTSCLGPILGPGQSWSGAKVAAPW
GTSGASGIHSSLEQIVPQTSLGNLSDLIYTSAIVTNVDHF

>SHOX_DA

MEELTAFVSKSFDQKTKESSKESITYREVLESGLARARELGNSETNLQEITETNNNHCPVHLYKEHVELEKEKLK
EFSVTRASDGIYDCKDKKEDVKSEDEDAQSKLKQRRSRTNFTLEQLNELERLFDETHYPDAFMREELSQRLGLSE
ARVQVWFQNRRAKCRKQENQMHKGVILGTASHLDACRVAPYVNMGALRMPFQQVQAQLQLEGVGTHSHPHLHPHL
AAHAPYLMFPPPPFGLPIASLADSASAAAAVVAAAKSNSKNSSIADLRLKARKHAEALGL

>SHOX_HU

MEELTAFVSKSFDQKSKDGNGGGGGGGGKKDSITYREVLESGLARSRELGTSDSSLQDITEGGGHCPVHLFKDHV
DNDKEKLKEFGTARVAEGIYECKEKREDVKSEDEDGQTKLKQRRSRTNFTLEQLNELERLFDETHYPDAFMREEL
SQRLGLSEARVQVWFQNRRAKCRKQENQMHKGVILGTANHLDACRVAPYVNMGALRMPFQQVQAQLQLEGVAHAH
PHLHPHLAAHAPYLMFPPPPFGLPIASLAESASAAAVVAAAAKSNSKNSSIADLRLKARKHAEALGL

>SHOX2_DA

MEELTAFVSKSFDQKVKEKKEVITYREVLETGSVRNRESLSADPNREEISSITRSGVRSSPVREADMLASERSRD
SSSPKLTDGNTDMKERKEDCKPLEDETQTKIKQRRSRTNFTLEQLNELERLFDETHYPDAFMREELSQRLGLSEA
RVQVWFQNRRAKCRKQENQLHKGVLIGAASQFEACRVAPYVNVGALRMPFQQASDSHCNVPPFSFQVQAQLQLDS
AVAHAHHHLHSHLAAHAPYMMFPAPPFGLPLATLAAESASAASVVAAAAAAKTTNKNSSIADLRLKAKKHAAALG
L

>SHOX2_HU

MEELTAFVSKSFDQKVKEKKEAITYREVLESGPLRGAKEPTGCTEAGRDDRSSPAVRAAGGGGGGGGGGGGGGGG
GGVGGGGAGGGAGGGRSPVRELDMGAAERSREPGSPRLTEVSPELKDRKEDAKGMEDEGQTKIKQRRSRTNFTLE
QLNELERLFDETHYPDAFMREELSQRLGLSEARVQVWFQNRRAKCRKQENQLHKGVLIGAASQFEACRVAPYVNV
GALRMPFQQDSHCNVTPLSFQVQAQLQLDSAVAHAHHHLHPHLAAHAPYMMFPAPPFGLPLATLAADSASAASVV
AAAAAAKTTSKNSSIADLRLKAKKHAAALGL

>SHOX2_MO

MEELTAFVSKSFDQKVKEKKEAITYREVLESGPLRGAKEPGCVEPGRDDRSSPAVRAAGGGGGAGGGGGGGGGG
GGAGGGGAGGGAGGGRSPVRELDMGAAERSREPGSPRLTEVSPELKDRKDDAKGMEDEGQTKIKQRRSRTNFTLE
QLNELERLFDETHYPDAFMREELSQRLGLSEARVQVWFQNRRAKCRKQENQLHKGVLIGAASQFEACRVAPYVNV
GALRMPFQQDSHCNVTPLSFQVQAQLQLDSAVAHAHHHLHPHLAAHAPYMMFPAPPFGLPLATLAADSASAASVV
AAAAAAKTTSKNSSIADLRLKAKKHAAALGL

>SP83252

MVAGVMSEEEYKTALQKQTLLMQSPREGTQYTPGSPTRSRTSSPSTSSTIECGGAGGGQNDGAEGEERKSPLSNG
DLLLKKKRRYRTTFTSFQLRELEKAFERTHYPDVFTREDLANRVELTEARVQVWFQNRRAKWRKKEKQQGLPSPT
AQPIAATSTPPALISSHETSTRVITLNPSSLLQSPPDTTTTTILATPEASVALPTLPQATTPVPLAHFLSGAASG
ATQPRQWHHPPALNFLSTTPSTNTTTLRAITLPNGGIVLQPTLATLPATAPAGTTQIIGLHPAQTAAASAGVFAP
QILSSPFQLAQIPVSMATGLSSGVITLQLAQQNVTSS

>SP86105

MATGNPAGKFGGRHYSLDSPGSLTSPPAYDMPLMPGAGPEQKPGVSALSMQNSCAGGQMSMSSCSPPFSQINPLV
YSHPGHAGLLPLNSTSLATVHAPNSHAGMYLHHPSAGPMVPSSMSIGRSEPYRKGKQRRHRTNFTSHQLEELEKA
FEKTRYPDVFMREELAMKISLTEARVQVWFQNRRAKWRKAEKAAAAANQKDSKDSEDQESPVSSPAPSDGKGASS
PKKSTSSPLSSPPARKMHPSTGGNESWTSSPVDSFSPPTFHSPPQSPSIIPTSCPTPNPIHSYTPESPFAAMGIV
SHQTPTGTGAPTGAGHFTNSYLQSMTRYAPHC

>SP87530

MFTIERLLNLQPTKSSKPPAEKYVDLTIGEKEERRHKQRYTSSCDSAAESDKDSIISDDYSTSPVQSPKGSVNII
MKSKRNRTTFTAHQLDELEMIFRQTHYPDVLLREKLAQRIGLPESRVQVWFQNRRAKWRKREKLIAAADAQMKAL
SNNHSIHHSNPSPSLSIPPAATPAGLYWSPAANSVTPTWTLPTATPGSIISPSHLPGAATSRLSQPPPLIPLSSI
NSCSPTSSTTGGSLLSTLTSNNSAILSPPLLQRREGGSSNGAGVLMAAAGVNPFSLYLSTLTPQQSIIIQQ

>SP87700

MPVAMMQKHSPPELEGTPPAAIANINGGLLVAGSDSPKLENGIESPGSSSVKSEDGGSDTERGSSKSSSSSKRQQ
RRFRTTFTSYQLQELEAAFAKTHYPDVFMREDLALRINLTEARVQVWFQNRRAKWRRAQKASQLAMGDIMSGRGV
SMMAGGGGIPHMHGMMPHNFAATLPTNLSPGSSVLMTPKSAGTSPSAHMFQNKPFVLHHPNQLPSSSMSAYPTPW
NTQNQFVFPSPTTMTFSNNHSPLSSSSPPVCSGVMTPQW

>SP89232

MEQTGEALSDEGPKVAPINTNSYSSDFSVSTRLLQADNPTQGASSSYIAPTYPTDNYSSNPPPPPNGWQFLLPSD
MGTARGHPHSTGQYEPAPNASLSLMPPPPYQSIAYCNGYPGSKIGISSLPSQSLDVHGHHHTSIPPLHHKFDIGY
NSWNVGASSSTGSGSNSGKPLPPVGTLTSPPSVSYPSSPSYNDQDNPDSSRPPMAIKEESYIESDDRGEEDYGPS
DEESDSNSTTDKDAEPKKPKRTRTAYSNSQLDQLELIFATTHYPDVFTREDLSRRLGIREDRIQVWFQNRRARFR
KQERTGSISCRSRYRQKRLEKLQHNFMPSSAGYPPTHYPPSVVGGGNTLSLVSSPSVSTTSPVSYDFSQSQGFSF
QTSFYPVTPNYRSYSTGTAFQYPGGLASLFQTAPSTAEGK

>SP91658

QDNPDSSRPPMAIKEESYIESDDRGEEDYGPSDEESDSNSTTDKDAEPKKPKRTRTAYSNSQLDQLELIFATTHY
PDVFTREDLSRRLGIREDRIQVWFQNRRARFRKQERTGSISCRSRYRQKRLEKLQHNFMPSSAGYPPTHYPPSVS
QGFSFQTSFYPVTPNYRSYSTGTAFQYPGGLASLFQTAPFDCRGK

>toe_DR

MATSTSPGPTRTTAAIRVSLPGAAPAIPATATAGSQSPGAIMPMAAVPLPSHLQLLGSSAGGVGQPAAITPVSPT
AATTVLAQPHSLSPSTPILTQGAPPAAALGGIFCGGSAAAGPGAAAAAATNLNALASQHRLLELSRFGLRGYDLA
QHMLSQQGAVSKLLGTLRPPGLIGGSKPKVATPTVVSKIEQYKRENPTIFAWEIRERLITEGVCTNATAPSVSSI
NRILRNRAAERAAAEFARAASYGYAIHPTHPHPYTSFPTWPAHHPLWGAVPLATPPGGGPAGAGGALQPGGSGSS
YGSDGNMSSNPNSSNSNTTHSNGHNTNSGSGCGDSSAGSGRLSLPALSPDSGSRDSRSPDADANRMIDIEGEDSE
SQDSDQPKFRRNRTTFSPEQLDELEKEFDKSHYPCVNTREKLAARTALSEARVQTSEMAAPPAGQLDQAARLALD
IELTHAVGQSGGQSGQSNPSSSSSCSSRIWPTEAAISVQHQQHVQHQQQQQQSTVQHHQSRQQNEQQNQQRQQQ
PAHGRASSGGGHCLTHSISSIINGR

>toy_DR

MMLTTEHIMHGHPHSSVGQSTLFGCSTAGHSGINQLGGVYVNGRPLPDSTRQKIVELAHSGARPCDISRILQVSN
GCVSKILGRYYETGSIKPRAIGGSKPRVATTPVVQKIADYKRECPSIFAWEIRDRLLSEQVCNSDNIPSVSSINR
VLRNLASQKEQQAQQQNESVYEKLRMFNGQTGGWAWYPSNTTTAHLTLPPAASVVTSPANLSGQADRDDVQKREL
QFSVEVSHTNSHDSTSDGNSEHNSSGDEDSQMRLRLKRKLQRNRTSFSNEQIDSLEKEFERTHYPDVFARERLAD
KIGLPEVWFSNRRAKWRREEKMRTQRRSADTVDGSGRTSTANNPSGTTASSSVATSNNSTPGIVNSAINVAERTS
SALVLGGEANTTHTSSESPPLQPAAPRLPLNSGFNTMYSSIPQPIATMAENYNSSLGSMTPSCLQQRDAYPYMFH
DPLSLGSPYVSAHHRNTACNPSAAHQQPPQHGVYTNSSPMPSSNTGVISAGVSVPVQISTQNVSDLTGSNYWPRL
Q

>UNC4_DR

MVLETSEGDTKKFLTNNNNSSASRNNNSHNNNNNNHSPKEIPEETGRSSSTSSNSIPNAHRTNAGQHLLGGSPSS
ACSTSVSGCGMPSEGLHPTAALQLYAAAAQLAPNGVRVPPWGPFLQFGVPGVFGPNGPFLGRPRFDAASAGGHPN
SAAAAAAATQMAAVNASNAFANLTGLSAAALRNVSAAQTTAVAAVASTVATIQHRLMIGNRQSLPPAGPPSEGSN
EDGGFPGDGDDDSSAAKRRRSRTNFNSWQLEELERAFSASHYPDIFMREALAMRLDLKESRVAVWFQNRRAKVRK
REHTKKGPGRPAHNAQPQTCSGEPIPPNELKAKERARRRKKLAKAIDRQARKLQAKGITVDLEALKAEYISQHKA
NGTFSDSDLEDDGIQIDVVGGTDSDDEGDSDAVSPVRLGGGGGGGGGTGGGGGGGASSSLHCGLDGDGDSSRAGS
FIGGGGSLGSPSSVAPPSMLSNGAVQFGKLEPMDGNESEERDRERDSPKPLLFPAKAFHQLNLQSSQQHHGLVVG
QGQGSGSGHGHGHQHNHHQHHLHHGSASASAAAAVANLVHQTSPISMRRSNPFSIESLLFNNT

>UNC4_HU

MMDGRLLEHPHAQFGGSLGGVVGFPYPLGHHHVYELAGHQLQSAAAAASVPFSIDGLLGGSCAAAASVVNPTPLL
PAACGVGGDGQPFKLSDSGDPDKESPGCKRRRTRTNFTGWQLEELEKAFNESHYPDVFMREALALRLDLVESRVQ
VWFQNRRAKWRKKENTKKGPGRPAHNSHPTTCSGEPMDPEEIARKELEKMEKKKRKHEKKLLKSQGRHLHSPGGL
SLHSAPSSDSDSGGGGLSPEPPEPPPPAAKGPGAHSGAAGTAPAPPGEPPAPGTCDPAFYPSQRSGAGPQPRPG
RPADKDAASCGPGAAVAAVERGAAGLPKASPFSVESLLSDSPPRRKAASNAAAAAAAGLDFAPGLPCAPRTLIGK
GHFLLYPITQPLGFLVPQAALKGGAGLEPAPKDAPPAPAVPPAPPAQASFGAFSGPGGAPDSAFARRSPDAVASP

GAPAPAPAPFRDLASAAATEGGGGDCADAGTAGPAPPPPAPSPRPGPRPPSPAEEPATCGVPEPGAAAGPSPPEG
EELDMD

>UNC4_MO

MMDGRLLEHPHAQFGGSLGGVVGFPYPLGHHHVYELAGHQLQSAAAAAAAASVPFSIDGLLSGSCAAAAASVVNP
TPLLPAACGVAGESQPFKLADSGDPDKESPGCKRRRTRTNFTGWQLEELEKAFNESHYPDVFMREALALRLDLVE
SRVQVWFQNRRAKWRKKENTKKGPGRPAHNSHPTTCSGEPMDPEEIARKELEKMEKKKRKHEKKLLKSQSRHLHS
PGGLSLHSAPSSDSDSGGGGLSPEPPEPPPPTAAAKGPGAHGSGIAGSAPVPPGEPPAPGTCDPAFYPSQRSGAG
SQPRLGRPADKDTVPCGPGAAATAGLPKASPFSVESLLSDSPPRRKATPANAAATAGLDFTPGLPCAPRTLIGKG
HFLLYPITQPLGFLVPQAALKGGAGPELVPKDAPPAPPAPPAPPAQASFGTFPGPGGAADPAFARRSPEVVASPG
PPAPASFRDLTAAAAESGAGDCADVGTVCPAASPPPPLETSPGPGPRAPSPPGEPATCGAAEPGAATGPSPPEGE
EVDMD

>UNCX_DA

MMDSRILDPPHAQFGGSLGGMVSFPYHLSHHHVYELAGHQLQSTAAVPFSIDGLLNGSCTASVVNSNPLLSSGCG
MNGDNQQYKLTDSGDPDKDSPGCKRRRTRTNFTGWQLEELEKAFNESHYPDVFMREALALRLDLIESRVQVWFQN
RRAKWRKKENTKKGPGRPAHNSHPTTCSGEPMDPEEIARRELERLEKKKRKQERRLLKSQNKLLPGDLFHTPGSD
SDSGLSQITDSEQSLHCDMGRNQTQPSCDQTPQKLQNQRNADQDASGSELDSSDSGQQSNLCSNSRSSALQKLNP
FSVESLLSDSRPRRKPPMDFPVPTPRPLIGKGHFLLYPITQPLGFIVPQTALKSTAPGPDTENGQKGPATDITFA
GNPGHRNAKENNSVNNNSGARAIKGQVSQSGNISCSTQSSSPQTTINGHSAGSCNERCPQDIELELVDPKSPQSE
KKEQSLPDYPPQNSESATTSKDTDKDSVDVDME

>UNCX4_DA

MMDSRILEHPHAQFGGSLGSMVGMGFPYHLGHHHVYDISGHQLQSAAAVPFSIDGLLNGSCSGSVANSNPLLGSG
CGVNGDSQYKLGDGGDPDKESPGCKRRRTRTNFTGWQLEELEKAFNESHYPDVFMREALALRLDLVESRVQVWFQ
NRRAKWRKKENTKKGPGRPAHNSHPTTCSGEPMDPEEIARRELERLEKKKRKQERKLLKSQNKLLAGELFHTPGS
DSDSGVSQSTDSESTPHTGPQHSAHRQQTEHICEQHARHQRASTVNETAEPMDSTRNSGLCPANGITRASTLQKL
NPFSVESLLADSSPRRKTILDFSQLPPQRPLVGKGHFLLYPITQPLGFIVPQTAMKQSHDSGNSGHHCSTTDTST
SNQKNVNHLCRDNTGASDELQRETKNSSIQSPSTSSEKCFSESNSPQKESENDSESTVTNSSQKESISANLSEYS
DRKSRSSADTNTDGEDVDMD

>VSX1_DA

MTGREEATDEKPKVKLYPSFGIDKSRLNGSGFRSKGFAITDLLGLESELQPHQSGTGAGPNGEGQSAAVGGFSFP
GGSLPLGLGFLCSLAAQQPPGAPCFLPSHIPLLQSRTESHFMQNLEQQRDVYSDDDCLSGDRNDGKNSGNSQKRK
KRRHRTVFTSHQLEELEKAFNEAHYPDVYAREMLAMKTELPEDRIQVWFQNRRAKWRKREKCWGRSSVMAEYGLY
GAMVRHSIPLPESIINSAKSGMMGSCAPWLLGMHKKSMEIVKKAPGTPESTHSDTYSEEHKGKDSDTTWSSGANG
ADDSEDMAIDLSSTSKQENKSTLKRSPPRTENSSDSENES

>VSX1_DR

MQATKMRTPFAIQEILGLGAYQQQATASATPSEAPPSPAATPPPTANTPANGSLSAVQQQQQQQQQQQQQQQQLQ
QQQQQAIDSLRESRSISPDVSRLSAAAAAAAAAAAAHCQLPVFNPANFYAAAGYAAAADPGNAAAAHHHHMTTLAA
AAYLRGFLPPGFSTPHEFRGHFQQHFGAQFAEQAAARGQEYASSLANASGDEQSPSKNSSSSGNGGSGGSGSGSG
GGNPTSNGLGHLGSPTASNGGSGGGAGVGGSGGGSGTSGSSSSTSGNGGNGGSSGSRSSPAGPHHSAALAAHQHA
AAAAAAAHHHAAAAAAAHHAHHAHAQAHAHAHAHVHAHAAHAAHAAHAHAHHAEAFLGAAGGAGGNPNSLLAAHG
GDVLVGPGGTGPGGKKKNKRRHGRTIFTSSQLEELEKAFKEAHYPDVSARELLSMKTGLAEDRIQVWYQNRRAKW
RKTEKCWGHSTKMAEYGLYGAMVRHSLPLPETIIKSAKEDESVAPWLLGMHKKSLEAAELLKSDESDRETPTSDD
TNTSYSAGSTHNSPISSFSISRLLFDAPPPAAGSAGGKGHHHHHHHHKGHHHAHVHAQAQAHAHMLLHHQQQQQQ
QQQQQQHHHHLHAAENSLQQQQVAGTGSGSGSGSGSGTGAGSTGPPAHHPYNQQQHLHHLQQLQQQQQQQQQQAQ
QQQQHHHHQHHHSVHHMHHHNAAAGYAEAAVAAAAAAAAVAAAANARNAAAAAAVAAASASASAAVTIAESEAEE
EQDDIEDMELDKDCEVEEDVEPVDNDTNTNNEHDNECDADADVEADVDADADADPEIDICDDHDEEMRLQEQLLL
KVKQEQAHLGSR

>VSX1_HU

MTGRDSLSDGRTSSRALVPGGSPRGSRPRGFAITDLLGLEAELPAPAGPGQGSGCEGPAVAPCPGPGLDGSSLAR
GALPLGLGLLCGFGTQPPAAARAPCLLLADVPFLPPRGPEPAAPLAPSRPPPALGRQKRSDSVSTSDEDSQSEDR

NDLKASPTLGKRKKRRHRTVFTAHQLEELEKAFSEAHYPDVYAREMLAVKTELPEDRIQVWFQNRRAKWRKREKR
WGGSSVMAEYGLYGAMVRHCIPLPDSVLNSAEGGLLGSCAPWLLGMHKKSMGMIRKPGSEDKLAGLWGSDHFKEG
SSQSESGSQRGSDKVSPENGLEDVAIDLSSSARQETKKVHPGAGAQGGSNSTALEGPQPGKVGAT

>VSX1_MO

MTGRDGLSDARSRSRALAPGCPPTGSRLRSFAINDLLGLEADLPTPAEPGLRSNSGDPAEAIGSGPGPGPGLCGS
CPARGALPLGLGLLCGFGAQPPSAAAAARARCLLLADLRLLPSAGPEPAVAQGPVHPPPALGSQQRSESVSTSDG
DSPSEEKNDPKMSLILGKRKKRRHRTVFTAHQLEELEKAFGEAHYPDVYAREMLAAKTELPEDRIQVWFQNRRAK
WRKREKRWGGSSVMAEYGLYGAMVRHCIPLPDSVLNSADSLQGSCAPWLLGMHKKSTGMRKPESEDKLAGLWEFD
HLKKGANKDEDGPERGPDETTQNPENSLEDVAIDLSSSSRQETKKMPPGSSTQLPQPPQVGAS

>VSX2_DA

MTGKDGAVLSESLNKSKSLCATENGGNNNPHLSKSSITHPPKCTGFGIQEILGLNKEPSSAPRSTLDSFPAGAHL
LASRSMLGPAGVGVGVGMGLIGPGGIPSFYSQPAFLEVLSDAQNVHLQPLSRTVGPLEHNQSASSDSDDVSSSER
KMSKSSLSQSKKRKKRRHRTIFTSYQLEELEKAFNEAHYPDVYAREMLAMKTELPEDRIQVWFQNRRAKWRKREK
CWGRSSVMAEYGLYGAMVRHSIPLPESILKSAKDGIMDSCAPWLLGMHKKSLETAGHQSNEKSDVTQTPTNPKPD
EAEAEERRTESPMSKEELRENSIAALRAKAQEHSAKVLGTVSSERLEHNMETTATEEKSSEQIDAKEEEKSS

>VSX2_DR

MPQRSPFAIQELLGLAVAAATEAPTDLTTTAGATVAKERQTPTPPKTTNATMATAATSAATAATPTNAAEGNLTS
VSEPQQQPQQQQQEQQHHQPHHHQYREHHQMTMAAASRMAYFNAHAAVAAAFMPHQLAAAVHHHHQHQHQHHPHH
HPHHPHGAVGGPPPPPPMQHHHPHHPHHPLLHAQGFPQLKSFAAGAGTCLPGSLAPKDFGMESLNGFGVGPNSKK
KKKKRRHSRTIFTSYQLEKLEEAFKEAHYPDVYAREMLSLKTELPEDRIQVWFQNRRAKWRKTEKVWGGSTIMAE
YGLYGAMVRHSLPLPDTILKSAKDNDAVAPWLLGMHRKSIEAQSALKDDSGVSDHEDSAGSKSAHSEDLSRSRCH
ALSSSTESLNVVSPAPSSCPTSASTSAPPTSTSGYAGAASSAATTPTGASTTNSSSSPHIELGSPSPQQQQHLQL
QQQQQQQASLYLGAGAVVTGCAPPSYHPLLDAANAAGAGAAASSKDFHMIMNTAVAAAAAAAAQQQHQQQQQQQQ
ATATAPGLHAHNLAAALMEHDPDAFRNNSIACLRAKAQEHQARLLNNGGLFLQVRRFAQGQAQIQDPSDMLKLSE
EHNNNSPIPIPPAHSTVNTQVNVKMELTANGGSMKNYEEA

>VSX2_HU

MTGKAGEALSKPKSETVAKSTSGGAPARCTGFGIQEILGLNKEPPSSHPRAALDGLAPGHLLAARSVLSPAGVGG
MGLLGPGGLPGFYTQPTFLEVLSDPQSVHLQPLGRASGPLDTSQTASSDSEDVSSSDRKMSKSALNQTKKRKKRR
HRTIFTSYQLEELEKAFNEAHYPDVYAREMLAMKTELPEDRIQVWFQNRRAKWRKREKCWGRSSVMAEYGLYGAM
VRHSIPLPESILKSAKDGIMDSCAPWLLGMHKKSLEAAAESGRKPEGERQALPKLDKMEQDERGPDAQAAISQEE
LRENSIAVLRAKAQEHSTKVLGTVSGPDSLARSTEKPEEEEAMDEDRPAERLSPPQLEDMA

>VSX2_MO

MTGKAGEALSKPKSETVAKSTSGGAPARCTGFGIQEILGLNKEPPSSHPRAALDGLAPGHLLAARSVLSPAGVGS
MGLLGPGGLPGFYTQPTFLEVLSDPQSVHLQPLGRASGPLDTSQTASSDSEDVSSSDRKMSKSALNQTKKRKKRR
HRTIFTSYQLEELEKAFNEAHYPDVYAREMLAMKTELPEDRIQVWFQNRRAKWRKREKCWGRSSVMAEYGLYGAM
VRHSIPLPESILKSAKDGIMDSCAPWLLGMHKKSLEAAAESGRKPEVERQALPKLDKMEQEERAPEAQAAISQEE
LRENSIAALRAKAQEHSTKVLGTVSGPDSLARNAEKPEEEDATEEDRPAEKLSPPQLEDMA

>CEH10

MAVDEMQLAASMQMSNQMSNSQRMSFAIHEILGIQGNAYLSHGYCPQGTFLSNQPNFMDIGSCGVSGFDRQLCGT
DSIGNAPAPLMYRMPISTSEVITSEPQTHVSSSSILSSATTSNSSGGGSSGGGGKASKRKKRRHRTIFTQYQIDE
LEKAFQDSHYPDIYAREVLAGKTELQEDRIQVWFQNRRAKWRKTEKTWGKSTIMAEYGLYGAMVRHSLPLPETIT
KSAEAADPQQSAAPWLLGMHKKSMEAAAHLESVEKCDMSDSDDDDRPVTPPVQRQVKNEYKPRIIEHVQQQAPQH
QSSLNFDTSLVVSSSLSQLHFQDSQMIPNNNASLKLYHDYNNPM

>HyHox1

MYSVSGDITFAPKIYNHSPILTTNNTCKTTRSERGYSHTLEYFPNRPIIAKSQLNNKTPIWPQQNASKITNYTAP
YNKNIDENYRRPLELFKQETPYNKARKKHFEHYQLYQNTKEHALLKNVAAEKKITTELIVLDDLKEYKGEYQSND
SQSSDNQSNDAFRKRCSFGHSKIIELEKEFKYNKYLSRDRRVEFARNLELSESQIKIWFQNRRMKQKKEQIVNDL
TKKDDDSPWFRIEKLHSLTEPFLSTNQVSPMFHTFLN

2) Homeodomain alignments of PRD class used for phylogenetic analysis

```
>al_DR
QRRYRTTFTSFQLEELEKAFSRTHYPDVFTREELAMKIGLTEARIQVWFQNRRAKWRKQE
>ALX1_DA
KRRHRTTFTSAQLEELEKVFQKTHYPDVYVREQLAMRTELTEARVQVWFQNRRAKWRKRE
>ALX1_HU
KRRHRTTFTSLQLEELEKVFQKTHYPDVYVREQLALRTELTEARVQVWFQNRRAKWRKRE
>ALX1_MO
KRRHRTTFTSLQLEELEKVFQKTHYPDVYVREQLALRTELTEARVQVWFQNRRAKWRKRE
>ALX3_HU
KRRNRTTFSTFQLEELEKVFQKTHYPDVYAREQLALRTDLTEARVQVWFQNRRAKWRKRE
>ALX3_MO
KRRNRTTFSTFQLEELEKVFQKTHYPDVYAREQLALRTDLTEARVQVWFQNRRAKWRKRE
>ALX4_HU
KRRNRTTFTSYQLEELEKVFQKTHYPDVYAREQLAMRTDLTEARVQVWFQNRRAKWRKRE
>ALX4_MO
KRRNRTTFTSYQLEELEKVFQKTHYPDVYAREQLAMRTDLTEARVQVWFQNRRAKWRKRE
>ALX4a_DA
KRRNRTTFTSYQLEELEKVFQKTHYPDVYAREQLALRTDLTEARVQVWFQNRRAKWRKRE
>ARX_DA
QRRYRTTFTSYQLEELERAFQKTHYPDVFTREELAMRLDLTEARVQVWFQNRRAKWRKRE
>ARX_HU
QRRYRTTFTSYQLEELERAFQKTHYPDVFTREELAMRLDLTEARVQVWFQNRRAKWRKRE
>ARX_MO
QRRYRTTFTSYQLEELERAFQKTHYPDVFTREELAMRLDLTEARVQVWFQNRRAKWRKRE
>CEH10
KRRHRTIFTNYQLEELEKAFKESHYPDVYARENLSLKIDLPEDRIQVWFQNRRAKWRKKE
>CG11294_DR
QRRNRTTFTPQQLQELEALFQKTHYPDVFLREEVALRISLSEARVQVWFQNRRAKWRKQA
>CG13141_DR
QRRSRTNFTLDQLNELERLFEETHYPDAFMREELSQRLGLSEARVQVWFQNRRAKCRKHE
>CG32532_DR
RRRHRTTFTQEQLAELEAAFAKSHYPDIYCREELARTTKLNEARIQVWFQNRRAKYRKQE
>CG34367_DR
QRRSRTNFTLDQLNELERLFEETHYPDAFMREELSQRLGLSEARVQVWFQNRRAKCRKHE
>CG9876_DR
PRRNRTTFSSAQLTALEKVFERTHYPDAFVREELATKVHLSEARVQVWFQNRRAKFRRNE
>crx_DA
QRRERTTFTRTQLDILEALFTKTRYPDIFMREEVALKINLPESRVQVWFKNRRAKCRQQQ
>CRX_HU
QRRERTTFTRSQLEELEALFAKTQYPDVYAREEVALKINLPESRVQVWFKNRRAKCRQQR
>CRX_MO
QRRERTTFTRSQLEELEALFAKTQYPDVYAREEVALKINLPESRVQVWFKNRRAKCRQQR
>DMBX1_HU
QRRSRTAFTAQQLEALEKTFQKTHYPDVVMRERLAMCTNLPEARVQVWFKNRRAKFRKKQ
>DMBX1_MO
QRRSRTAFTAQQLEALEKTFQKTHYPDVVMRERLAMCTNLPEARVQVWFKNRRAKFRKKQ
>DMBX1a_DA
QRRSRTAFTAQQLEALEKTFQKTHYPDVVMRERLAMCTNLPEARVQVWFKNRRAKFRKKQ
>DRGX_DA
QRRNRTTFTLQQLEALEAVFAQTHYPDVFTREELAMKINLTEARVQVWFQNRRAKWRKTE
>DRGX_DR
QRRNRTTFTLQQLEELETAFAQTHYPDVFTREDLAMKINLTEARVQVWFQNRRAKWRKAE
>DRGX_HU
QRRNRTTFTLQQLEALEAVFAQTHYPDVFTREELAMKINLTEARVQVWFQNRRAKWRKTE
>DRGX_MO
QRRNRTTFTLQQLEALEAVFAQTHYPDVFTREELAMKINLTEARVQVWFQNRRAKWRKTE
>DU4L2_HU
GRRRRLVWTPSQSEALRACFERNPYPGIATRERLAQAIGIPEPRVQIWFQNERSRQLRQH
>DUX4_2_HU
```

GRRRRLVWTPSQSEALRACFERNPYPGIATRERLAQAIGIPEPRVQIWFQNERSRQLRQH
>DUX4_1_HU
GRRKRTAVTGSQTALLLRAFEKDRFPGIAAREELARETGLPESRIQIWFQNRRARHPGQG
>DUXA_2_HU
HRRCRTKFTEEQLKILINTFNQKPYPGYATKQKLALEINTEESRIQIWFQNRRARHGFQK
>DUXA_1_HU
ARRCRTTYSASQLHTLIKAFMKNPYPGIDSREELAKEIGVPESRVQIWFQNRRSRLLLQR
>ey_DR
LQRNRTSFTNDQIDSLEKEFERTHYPDVFARERLAGKIGLPEARIQVWFSNRRAKWRREE
>eyg_DR
FRRNRTTFSPEQLEELEKEFDKSHYPCVSTRERLSSRTSLSEARVQVWFSNRRAKWRRHQ
>gsb_DR
QRRSRTTFSNDQIDALERIFARTQYPDVYTREELAQSTGLTEARVQVWFSNRRARLRKQL
>GSC_DA
KRRHRTIFTDEQLEALENLFQETKYPDVGTREQLARKVHLREEKVEVWFKNRRAKWRRQK
>GSC_DR
KRRHRTIFTEEQLEQLEATFDKTHYPDVVLREQLALKVDLKEERVEVWFKNRRAKWRKQK
>GSC_HU
KRRHRTIFTDEQLEALENLFQETKYPDVGTREQLARKVHLREEKVEVWFKNRRAKWRRQK
>GSC_MO
KRRHRTIFTDEQLEALENLFQETKYPDVGTREQLARKVHLREEKVEVWFKNRRAKWRRQK
>GSC2_HU
TRRHRTIFSEEQLQALEALFVQNQYPDVSTRERLAGRIRLREERVEVWFKNRRAKWRHQK
>GSC2_MO
TRRHRTIFSEEQLQALEALFVQNQYPDVGTRERLAVRIRLREERVEVWFKNRRAKWRHQK
>HBN_DR
VRRSRTTFTTFQLHQLERAFEKTQYPDVFTREDLAMRLDLSEARVQVWFQNRRAKWRKRE
>HESX1_DA
GRRPRTAFSSVQIKILESVFQVNSYPGIDIREELAKKLHLDEDRIQIWFQNRRAKLKRSH
>HESX1_HU
GRRPRTAFTQNQIEVLENVFRVNCYPGIDIREDLAQKLNLEEDRIQIWFQNRRAKLKRSH
>HESX1_MO
GRRPRTAFTQNQVEVLENVFRVNCYPGIDIREDLAQKLNLEEDRIQIWFQNRRAKMKRSR
>XP_002159617.3_DENV_036790
MRRVRTTFSLEQRRALEDAFEKTPYPDAEQREEISIQCDLPEPRVQVWFSNKRAKLRRQD
>XP_002160848.1
QRRYRTTFTQFQLDELERAFDKTHYPDVFMREELAVRVHLTEARVQVWFQNRRAKWRKRE
>XP_002161581.1
QRRERTTFTKSQLEILEELFLKTHYPDIFMREEAARKINLPESRVQVWFKNRRAKHRQKA
>XP_002162903_DENV_003026
QRRNRTTFSKQQLMELELTFQKKHYPDINTREALAEQIGITEARIQVWFQNRRAKWRKLS
>XP_002168027.1_DENV_045465
KRRYRTTFTTHQLDELERVFNRTHYPDIFLREEMAVKLGLTEARIQVWFQNRRAKWRKRN
>XP_004208605.2_DENV_021903
NKRNRTIFSKKQSCVLEDVFKKTHYPDLKCRYEISQQTKLAENRIQVWFQNRRAKWRRTE
>XP_012556935.1_DENV_099801
RIRMRTNFSPWQLEEELETAFKKSYYPDVYTREVLALKLEIAESRIQVWFQNRRAKWRKAE
>XP_012557153.1
QRRNRTTFSKQQLMELELTFQKKHYPDINTREALAEQIGITEARIQVWFQNRRAKWRKLS
>XP_012558124.1_DENV_044489
LRRNRTTFTTYQLHQLERSFDKTQYPDVFTRENLALKLDLSEARVQVWFQNRRAKWRKRE
>XP_012559896.1_DENV_052386
QRRERTTFTKFQLEILDNLFKETKYPDVFLREDVARRISLPESRVQVWFKNRRAKHRQKS
>XP_012566347.1
HRRVRTAFTHHQLTTLERTFETSHYPDVVLRERLASFTGLAESRIQVWFKNRRAKYRKHQ
>DENV_011065_XP_002164986.2
IRRQRTHFTVTQLHRLETCFARNRYPDMAMREDIAQWCSLTESRVRIWFKNRRAKWRKKE
>DENV_022238_XP_012562202.1
HRRVRTAFTHHQLTTLERTFETSHYPDVVLRERLASFTGLAESRIQVWFKNRRAKYRKHQ
>DENV_024188_XP_012566844.1
GKKTRQCFSTTQVEQLEKLFNEINYPDAYTRQMLAKKMKVSETRIQIWCQNRRAKIRRQR

>DENV_026119_XP_002164410.3
QKRHRTRFTQTQLNELERYFNKTHYPDIFVREELAMRIDLTESRVQVWFQNRRAKWKKRR
>DENV_027852_XP_004207550.1
GRRPRTSITLCQRDFLECEFQKERYPTLLYINKLSSKINLSQYVIKVWFQNRRAKYRKEG
>DENV_043831_XP_002165604.2
KRRHRTIFSDEQLNVLERLFNKTHYPDVIVREEIAGIINLTEEKVEVWFKNRRARWRKQK
>DEN_079506_XP_002157088.1
RRRERTTFTKAQLDVLEDMFGKTMYPDVFMREEVAKKINLAEARVQVWFKNRRAKFRRSR
>DENV_110060_XP_002163599.1
QKRHRTRFNPSQLNELERYFNKTHYPDIFVREELALRIGLTESRVQVWFQNRRAKWKKRK
>DENV_130791_XP_012554469.1
QRRERTTFTKSQLEILEELFLKTHYPDIFMREEAARKINLPESRVQVWFKNRRAKHRQKA
>DENV_177203
QRRNRTTFSKQQLMELELTFQKKHYPDINTREALAEQIGITEARIQVWFQNRRAKWRKLS
>DENV_189615_XP_002160816.1
QRRYRTTFTQFQLDELERAFDKTHYPDVFMREELAVRVHLTEARVQVWFQNRRAKWRKRE
>DENV_191873
QRRYRTTFTQFQLDELERAFDKTHYPDVFMREELAVRVHLTEARVQVWFQNRRAKWRKRE
>HyHox1
AFRKRCSFGHSKIIELEKEFKYNKYLSRDRRVEFARNLELSESQIKIWFQNRRMKQKKEQ
>ISX_HU
KRRVRTTFTTEQLHELEKIFHFTHYPDVHIRSQLAARINLPEARVQIWFQNQRAKWRKQE
>ISX_MO
KRRVRTTFTTEQLQELEKLFHFTHYPDIHVRSQLASRINLPEARVQIWFQNQRAKWRKQE
>MIXL1_DA
HRRKRTNFTQQQIDVLEKVYLDTKYPDIYLREKLEALTGLPESRIQVWFQNRRAKSRRQV
>MIXL1_HU
QRRKRTSFSAEQLQLLELVFRRTRYPDIHLRERLAALTLLPESRIQVWFQNRRAKSRRQS
>MIXL1_MO
QRRKRTSFSSEQLQLLELVFRQTMYPDIHLRERLAALTLLPESRIQVWFQNRRAKSRRQS
>MXTX1_DA
SRRKRTSFSKEHVELLRATFETDPYPGISLRESLSQTTGLPESRIQVWFQNRRARTLKCK
>MXTX2_DA
GRRKRTSFTKEHLELLKMAFNVDPYPGISVRESLSQATGLPESRIQVWFQNKRARTLKNR
>NOBOX_HU
RKKTRTLYRSDQLEELEKIFQEDHYPDSDKRREIAQTVGVTPQRIMVWFQNRRAKWRKME
>NOBOX_MO
RKKTRTLYRSDQLEELERIFQEDHYPDSDKRHEISQMVGVTPQRIMVWFQNRRAKWRKVE
>NvCART1
KRRNRTTFTAYQLEEMERVFQKTHYPDVYTREQLALRCALTEARVQVWFQNRRAKWRKRE
>NVDMBXa
IRRTRTTFNQFQLDTLERAFSRTHYPDVLLREQLAVYTNLPESRIQVWFKNRRAKYRKSK
>NVDMBXb
SRRTRTAFTHQQLTALEKVFSKTHYPDVEVREQLATSTNLQEARIQVWFKNRRAKYRKDQ
>NVDMBXc
PRRMRTCFTPYQLQVLENTFCNTHYPDVMLREQLASYVNLPEARIQVWFKNRRAKHRKND
>NVDMBXf
HRRTRTAFTHQQLQILESTFSKTHYPDVVMREQLAAYINIPESRIQVWFKNRRAKYRKQV
>NVDUXa
HRRQRTFFSKEQTVILEGAFQYERFPGIQIREKLARELDIDESRIQVWFQNRRSRQNRQK
>NVDUXb
HRRQRTFFSKEQTVILEGAFQYERFPGIQIREKLARELDIDESRIQVWFQNRRSRQNRQK
>NVDUXc
HRRQRTFFSKEQTVILEGAFQYERFPGIQIREKLARELDIDESRIQVWFQNRRSRQNRQK
>NVGSC
KRRHRTIFTEEQLELLETTFQKTHYPDVLLREELAMKVDLKEERVEVWFKNRRAKWRKQK
>NVHBN
VRRSRTTFTTYQLHQLERAFEKTQYPDVFTREELALRLDLSEARVQVWFQNRRAKWRKRE
>NVHD014
QRRSRTKFTSKQVDELEKAFLKTQYPDVYTREELAQRLNLTEARVQVWFSNRRARLRKKK
>NVHD031

RRRCRTVFSTEQLAILEEGFQKQHFPDNKLRQAIATRAGLPEDRVQVWFQNRRAKEKRLL
>NVHD037
KRRHRTTFSKLQLDSLEEAFSRSQYPDVFTREQLAKRINLNEARVQVWFQNRRAKHRKQE
>NVHD052
IKRKRRNLTKRQQKILETAYSTIKYPTIEDRQRLETSTQLSEDRIQVWFQNRRAKDRRLQ
>NVHD058
HRRKRTTFTHDQLQLMEAYFHNNRYPGIEQREGLAEKIQVSESRLQVWFQNRRSKWRKHQ
>NVHD074
QRRNRTKFTAYQLEQLEDAYQKAKYPDVQARETLAQRLGVAESRVQVWFSNRRSKGKRKE
>NVHD100
QRRYRTTFTSYQLEELERAFAKTHYPDVFTREALAVKIDLTEARVQVWFQNRRAKWRKRE
>NVHD101
PRRSRTRFTVSQTDELERAFRKTHYPDIYAREELAQRLGLSEARVQVWFSNRRARLRKER
>NVHD146
GKRYRATFDKAQIFQMERVFLLNHYPDVAARSELSRRTGLSESQVQIWFQNRRAKWRKQQ
>NVHD148
SRRQRTNFTDEQIEKLEKVFEKTHYPDVFTREELAQQVNLSEARIQVWYSNRRAKWRKEG
>NVOTP
QKRHRTRFTPAQLNELERCFARTHYPDVFMREELAARIGLTESRVQVWFQNRRAKWKKRK
>NVOTXa
QRRERTTFTKNQLEVLEELFAKTRYPDIFMREEVAIKINLPESRVQVWFKNRRAKTRQLE
>NVOTXb
QRRERTTFTKNQLEILEELFAKTRYPDIFMREEVAIKINLPESRVQVWFKNRRAKARQQA
>NVOTXc
QRRERTTFTKNQLEILEELFAKTRYPDIFMREEVAIKINLPESRVQVWFKNRRAKLRQLS
>NVPAX4/6
LRRNRTTFTPDQLEMLEKEFEKSHYPDVATREELANKIDMSEARVQVWFSNRRAKWRRHQ
>NVPTX
QRRQRTHFTSFQLQQLEGTFGRNRYPDMQMREEIALYTNLTEARVRVWFKNRRAKWRKKE
>NVREPO
KTRYRTTFSQYQIEELERAFDKAPYPDVFAREELAAKLGLTEARIQVWFQNRRAKWRKRE
>NVRX
LRRNRTTFTTFQLHELERAFEKSHYPDVYTREELALKISLPEVRVQVWFQNRRAKWRRQE
>NVUNC4
RMRVRTNFSPWQLEELEHAFETTHYPDVFMREALAMRLDLTEARVQVWFQNRRAKWRKRE
>oc_DR
QRRERTTFTRAQLDVLEALFGKTRYPDIFMREEVALKINLPESRVQVWFKNRRAKCRQQL
>OdsH_DR
KRRGRTNFNSWQLRELERVFQGSHYPDIFMREALATKLDLMEGRIAVWFQNRRAKWRKQE
>OTP_DR
QKRHRTRFTPAQLNELERCFSKTHYPDIFMREEIAMRIGLTESRVQVWFQNRRAKWKKRK
>OTP_HU
QKRHRTRFTPAQLNELERSFAKTHYPDIFMREELALRIGLTESRVQVWFQNRRAKWKKRK
>OTP_MO
QKRHRTRFTPAQLNELERSFAKTHYPDIFMREELALRIGLTESRVQVWFQNRRAKWKKRK
>OTPa_DA
QKRHRTRFTPAQLNELERSFAKTHYPDIFMREELALRIGLTESRVQVWFQNRRAKWKKRK
>OTPb_DA
QKRHRTRFTPAQLNELERSFAKTHYPDIFMREELALRIGLTESRVQVWFQNRRAKWKKRK
>OTX1_HU
QRRERTTFTRSQLDVLEALFAKTRYPDIFMREEVALKINLPESRVQVWFKNRRAKCRQQQ
>OTX1_MO
QRRERTTFTRSQLDVLEALFAKTRYPDIFMREEVALKINLPESRVQVWFKNRRAKCRQQQ
>OTX1a_DA
QRRERTTFTRTQLDILESLFAKTRYPDIFMREEVALKINLPESRVQVWFKNRRAKCRQQQ
>OTX1b_DA
QRRERTTFTRSQLDILEALFAKTRYPDIFMREEVALKINLPESRVQVWFKNRRAKCRQQQ
>OTX2_DA
QRRERTTFTRAQLDVLEALFAKTRYPDIFMREEVALKINLPESRVQVWFKNRRAKCRQQQ
>OTX2_HU
QRRERTTFTRAQLDVLEALFAKTRYPDIFMREEVALKINLPESRVQVWFKNRRAKCRQQQ

>OTX2_MO
QRRERTTFTRAQLDVLEALFAKTRYPDIFMREEVALKINLPESRVQVWFKNRRAKCRQQQ
>OTX5_DA
QRRERTTFTRAQLDVLEALFSKTRYPDIFMREEVALKINLPESRVQVWFKNRRAKCRQQQ
>PAX3_HU
QRRSRTTFTAEQLEELERAFERTHYPDIYTREELAQRAKLTEARVQVWFSNRRARWRKQA
>PAX3_MO
QRRSRTTFTAEQLEELERAFERTHYPDIYTREELAQRAKLTEARVQVWFSNRRARWRKQA
>PAX3a_DA
QRRSRTTFTAEQLEELERAFERTHYPDIYTREELAQRAKLTEARVQVWFSNRRARWRKQA
>PAX3b_DA
QRRSRTTFTADQLEELERAFERTHYPDIYTREELAQRAKLTEARVQVWFSNRRARWRKQA
>PAX4_DA
AHRSRTAFTADQSGRLEKEFTCGLYPDLLTREKLAEETNLSQDTIKVWFSNRRARMRRER
>PAX4_HU
GHRNRTIFSPSQAEALEKEFQRGQYPDSVARGKLATATSLPEDTVRVWFSNRRAKWRRQE
>PAX4_MO
SHRNRTIFSPGQAEALEKEFQRGQYPDSVARGKLAAATSLPEDTVRVWFSNRRAKWRRQE
>PAX6_HU
LQRNRTSFTQEQIEALEKEFERTHYPDVFARERLAAKIDLPEARIQVWFSNRRAKWRREE
>PAX6_MO
LQRNRTSFTQEQIEALEKEFERTHYPDVFARERLAAKIDLPEARIQVWFSNRRAKWRREE
>PAX6a_DA
LQRNRTSFTQEQIEALEKEFERTHYPDVFARERLAAKIDLPEARIQVWFSNRRAKWRREE
>PAX7_HU
QRRSRTTFTAEQLEELEKAFERTHYPDIYTREELAQRTKLTEARVQVWFSNRRARWRKQA
>PAX7_MO
QRRSRTTFTAEQLEELEKAFERTHYPDIYTREELAQRTKLTEARVQVWFSNRRARWRKQA
>PAX7a_DA
QRRSRTTFTAEQLEELEKAFERTHYPDIYTREELAQRTKLTEARVQVWFSNRRARWRKQA
>PHDP_DR
QRRIRTTFTSNQLNELEKIFLETHYPDIYTREEIASKLHLTEARVQVWFQNRRAKFRKQE
>PHOX2a_DA
QRRIRTTFTSSQLKELERVFAETHYPDIYTREELALKIDLTEARVQVWFQNRRAKFRKQE
>PHOX2b_DA
QRRIRTTFTSAQLKELERVFAETHYPDIYTREELALKIDLTEARVQVWFQNRRAKFRKQE
>PHX2A_HU
QRRIRTTFTSAQLKELERVFAETHYPDIYTREELALKIDLTEARVQVWFQNRRAKFRKQE
>PHX2A_MO
QRRIRTTFTSAQLKELERVFAETHYPDIYTREELALKIDLTEARVQVWFQNRRAKFRKQE
>PHX2B_HU
QRRIRTTFTSAQLKELERVFAETHYPDIYTREELALKIDLTEARVQVWFQNRRAKFRKQE
>PHX2B_MO
QRRIRTTFTSAQLKELERVFAETHYPDIYTREELALKIDLTEARVQVWFQNRRAKFRKQE
>PITX1_DA
QRRQRTHFTSQQLQELEATFQRNRYPDMSTREEIAVWTNLTEARVRVWFKNRRAKWRKRE
>PITX1_HU
QRRQRTHFTSQQLQELEATFQRNRYPDMSMREEIAVWTNLTEPRVRVWFKNRRAKWRKRE
>PITX1_MO
QRRQRTHFTSQQLQELEATFQRNRYPDMSMREEIAVWTNLTEPRVRVWFKNRRAKWRKRE
>PITX2_DA
QRRQRTHFTSQQLQELEATFQRNRYPDMSTREEIAVWTNLTEARVRVWFKNRRAKWRKRE
>PITX2_HU
QRRQRTHFTSQQLQELEATFQRNRYPDMSTREEIAVWTNLTEARVRVWFKNRRAKWRKRE
>PITX2_MO
QRRQRTHFTSQQLQELEATFQRNRYPDMSTREEIAVWTNLTEARVRVWFKNRRAKWRKRE
>PITX3_DA
QRRQRTHFTSQQLQELEATFQRNRYPDMSTREEIAVWTNLTEARVRVWFKNRRAKWRKRE
>PITX3_HU
QRRQRTHFTSQQLQELEATFQRNRYPDMSTREEIAVWTNLTEARVRVWFKNRRAKWRKRE
>PITX3_MO

QRRQRTHFTSQQLQELEATFQRNRYPDMSTREEIAVWTNLTEARVRVWFKNRRAKWRKRE
>Pph13_DR
QRRYRTTFNTLQLQELERAFQRTHYPDVFFREELAVRIDLTEARVQVWFQNRRAKWRKQE
>prd_DR
QRRCRTTFSASQLDELERAFERTQYPDIYTREELAQRTNLTEARIQVWFSNRRARLRKQH
>PROP1_DA
RRRHRTTFSNEQLEHLELAFRQNHYPDIYYREELARVTKLNEARIQAWFQNRRAKQRKQD
>PROP1_HU
RRRHRTTFSPVQLEQLESAFGRNQYPDIWARESLARDTGLSEARIQVWFQNRRAKQRKQE
>PROP1_MO
RRRHRTTFNPAQLEQLESAFGRNQYPDIWAREGLAQDTGLSEARIQVWFQNRRAKQRKQE
>PRRX1_HU
QRRNRTTFNSSQLQALERVFERTHYPDAFVREDLARRVNLTEARVQVWFQNRRAKFRRNE
>PRRX1_MO
QRRNRTTFNSSQLQALERVFERTHYPDAFVREDLARRVNLTEARVQVWFQNRRAKFRRNE
>PRRX1a_DA
QRRNRTTFNSSQLQALERVFERTHYPDAFVREDLARRVNLTEARVQVWFQNRRAKFRRNE
>PRRX2_HU
QRRNRTTFNSSQLQALERVFERTHYPDAFVREELARRVNLSEARVQVWFQNRRAKFRRNE
>PRRX2_MO
QRRNRTTFNSSQLQALERVFERTHYPDAFVREELARRVNLSEARVQVWFQNRRAKFRRNE
>PTX1_DR
QRRQRTHFTSQQLQELEHTFSRNRYPDMSTREEIAMWTNLTEARVRVWFKNRRAKWRKRE
>RAX2_HU
HRRNRTTFTTYQLHQLERAFEASHYPDVYSREELAAKVHLPEVRVQVWFQNRRAKWRRQE
>REPO_DR
KKKTRTTFTAYQLEELERAFERAPYPDVFAREELAIKLNLSESRVQVWFQNRRAKWRKHE
>RX_DR
HRRNRTTFTTYQLHELERAFEKSHYPDVYSREELAMKVNLPEVRVQVWFQNRRAKWRRQE
>RX_HU
HRRNRTTFTTYQLHELERAFEKSHYPDVYSREELAGKVNLPEVRVQVWFQNRRAKWRRQE
>RX_MO
HRRNRTTFTTYQLHELERAFEKSHYPDVYSREELAGKVNLPEVRVQVWFQNRRAKWRRQE
>RX1_DA
HRRNRTTFTTYQLHELERAFEKSHYPDVYSREELAMKVNLPEVRVQVWFQNRRAKWRRQE
>RX2_DA
HRRNRTTFTTYQLHELERAFEKSHYPDVYSREELAMKVNLPEVRVQVWFQNRRAKWRRQE
>RX3_DA
HRRNRTTFTTFQLHELERAFEKSHYPDVYSREELALKVNLPEVRVQVWFQNRRAKWRRQE
>SEBOX_DA
RKRKRTIFSRAQLSELERAFMITPYPDITLRERLAALTLLPESKIQVWFQNRRARSMKSK
>SEBOX_HU
HRRKRTTFSKGQLLELERAFAAWPYPNISTHEHLAWVTCLPEAKVQVWFQKRWAKIIKNR
>SEBOX_MO
HRRKRTTFSVGQLVELERVFAARPYPDISTREHLAQVTHLPEAKIQVWFQNRRAKRIKDR
>SHOX_DA
QRRSRTNFTLEQLNELERLFDETHYPDAFMREELSQRLGLSEARVQVWFQNRRAKCRKQE
>SHOX_HU
QRRSRTNFTLEQLNELERLFDETHYPDAFMREELSQRLGLSEARVQVWFQNRRAKCRKQE
>SHOX2_DA
QRRSRTNFTLEQLNELERLFDETHYPDAFMREELSQRLGLSEARVQVWFQNRRAKCRKQE
>SHOX2_HU
QRRSRTNFTLEQLNELERLFDETHYPDAFMREELSQRLGLSEARVQVWFQNRRAKCRKQE
>SHOX2_MO
QRRSRTNFTLEQLNELERLFDETHYPDAFMREELSQRLGLSEARVQVWFQNRRAKCRKQE
>SP83252
KRRYRTTFTSFQLRELEKAFERTHYPDVFTREDLANRVELTEARVQVWFQNRRAKWRKKE
>SP86105
QRRHRTNFTSHQLEELEKAFEKTRYPDVFMREELAMKISLTEARVQVWFQNRRAKWRKAE
>SP87530
SKRNRTTFTAHQLDELEMIFRQTHYPDVLLREKLAQRIGLPESRVQVWFQNRRAKWRKRE

```
>SP87700
QRRFRTTFTSYQLQELEAAFAKTHYPDVFMREDLALRINLTEARVQVWFQNRRAKWRRAQ
>SP89232
PKRTRTAYSNSQLDQLELIFATTHYPDVFTREDLSRRLGIREDRIQVWFQNRRARFRKQE
>SP91658
PKRTRTAYSNSQLDQLELIFATTHYPDVFTREDLSRRLGIREDRIQVWFQNRRARFRKQE
>toe_DR
FRRNRTTFSPEQLDELEKEFDKSHYPCVNTREKLAARTALSEARVQ------TSEMAAPP
>toy_DR
LQRNRTSFSNEQIDSLEKEFERTHYPDVFARERLADKIGLPE----VWFSNRRAKWRREE
>UNC4_DR
RRRSRTNFNSWQLEELERAFSASHYPDIFMREALAMRLDLKESRVAVWFQNRRAKVRKRE
>UNC4_HU
RRRTRTNFTGWQLEELEKAFNESHYPDVFMREALALRLDLVESRVQVWFQNRRAKWRKKE
>UNC4_MO
RRRTRTNFTGWQLEELEKAFNESHYPDVFMREALALRLDLVESRVQVWFQNRRAKWRKKE
>UNCX_DA
RRRTRTNFTGWQLEELEKAFNESHYPDVFMREALALRLDLIESRVQVWFQNRRAKWRKKE
>UNCX4_DA
RRRTRTNFTGWQLEELEKAFNESHYPDVFMREALALRLDLVESRVQVWFQNRRAKWRKKE
>VSX1_DA
KRRHRTVFTSHQLEELEKAFNEAHYPDVYAREMLAMKTELPEDRIQVWFQNRRAKWRKRE
>VSX1_DR
RRHGRTIFTSSQLEELEKAFKEAHYPDVSARELLSMKTGLAEDRIQVWYQNRRAKWRKTE
>VSX1_HU
KRRHRTVFTAHQLEELEKAFSEAHYPDVYAREMLAVKTELPEDRIQVWFQNRRAKWRKRE
>VSX1_MO
KRRHRTVFTAHQLEELEKAFGEAHYPDVYAREMLAAKTELPEDRIQVWFQNRRAKWRKRE
>VSX2_DA
KRRHRTIFTSYQLEELEKAFNEAHYPDVYAREMLAMKTELPEDRIQVWFQNRRAKWRKRE
>VSX2_DR
RRHSRTIFTSYQLEKLEEAFKEAHYPDVYAREMLSLKTELPEDRIQVWFQNRRAKWRKTE
>VSX2_HU
KRRHRTIFTSYQLEELEKAFNEAHYPDVYAREMLAMKTELPEDRIQVWFQNRRAKWRKRE
>VSX2_MO
KRRHRTIFTSYQLEELEKAFNEAHYPDVYAREMLAMKTELPEDRIQVWFQNRRAKWRKRE
```

Finding orthologs using RBH (reciprocal BAST hits) in planaria and Xenopus

1) **Command lines used for generating the RBH data**

$makeblastdb -in ./blast_db/hydra_hybrid_protein.fasta -dbtype prot -out ./blast_db/hydra_hybrid_protein
$makeblastdb -in ./blast_db/planaria_longest_orfs_mod.pep -dbtype prot -out ./blast_db/planaria_longest_orfs_mod
$makeblastdb -in ./blast_db/XL9_1_v20161019_primaryTranscripts_pep.fa -dbtype prot -out ./blast_db/XL9_1_v20161019_primaryTranscripts_pep
#./reverse_blast.sh
$blastp -num_threads 12 -query ./blast_db/hydra_hybrid_protein.fasta -db ./blast_db/planaria_longest_orfs_mod -out ./RBH/hydra_vs_planaria -evalue 0.01 -outfmt 7 -max_target_seqs 1
$blastp -num_threads 12 -query ./blast_db/planaria_longest_orfs_mod.pep -db ./blast_db/hydra_hybrid_protein -out ./RBH/planaria_vs_hydra -evalue 0.01 -outfmt 7 -max_target_seqs 1
$blastp -num_threads 12 -query ./blast_db/hydra_hybrid_protein.fasta -db ./blast_db/XL9_1_v20161019_primaryTranscripts_pep -out ./RBH/hydra_vs_xenopus -evalue 0.01 -outfmt 7 -max_target_seqs 1

```
$blastp -num_threads 12 -query
./blast_db/XL9_1_v20161019_primaryTranscripts_pep.fa -db
./blast_db/hydra_hybrid_protein -out ./RBH/xenopus_vs_hydra -evalue 0.01 -outfmt
7 -max_target_seqs 1

$awk '/^DENV|^XP/' ./RBH/hydra_vs_planaria|awk '{if($3>=30) print$0}'|awk
'{print$1,$2}'|sort -u  > ./RBH/hydra_planaria_table
$awk '/^tr5/' ./RBH/planaria_vs_hydra |awk '{if($3>=30) print$0}'|awk
'{print$1,$2}'|sort -u > ./RBH/planaria_hydra_table
$awk '/^DENV|^XP/' ./RBH/hydra_vs_xenopus|awk '{if($3>=30) print$0}'|awk
'{print$1,$2}'|sort -u > ./RBH/hydra_xenopus_table
$awk '/^gnl/' ./RBH/xenopus_vs_hydra|awk '{if($3>=30) print$0}'|awk
'{print$1,$2}'|sort -u > ./RBH/xenopus_hydra_table
$awk 'NR==FNR{a[$1,$2];next} ($2,$1) in a' ./RBH/planaria_hydra_table
./RBH/hydra_planaria_table > ./RBH/hydra_planaria_rbh
$awk 'NR==FNR{a[$1,$2];next} ($2,$1) in a' ./RBH/xenopus_hydra_table
./RBH/hydra_xenopus_table > ./RBH/hydra_xenopus_rbh
```

2) Protein IDs of genes upregulated upon knockdown of β-catenin with their orthologs (based on RBH-reciprocal BLAST hits) in planaria

```
DENV_002883_XP_002166177.1    tr5_comp8157_c0_seq1_m_4534
DENV_003721 tr5_comp1472_c0_seq1_m_2764
DENV_008342_NP_001267778.1    tr5_comp2989_c0_seq1_m_10262
DENV_017300_XP_002166749.1    tr5_comp18327_c0_seq1_m_12529
DENV_031968_XP_012554474.1    tr5_comp11238_c0_seq1_m_12911
DENV_055772_XP_002157026.1    tr5_comp17606_c0_seq1_m_12097
DENV_057854 tr5_comp21026_c0_seq1_m_17528
DENV_066788_XP_002163988.1    tr5_comp2749_c0_seq1_m_8218
DENV_076867_XP_012560246.1    tr5_comp316_c0_seq1_m_8874
DENV_097762_XP_012565729.1    tr5_comp24701_c0_seq1_m_17136
DENV_097978_XP_012563379.1    tr5_comp7445_c0_seq1_m_1572
DENV_098962_XP_004208490.1    tr5_comp3340_c0_seq1_m_5018
DENV_127367_XP_002163146.3    tr5_comp7567_c0_seq1_m_4070
DENV_134105 tr5_comp150_c0_seq1_m_450
DENV_163354_XP_012555860.1    tr5_comp2868_c0_seq1_m_10311
XP_002166590.1_DENV_075186    tr5_comp19681_c0_seq1_m_12643
XP_002168605.3_DENV_067560    tr5_comp7514_c0_seq1_m_4569
XP_004208605.2_DENV_021903    tr5_comp39772_c0_seq1_m_18769
XP_012553532.1_DENV_100559    tr5_comp4330_c0_seq1_m_551
XP_012554603.1_DENV_178355    tr5_comp6149_c0_seq1_m_7319
XP_012555020.1_DENV_049455    tr5_comp1616_c0_seq1_m_8832
XP_012555758.1_DENV_013271    tr5_comp20054_c0_seq1_m_17081
XP_012555832.1_DENV_170349    tr5_comp7416_c0_seq1_m_6801
XP_012556963.1_DENV_068869    tr5_comp11764_c0_seq1_m_12451
XP_012558898.1    tr5_comp5422_c0_seq1_m_2396
XP_012559357.1_DENV_023644    tr5_comp14634_c0_seq1_m_14972
XP_012561605.1_DENV_008711    tr5_comp3700_c0_seq1_m_5956
XP_012564494.1_DENV_176993    tr5_comp4647_c0_seq1_m_5548
XP_012565081.1    tr5_comp803_c0_seq1_m_10400
XP_012565777.1    tr5_comp16411_c0_seq1_m_10830
XP_012566719.1_DENV_174502    tr5_comp8109_c0_seq1_m_47
```

3) Protein IDs of genes downregulated upon knockdown of β-catenin with their orthologs (based on RBH-reciprocal BLAST hits) in planaria

```
DENV_012348_XP_012553992.1    tr5_comp5362_c0_seq1_m_7568
DENV_029380_XP_012555637.1    tr5_comp13078_c0_seq1_m_14058
```

DENV_051689 tr5_comp4080_c0_seq1_m_396
DENV_056127_XP_004207355.2   tr5_comp12334_c0_seq1_m_13170
DENV_056573_XP_012566931.1   tr5_comp14231_c0_seq1_m_14133
DENV_060128_XP_012553600.1   tr5_comp9437_c0_seq1_m_8970
DENV_073439 tr5_comp12477_c0_seq1_m_13438
DENV_078983_XP_004206814.1   tr5_comp3625_c0_seq1_m_4072
DENV_085512_XP_002159764.2   tr5_comp13566_c0_seq1_m_10678
DENV_089748_NP_001267780.1   tr5_comp2508_c0_seq1_m_7772
DENV_091231_XP_004206634.1   tr5_comp8204_c0_seq1_m_4187
DENV_102767_XP_012555741.1   tr5_comp9535_c0_seq1_m_8607
DENV_103840_XP_012555807.1   tr5_comp19981_c0_seq1_m_18049
DENV_110060_XP_002163599.1   tr5_comp27318_c0_seq1_m_17771
DENV_139448_XP_002160774.1   tr5_comp4102_c0_seq1_m_1317
DENV_144578 tr5_comp7938_c0_seq1_m_8690
DENV_164073_XP_002162811.3   tr5_comp20320_c0_seq1_m_18500
DENV_173944_XP_002155943.3   tr5_comp6293_c0_seq1_m_6003
DENV_191448_XP_002164795.3   tr5_comp125_c0_seq1_m_10183
DENV_198081_XP_002155049.1   tr5_comp4681_c0_seq1_m_7022
DENV_198433 tr5_comp637_c0_seq1_m_5451
XP_002153848.3_DENV_045673   tr5_comp24446_c0_seq1_m_17218
XP_002159974.1_DENV_049843   tr5_comp6898_c0_seq1_m_10014
XP_002163296.3_DENV_106178   tr5_comp11071_c0_seq1_m_10577
XP_002163691.2_DENV_170250   tr5_comp36486_c0_seq1_m_19493
XP_002164046.1   tr5_comp1487_c0_seq1_m_9540
XP_002165472.2   tr5_comp4801_c0_seq1_m_3100
XP_002165493.1_DENV_051715   tr5_comp10336_c0_seq1_m_13562
XP_002166171.3_DENV_197934   tr5_comp11025_c0_seq1_m_15317
XP_002168125.2_DENV_176500   tr5_comp5964_c0_seq1_m_8909
XP_004208612.2_DENV_171524   tr5_comp9177_c0_seq1_m_10244
XP_012554604.1_DENV_101910   tr5_comp32538_c0_seq1_m_19111
XP_012555618.1_DENV_004709   tr5_comp14941_c0_seq1_m_14112
XP_012556297.1_DENV_058222   tr5_comp3789_c0_seq1_m_2565
XP_012556498.1_DENV_200779   tr5_comp4929_c0_seq1_m_8401
XP_012558698.1_DENV_051053   tr5_comp347_c0_seq1_m_457
XP_012559634.1_DENV_168885   tr5_comp3801_c0_seq1_m_2460
XP_012561026.1_DENV_084438   tr5_comp1908_c0_seq1_m_9940
XP_012561260.1   tr5_comp2726_c0_seq1_m_2598
XP_012561295.1_DENV_008382   tr5_comp9858_c0_seq1_m_11192
XP_012562708.1_DENV_062145   tr5_comp9081_c0_seq1_m_2737
XP_012566382.1_DENV_178167   tr5_comp14597_c0_seq1_m_11204

4) Protein IDs of genes upregulated upon knockdown of β-catenin with their orthologs (based on RBH-reciprocal BLAST hits) in Xenopus

DENV_002883_XP_002166177.1   Xelaev18033356m
DENV_008342_NP_001267778.1   Xelaev18011464m
DENV_008531 Xelaev18026968m
DENV_009915_XP_012556947.1   Xelaev18033977m
DENV_010691_XP_012557009.1   Xelaev18041017m
DENV_017300_XP_002166749.1   Xelaev18000174m
DENV_022783_XP_012557245.1   Xelaev18036422m
DENV_023080_XP_002164291.1   Xelaev18025040m
DENV_029253_NP_001274286.1   Xelaev18027357m
DENV_031402_XP_002165888.1   Xelaev18046281m
DENV_031968_XP_012554474.1   Xelaev18018810m
DENV_055772_XP_002157026.1   Xelaev18039153m
DENV_057854 Xelaev18002488m
DENV_066788_XP_002163988.1   Xelaev18030893m
DENV_067234_XP_012566767.1   Xelaev18011720m
DENV_070660_XP_002157611.3   Xelaev18022709m
DENV_097762_XP_012565729.1   Xelaev18017244m
DENV_097978_XP_012563379.1   Xelaev18037770m

DENV_098962_XP_004208490.1   Xelaev18043355m
DENV_127367_XP_002163146.3   Xelaev18011004m
DENV_146956 Xelaev18028129m
DENV_148889_XP_002164528.2   Xelaev18026705m
DENV_157487 Xelaev18038520m
DENV_163354_XP_012555860.1   Xelaev18024895m
DENV_188436_XP_002154928.1   Xelaev18035982m
XP_002154008.1   Xelaev18037744m
XP_002163087.2_DENV_169466   Xelaev18026971m
XP_002166590.1_DENV_075186   Xelaev18033038m
XP_002168605.3_DENV_067560   Xelaev18013914m
XP_004208605.2_DENV_021903   Xelaev18026576m
XP_004211659.2_DENV_037292   Xelaev18018977m
XP_012553451.1   Xelaev18029006m
XP_012553532.1_DENV_100559   Xelaev18044849m
XP_012554388.1_DENV_061537   Xelaev18006883m
XP_012555020.1_DENV_049455   Xelaev18021210m
XP_012555758.1_DENV_013271   Xelaev18021235m
XP_012556963.1_DENV_068869   Xelaev18026122m
XP_012557068.1   Xelaev18003904m
XP_012557269.1   Xelaev18003719m
XP_012558523.1_DENV_090796   Xelaev18009322m
XP_012559357.1_DENV_023644   Xelaev18023669m
XP_012560419.1_DENV_152770   Xelaev18014156m
XP_012561605.1_DENV_008711   Xelaev18045680m
XP_012563378.1_DENV_206654   Xelaev18003717m
XP_012564439.1_DENV_006543   Xelaev18037126m
XP_012564494.1_DENV_176993   Xelaev18015103m
XP_012566923.1_DENV_170123   Xelaev18012891m

5) Protein IDs of genes downregulated upon knockdown of β-catenin with their orthologs (based on RBH-reciprocal BLAST hits) in Xenopus

DENV_008833_XP_012554670.1   Xelaev18026018m
DENV_012348_XP_012553992.1   Xelaev18020051m
DENV_022146_XP_002165751.1   Xelaev18034616m
DENV_027001_XP_002158990.1   Xelaev18018204m
DENV_046948 Xelaev18033688m
DENV_048507_XP_012562827.1   Xelaev18012794m
DENV_056127_XP_004207355.2   Xelaev18020972m
DENV_056573_XP_012566931.1   Xelaev18013013m
DENV_060128_XP_012553600.1   Xelaev18022960m
DENV_069649 Xelaev18012288m
DENV_076720_XP_002158844.1   Xelaev18020482m
DENV_078983_XP_004206814.1   Xelaev18005453m
DENV_085512_XP_002159764.2   Xelaev18034293m
DENV_089748_NP_001267780.1   Xelaev18031149m
DENV_093426_XP_004210083.1   Xelaev18043203m
DENV_098364_XP_012555674.1   Xelaev18039397m
DENV_101803_XP_012565167.1   Xelaev18045463m
DENV_102767_XP_012555741.1   Xelaev18040863m
DENV_109387_XP_012562538.1   Xelaev18011397m
DENV_113734_XP_012556903.1   Xelaev18006583m
DENV_118389_NP_001274292.1   Xelaev18043371m
DENV_128081_XP_012559396.1   Xelaev18005839m
DENV_129246_XP_012560559.1   Xelaev18011685m
DENV_135075_XP_002164912.3   Xelaev18029079m
DENV_139448_XP_002160774.1   Xelaev18002478m
DENV_141689_XP_002158070.2   Xelaev18030633m
DENV_164073_XP_002162811.3   Xelaev18028982m

```
DENV_173944_XP_002155943.3    Xelaev18045581m
DENV_179050_XP_012555495.1    Xelaev18022865m
DENV_183673_XP_002154419.2    Xelaev18005899m
DENV_184349_XP_002157228.2    Xelaev18009169m
DENV_191448_XP_002164795.3    Xelaev18011935m
DENV_192159_XP_012558303.1    Xelaev18040932m
DENV_198081_XP_002155049.1    Xelaev18029658m
DENV_198433 Xelaev18026677m
DENV_203339_XP_012561918.1    Xelaev18041536m
XP_002153848.3_DENV_045673    Xelaev18032986m
XP_002156487.1_DENV_099471    Xelaev18032949m
XP_002159974.1_DENV_049843    Xelaev18019997m
XP_002162975.1_DENV_038595    Xelaev18015825m
XP_002165472.2    Xelaev18029500m
XP_002165493.1_DENV_051715    Xelaev18016633m
XP_002166171.3_DENV_197934    Xelaev18031534m
XP_002167951.2_DENV_065194    Xelaev18025043m
XP_002168125.2_DENV_176500    Xelaev18017736m
XP_004208612.2_DENV_171524    Xelaev18025326m
XP_004211105.1_DENV_034494    Xelaev18017003m
XP_012553964.1    Xelaev18025341m
XP_012554405.1_DENV_105277    Xelaev18021564m
XP_012554629.1_DENV_039347    Xelaev18021990m
XP_012554760.1    Xelaev18013127m
XP_012556498.1_DENV_200779    Xelaev18025960m
XP_012557119.1_DENV_141373    Xelaev18038940m
XP_012558698.1_DENV_051053    Xelaev18032838m
XP_012559409.1_DENV_179707    Xelaev18041001m
XP_012559634.1_DENV_168885    Xelaev18027250m
XP_012560374.1_DENV_179864    Xelaev18005545m
XP_012561260.1    Xelaev18031383m
XP_012561295.1_DENV_008382    Xelaev18031124m
XP_012562678.1_DENV_130362    Xelaev18013693m
XP_012562708.1_DENV_062145    Xelaev18022141m
XP_012564205.1_DENV_168699    Xelaev18034771m
XP_012566382.1_DENV_178167    Xelaev18038200ma
```

Melt curve plots for ChIP qRT-PCR

Melt curve of *Margin* - 1

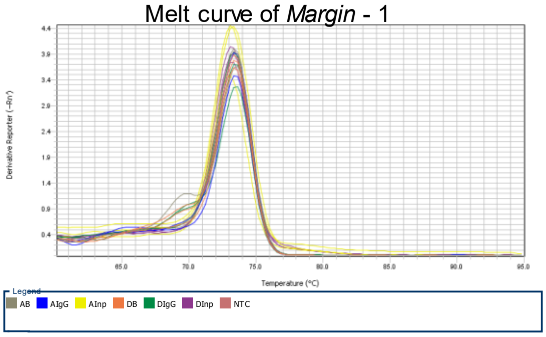

Melt curve of *Margin* - 2

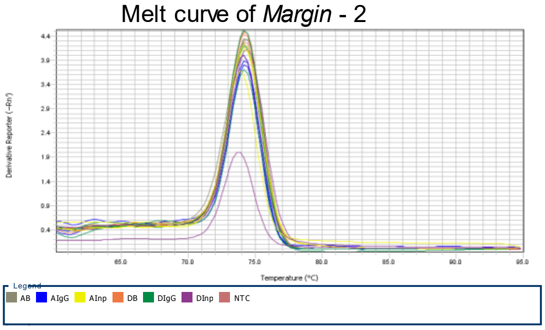

Melt curve of *Margin* - 3

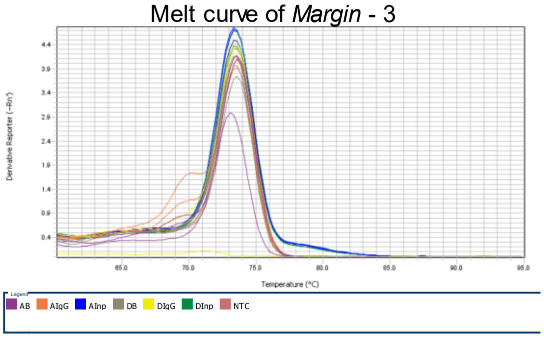

Melt curve of *Margin* - 4

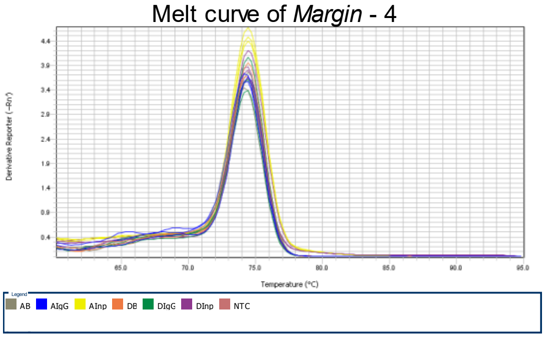

Melt curve of Desert region

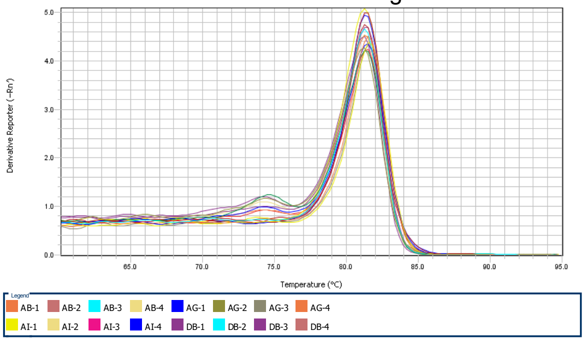

TCF7L2 binding motifs on promoter regions of *HvOtp1*, *HvOtx1* and *Gorget*

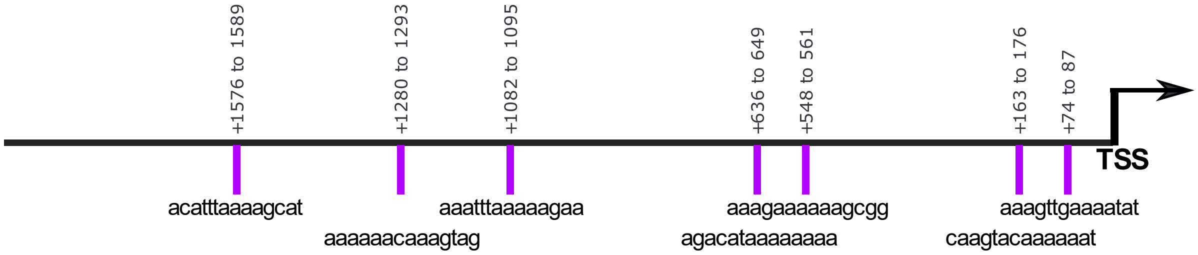

HvOtp1 promoter - NW_004167801.1: 137146-139146

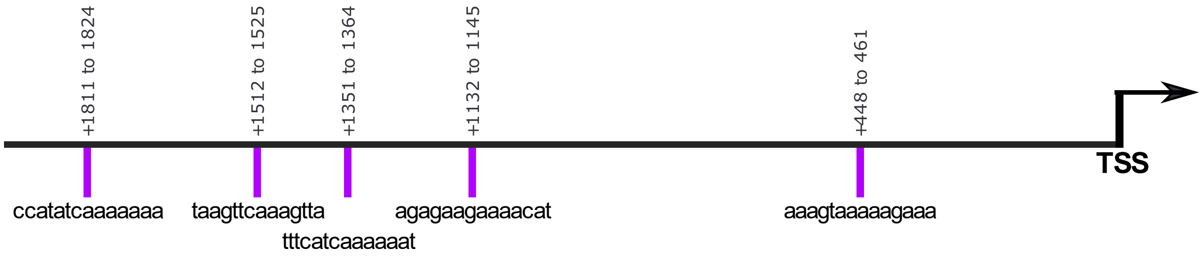

HvOtx1 promoter - NW_004169543.1: 87178-89178

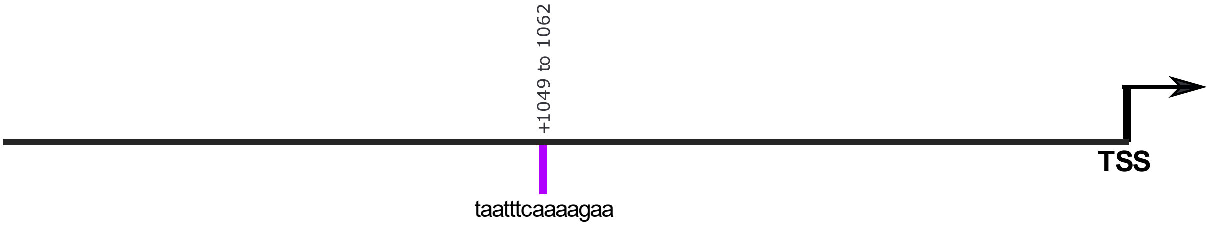

Gorget promoter - NW_004172274.1: 24425-26425

References

1 Siebert, S. *et al.* Stem cell differentiation trajectories in Hydra resolved at single-cell resolution. *Science* **365**, doi:10.1126/science.aav9314 (2019).

2 Wenger, Y., Buzgariu, W., Perruchoud, C., Loichot, G. & Galliot, B. Generic and context-dependent gene modulations during Hydra whole body regeneration. *bioRxiv*, 587147, doi:10.1101/587147 (2019).

3 Vogg, M. C. *et al.* An evolutionarily-conserved Wnt3/beta-catenin/Sp5 feedback loop restricts head organizer activity in Hydra. *Nat Commun* **10**, 312, doi:10.1038/s41467-018-08242-2 (2019).

4 Zhong, Y. F. & Holland, P. W. HomeoDB2: functional expansion of a comparative homeobox gene database for evolutionary developmental biology. *Evol Dev* **13**, 567-568, doi:10.1111/j.1525-142X.2011.00513.x (2011).

5 Ryan, J. F. *et al.* The cnidarian-bilaterian ancestor possessed at least 56 homeoboxes: evidence from the starlet sea anemone, Nematostella vectensis. *Genome Biol* **7**, R64, doi:10.1186/gb-2006-7-7-R64 (2006).

6 Zimmermann, L. *et al.* A Completely Reimplemented MPI Bioinformatics Toolkit with a New HHpred Server at its Core. *J Mol Biol* **430**, 2237-2243, doi:10.1016/j.jmb.2017.12.007 (2018).

7 Huerta-Cepas, J. *et al.* eggNOG 4.5: a hierarchical orthology framework with improved functional annotations for eukaryotic, prokaryotic and viral sequences. *Nucleic Acids Res* **44**, D286-293, doi:10.1093/nar/gkv1248 (2016).

8 Miller, M. A., Pfeiffer, W. & Schwartz, T. in *2010 Gateway Computing Environments Workshop (GCE).* 1-8.

9 Letunic, I. & Bork, P. Interactive tree of life (iTOL) v3: an online tool for the display and annotation of phylogenetic and other trees. *Nucleic Acids Res* **44**, W242-245, doi:10.1093/nar/gkw290 (2016).
