# Supplementary material for: Molecular signature of an ancient organizer regulated by Wnt/β-catenin signalling during primary body axis patterning in Hydra
Source: Commun Biol. 2019 Nov 26;2:434. doi: 10.1038/s42003-019-0680-3 (PMC6879750; doi:10.1038/s42003-019-0680-3)
Supplement: Supplementary file 2 — Descriptions of Additional Supplementary Files [file 42003_2019_680_MOESM2_ESM.pdf]

Supplementary Data 1: Differentially expressed genes upon Alsterpaullone treatment.

Supplementary Data 2: Significantly differentially expressed TFs after Alsterpaullone treatment.

Supplementary Data 3: Differentially expressed genes upon *β-catenin* knockdown and Alsterpaullone treatment.

Supplementary Data 4: Differentially expressed TFs upon *β-catenin* knockdown and Alsterpaullone treatment.

Supplementary Data 5: **a**) Wnt/β-catenin signalling targets from Hydra, Planaria and Xenopus. **b**) Targets of Wnt/β-catenin signalling from Hydra categorized based on selected gene ontology (GO) terms.

Supplementary Data 6: *Hydra vulgaris* Ind-Pune Hybrid assembly_cds.fsa

Supplementary Data 7: *Hydra vulgaris* Ind-Pune Hybrid assembly Annotation.gaf

Supplementary Data 8: **a**) Primers used for cloning, qRT-PCR of target genes and ChIP qRT-PCR; **b**) Sequences of siRNA oligos.

Supplementary Data 9: Source data for Fig. 3b, 3f, 4c, 4d and Supplementary Fig. 7b.
